# Supplementary material for: Bioinformatics Approach to Identifying Molecular Targets of Isoliquiritigenin Affecting Chronic Obstructive Pulmonary Disease: A Machine Learning Pharmacology Study
Source: Int J Mol Sci. 2025 Apr 21;26(8):3907. doi: 10.3390/ijms26083907 (PMC12027559; doi:10.3390/ijms26083907)
Supplement: Supplementary file 1 [file ijms-26-03907-s001.zip › FigureS9 External dataset verification.pdf]

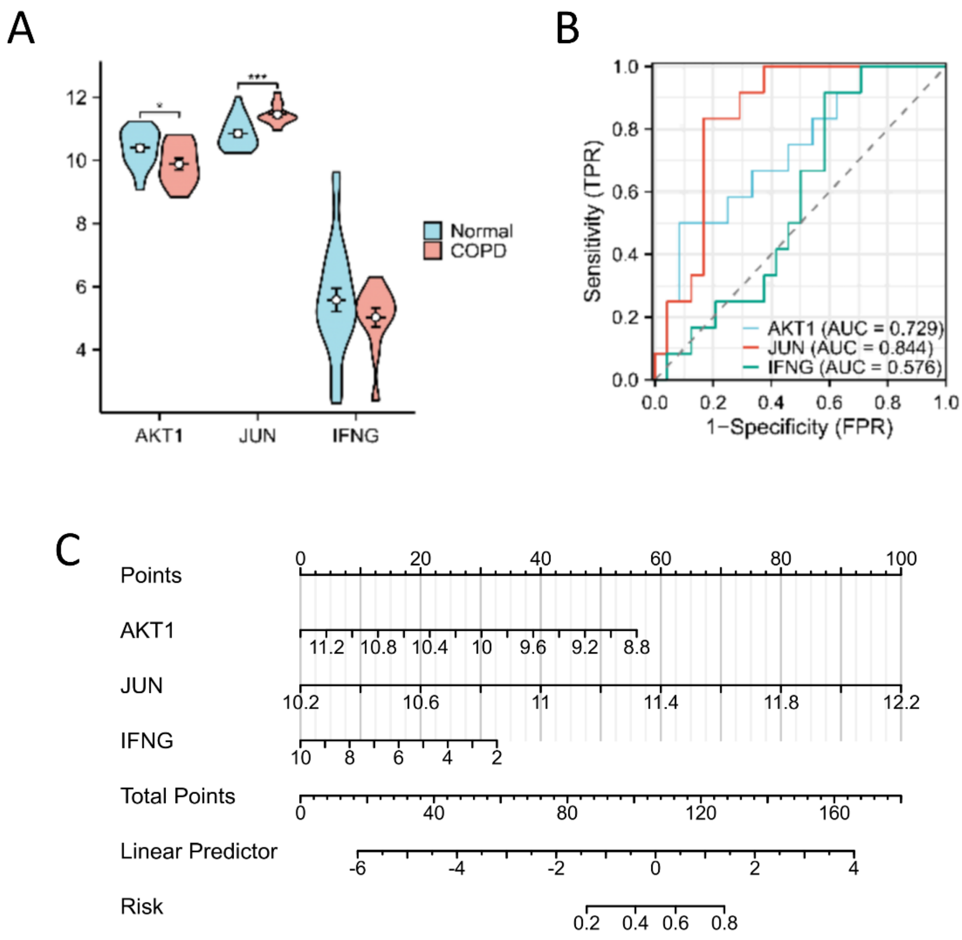

Figure S9 External dataset verification

A. Expression of key targets in GSE13896. B. ROC curve for evaluating the diagnostic efficacy of key indicators in GSE13896. C. Nomogram of key indicators for evaluating COPD risk in GSE13896

.
