# Supplementary material for: Bioinformatics Approach to Identifying Molecular Targets of Isoliquiritigenin Affecting Chronic Obstructive Pulmonary Disease: A Machine Learning Pharmacology Study
Source: Int J Mol Sci. 2025 Apr 21;26(8):3907. doi: 10.3390/ijms26083907 (PMC12027559; doi:10.3390/ijms26083907)
Supplement: Supplementary file 1 [file ijms-26-03907-s001.zip › tableS1.pdf]

Table S1 DEGs and key module gene lists

Only DEGs	Only WGCNA	DEGs AND WGCNA
HS3ST2	ACOX1	CXCL9
FABP3	AGRP	RSPO3
CCL2	APOL4	TNFAIP6
CCL23	APOL6	CCL20
HTRA4	ARHGAP22	MMP12
TRPC6	BATF2	HLA-DQB2
PLTP	BAZ1A	SLC19A3
ZMYND15	BST2	CXCL10
DDIT4	CBLB	RCAN2
TJP1	CCND2	ATP6V0D2
CTNNAL1	CD226	IFI27
mir-223	CD33	EMR1
AQP3	CD36	ELOVL7
RGL1	CD3G	LAMB1
CES1P1	CEP70	AOC3
PRSS21	CERK	SRPX
TMEM173	CHST15	NDP
FBXL2	CLEC1A	LPAR3
LGALS3BP	COL22A1	IL32
AK056982	CSF2RB	C1S
LGMN	CXCL11	DNASE2B
RASSF5	DCSTAMP	CCL4
LIMA1	FCGR2A	TNIP3
OLR1	FCGR2C	PROCR
EPDR1	FILIP1L	PRKAR2B
KIAA0101	FRMD3	SHROOM3
RAD51AP1	GBP1	FBXO15
LOC101930415	GMPR	SLC39A10
CPED1	GZMB	DEFB1
FLOT2	HERC5	MLLT11
SH3RF3	HLA-DPB2	IL1B
CRTAM	HLA-DQA1	GCH1
BNIP3	HNRNPA1	CADPS2
TMEM74B	IDO1	VNN1
RTN1	IFI44	HP
XK	IFIT1	EPS8
SNAI2	IFIT2	PTX3
FNIP2	IFIT3	MMP7
KRT10	IFIT5	FPR2
PRKCZ	IFNG	SLC26A11
MME	IL15RA	HSPA6
ETHE1	KLHDC7B	SMIM3
AICDA	LINC00996	TRBC1
IL2RG	LOC100132891	ADORA3
FCGR2B	LOC100506922	CYP1B1
ALDH1A2	LOC101927752	CD69
SELM	LRRC61	LOC731424
SERPINE2	MAP3K7CL	FOLR3
SCD	MATK	CCL5

TIMP1	ME3	AHRR
QPCT	MMP9	GBP5
TMEM9	MX2	INHBA
BCAT1	NEXN	LOC100996412
TAGLN2	NFE2L3	FCN1
CYP3A7-CYP3AP1	NT5C3A	TRHDE
IL4I1	OAS1	DFNA5
IFI35	OPN3	IGFBP2
C15orf37	P2RY14	TMEM163
CYB5R3	RARA-AS1	GPR34
FAM228B	RBM11	LINC01010
L1TD1	RPIA	CYBRD1
PRKACB	SAMD9L	SPP1
STARD10	SAMSN1	IFI44L
RP1-30M3.5	SP110	RASSF2
PIGW	SULT1C2	ITIH5
RNF19B	TAPBPL	FAM101B
CD84	THAP9-AS1	RP11-271C24.3
HRASLS	TM6SF1	PDE4B
RP11-38P22.2	TMCC3	TDRD9
MPV17L	TNFRSF21	PTH2R
ITGAL	TTC30B	SPRY2
MELK	UBE2L6	LOC100190986
PROS1	USP18	IFITM3
LINC00520	USP30-AS1	FAM198B
SIRPA	VAMP5	ICAM1
ENPP5	VKORC1L1	PLXNC1
TMEM192	VMO1	CD2
NCAPH	WARS	C8B
IL10RA	XAF1	CIR1
TSPAN6	YBEY	CD38
C1QC	ABCB5	KCNA3
C3	ACP2	CD3D
TMEM55A	ACSL1	ANKRD22
GRINA	ACSL5	AL833181
OPLAH	ACSS2	IRG1
CYGB	ADAM17	MCOLN2
STAT6	ADRB2	TDRD3
CD48	AF007147	TNFSF10
ANKRD28	ALDH5A1	CFB
LINC00969	AMICA1	MINOS1P1
PAPD4	ANPEP	AGPAT9
RP11-548H18.2	APBA1	S100P
AOAH	APOC1	RNASE6
SAMD13	ARL4A	GBP4
HCK	ATL1	CYTL1
TREM1	BACE2	SLC18B1
SHPRH	BID	PSTPIP2
PPAP2B	BIRC3	CXCL5
SOAT1	BTG3	DDIT4L
DRAP1	C11orf21	BCL11A

ZFYVE16	C11orf45	PMAIP1
TRPM4	C15orf48	C16orf54
SERINC5	C1orf162	TEX14
SPINT2	C7orf60	SLC22A4
SERPINH1	CCDC125	COL15A1
TVP23B	CCL1	GPR183
FAM168A	CD1D	KL
UBXN2A	CD40	TNS1
SLC25A39	CD44	AIM2
LRRC75A	CD55	TOB1
OMA1	CD58	RARRES3
STX11	CD80	SECTM1
ACER3	CDC42SE2	EPSTI1
SIGLEC15	CDK14	CTSK
RP11-305E6.4	CEBPA	HCP5
ACIN1	CELF2	BAG3
NDRG1	CH25H	AFAP1L1
TMEM194A	CHN2	SLC12A8
PVRL2	CIPC	IFITM2
CHAC2	CLEC4D	LOC644090
EDEM2	CLEC4E	PDCD1LG2
CA2	CMBL	SLC28A3
APP	CMTM2	SH3RF1
WFS1	COLEC12	ERV3-2
GRIPAP1	CTD-2165H16.3	LOC100131541
HIATL2	CXCL1	ASAP1-IT1
C6orf62	CXCL2	ITGAE
ARMCX2	CXCL3	TMEM14A
BTG2	CXCL8	CAMP
CORO7	DHDH	APOL3
TMA16	DHFR	LOC100129406
HLA-DPB1	DHRS3	ISG20
N4BP2L2	DOPEY2	GBP2
AXL	DPCD	SEMA4D
AKT1	DUSP6	IFITM1
MINK1	EBI3	TMEM158
PARP3	EIF2D	IL2RB
RAB24	ENC1	CCDC102B
SELPLG	ENPP4	CCR5
ZC3H12D	EREG	TIMP3
CHD9	ETS2	TCHH
IFNGR1	EVL	GALM
ATP2B1	EXTL2	PLA2G4A
TRANK1	FAM117B	CFD
VIPR1	FAM129A	PPP1R14C
GK3P	FAM13A	MYC
PIK3R1	FAS	FTX
PDLIM7	FBP1	SERPING1
MORF4L2	FHIT	GYPC
WDR54	FRAT2	RP11-403P17.4
SPAG1	FUT4	LIMK2

GUCY1B3	G0S2	TREM2
ACSS3	GADD45G	CHMP4C
PTK2B	GAL3ST4	STOX2
TMEM117	GALNT12	SMAD7
ACADVL	GPD1	LOC285812
TRMT13	GPR133	HCRP1
VASP	GPR64	STAP1
PLD2	GPR68	CDC42-IT1
CEPT1	GRAMD1A	SLC43A2
MAPKAPK2	H1F0	ID2
FAM223B	HADH	GAS2L3
TBC1D4	HAMP	MERTK
ZBTB41	HEY1	SLC20A1
GOLGA4	HIP1	GCLM
STIL	HIPK3	FHL1
MDFIC	HMGN2P46	PRO2852
ACSM5	HRH1	CNIH3
PKN1	HS3ST3B1	EHD1
EHF	IDH2	C2
KNSTRN	IER3	TRAC
BST1	IL10	LOC101926963
DERA	IL18R1	GCLC
LCP1	IL1A	VGLL3
SBNO2	IL7R	LOC101928716
RTN2	IMPA2	GPR84
SPNS1	IRAK3	FAM213B
DGKA	ITGB8	GAS7
ABHD8	JAK1	LINC01093
ZNF587	KBTBD11	NIPSNAP3A
ZFP36L2	KYNU	SCGB1A1
LOC101928000	L3HYPDH	TGFBR2
LPCAT3	LAMB3	MCOLN3
ZNF57	LILRA2	SLC38A6
ACAT2	LINC01410	ID3
VILL	LOC100505564	ANKRD29
MAD2L1	LOC100505592	RELB
TNFAIP8L1	LOC100506388	SHISA4
SIRT1	LOC100507535	GLDN
FMNL1	LOC101928429	TMEM45B
FAM102B	LOC101930114	CD8A
IRF9	LOC285957	CD180
CTSC	LOXL3	MX1
DZIP1L	LPAR1	GZMA
FANCE	LRMP	RNF144B
PDE9A	LRPAP1	PKIA
KDSR	LTB4R	ZFAND2A
GK	MAP3K8	CKS2
CTSH	MARCKS	SIGLEC1
RFX5	MARCKSL1	SSBP3
UBTD2	MB21D2	TRERF1
KIAA0226L	MBOAT1	ARHGAP24

WDR43	MGAT4A	DMXL1
TAF13	MGC12916	ASAP1-IT2
MXI1	MMD	IGSF6
IL17RA	MMEL1	MT1F
TM9SF4	MS4A6A	IRF7
CDK2AP1	MT1E	ALDH18A1
EVI2B	MT1G	PSMB9
DNAJB5	MT1H	ACVRL1
FLOT1	MT1HL1	SGTB
ZNF587B	MT1M	PNRC1
PSD3	MT1X	A2M
GUF1	MT2A	RBP7
PPIC	MTF1	ICOS
CSTB	MURC	CPE
USP21	MXD1	LOC285181
MVB12B	MYO1G	BEX5
KIFAP3	NFIL3	GZMH
LINS	NFKBIA	THAP10
IARS	NQO1	ZC3H12C
ZBTB8A	NREP	SLC6A16
SRGAP1	NRGN	ITGAM
PILRA	OR52K3P	GTF2H3
CDK6	OSBPL11	TCTEX1D2
GIPC1	OSBPL1A	PMEPA1
ALDH2	OTUD1	TUT1
LOC100287896	P2RX1	FOSL2
SEPHS2	P2RY12	C1R
TBCCD1	PAQR8	TNIK
PLA2G7	PBX3	SNX7
PVR	PC	RAB42
CCDC43	PCED1B-AS1	LCK
C2orf76	PCNX	FAM26F
STEAP3	PDE4DIP	LGALS3
ZBTB21	PDK3	PRKCB
GCAT	PEA15	SOCS3
HLA-F	PLA2G4C	SKAP1
ASAP2	PLGRKT	DBF4
RRP15	PNPLA3	MLF1
LINC00888	PRADC1	FAIM
ZNF702P	PRAM1	HPGD
AP5B1	PRDM1	LRRC69
ZNF155	PRDX4	PRR11
AMOTL1	PRKX	MS4A6E
TMEM33	PSRC1	GTF2H2B
GALC	PTGER2	OSM
RORA	PTGS2	ZBED3
C12orf66	RAB8B	DNAJC5B
TMEM99	RARRES1	SLC39A8
ATXN2L	RASGRP1	A4GALT
GNG12	RBP1	ACKR3
TMEM251	RGS18	PDLIM1

RAB4B	RNASE1	AK090844
RPP40	RNF125	PPAP2A
GDF15	RNF166	DSERG1
KIAA0232	RTN4R	MATR3
PANK3	RUFY3	CD274
CNIH1	SCAMP1-AS1	FLJ31306
ETV6	SCCPDH	ALK
POLM	SDC4	LOC101928770
RRAS	SEMA3C	ISG15
PPP1R13B	SEPP1	NLN
RAB27A	SERPINB2	SLC48A1
PBX4	SESN1	C20orf194
ADAMTSL4	SGK223	AKT3
SEC24B	SLAMF7	KIAA0930
MRPL19	SLC16A6	SETDB2
TUBB	SLC1A2	ENTHD1
AZU1	SLC2A6	TCF15
COPS7A	SLC37A2	SLC26A6
PSD4	SLC41A2	LOC100272216
ZDHHC23	SLC44A2	TPD52
SESN2	SLC45A4	RASGRP3
MOV10	SLC46A1	TCEB1
DAPK1	SLC6A12	STAT1
SIVA1	SLFN11	ANKRD10-IT1
CD74	SNX10	ACP5
COL4A3BP	SPATC1	MRTO4
DYRK2	SPRED1	APOL1
AP1M1	SSR1	ZFAND6
ARL2BP	ST5	JSRP1
NUP35	STMN1	BTN3A3
IGSF8	SUCNR1	TGFBR1
C8A	TANC2	RND3
MAP4K1	TFCP2L1	CST6
CCDC159	TLE3	NAA25
PLA1A	TLR2	AOX1
RP4-710M16.1	TLR4	METTL9
MFSD10	TMEM37	NR1D2
CDC23	TMOD3	LRG1
C1QB	TNFAIP3	PHACTR1
FAHD1	TNFAIP8	MAP4K4
C8orf76	TNIP1	PLIN2
ZNF274	TNNI2	LOC102724356
MCL1	TPD52L1	IDH1
RAB32	TREML3P	RNF19A
GPR137B	TREML4	NEIL2
LOC102724965	TSHZ1	ASRGL1
CCNB1IP1	TSPYL5	GPNMB
FAM134B	UBE2Z	LILRB5
SLBP	UXS1	RRN3P1
CCDC152	VCAN	KIAA1147
PTPRJ	VWA8	FABP5

SPAG5	WDR91	ZNF426
RP11-589P10.5	WDR92	ZNF646
AK8	WFDC21P	CA12
OAS3	WTAP	LOC340340
FAM98C	ZMAT3	MOSPD1
ARF3	ZNF618	IGFLR1
LOC146880	ABHD17C	SLC16A10
MDM2	ABO	BTN3A2
SLC35A3	AC004941.5	WWTR1
LIPA	AEBP1	AX748339
TRIAP1	AF213884.2	SRI
NBEA	AL928742.12	PSMB8
P2RX4	APOBEC3G	NNT-AS1
TRIM21	AQP5	CCDC91
CMTM7	ASB16-AS1	CFP
SS18L1	ASGR2	LOC284837
SPRYD4	AVEN	RP4-781L3.1
SRGAP2	BAI1	CCDC151
SLC25A11	BBS5	MAP1LC3A
ZAK	BPGM	PRPF3
LSM2	C11orf80	CD163L1
FLVCR1	C16orf74	OAS2
NEK4	C9orf37	COL23A1
C1QTNF9B-AS1	C9orf50	EIF4E3
SLC41A1	CCDC114	AK025288
MBIP	CD320	PDPN
SBF2-AS1	CHRNG	GPD2
RP11-334J6.6	CHST8	ARNTL2
PCNA	CLCN5	C1RL
C2orf69	CNIH2	RASAL3
IL4R	CPNE7	HNRNPU-AS1
SPRR4	CRB3	RRAGD
SYP	CRIPAK	NFKB1
EPB41L4A-AS2	CRYBB2	KCNJ5
AX747507	CSF3	RP5-894A10.6
ARHGEF11	CTB-31O20.2	RHOU
ERLIN1	DHRS13	SCT
TXNL1	DQ570835	RP5-1074L1.4
TNFRSF1A	EIF3C	OTUD6B
SCGB3A1	EMILIN1	RACGAP1
MYOZ1	FAM71A	EPB41L3
DPH3	FHL3	NLRC5
CCDC62	FIGNL1	DENND4C
PSMG1	FLJ20021	NPL
IFFO2	FUT5	SOCS2
ARRB2	FZD9	SLC29A3
NUPL1	GJA8	PKDCC
CRELD1	GJD4	DAB2
RPL37A	GNG8	LGALSL
QSOX1	GNRH2	LSS
CELSR1	GRASP	LOC101927098

ODF3B	GS1-124K5.11	TMTC2
DCLRE1C	HERC6	OR2A4
UBE2Q2	HIST1H2AJ	KANSL1
TNNT1	HMX1	ORC6
C12orf5	IL17B	LOC340184
EPN1	INTU	LOC645984
C1QA	ITPR1-AS1	CCDC115
CTF1	KANK3	RGS1
TRPC4AP	KCNF1	RHOH
SH3BP5	KCNQ1DN	BLVRB
CPM	KLHL28	MIR3916
ZYG11B	KRT81	ZDHHC2
AF520793	LA16c-381G6.1	ZZZ3
KIAA0040	LAMA5-AS1	ETS1
SUMF2	LILRA4	LINC00597
MKKS	LIME1	ZNF468
APOPT1	LINC00115	MAP1LC3C
IGF1R	LINC00685	LOC286437
SPOCK2	LOC100128108	LOC101927451
PTGER3	LOC100287808	SNX20
KIAA0922	LOC100507557	FAM63B
ADPGK	LOC100652768	ABCC5
GMNN	LOC101927365	ITPK1-AS1
SRF	LOC101927507	TAF15
SAMD9	LOC101929076	TMEM69
SMNDC1	LOC101929609	CD82
HLA-B	LOC101930026	PDE1B
ZNF436	LOC102606465	IFT57
MCUR1	LOC153682	GFOD1
LTBP4	LOC284242	PSMB7
TTC37	LOC403323	PARP9
CXCR3	LOC643072	IGF1
SS18	LOC644656	S100B
TMEM68	LRP8	PCOLCE2
CPEB4	LRTM2	MMP14
MORC4	LY6G5C	HEATR3
OSBPL5	LYSMD4	EPHB2
FAM46A	MANEAL	MMP2
NDUFB9	METTL2B	CASC14
SLC39A6	MGC50722	RAP2B
DRD4	MIRLET7D	HOXB6
MIR1244-3	MIS18A	SLC8A1
SLC6A18	MYSM1	LOC100127972
MRPL43	NANOS3	ZFYVE26
ARID3A	NBEAL1	ATP6AP1L
FAM73A	NKPD1	SPECC1
AGPAT1	NPAS1	CLIC4
FMO4	NPPA	LPAR6
PRAF2	NPPB	PALLD
EPB41L4A	NUP155	CD163
MED13L	P2RY1	CCZ1B

UBAP2	PDE3B	CREB5
GPR27	PGLYRP1	PLEKHA4
ACTA1	PIGZ	LMO4
PRKRA	PLEKHN1	LOC100132167
FAM214A	PLIN4	XIAP
TMEM243	POP1	LINC00622
GSR	PRDM12	SPATA12
FMN1	PRKY	CP
POMT1	PRLH	TRAPPC10
YWHAQ	PRR3	RP11-182L21.5
C4orf29	PTGES	PRIM1
NOP58	RALY-AS1	PSMC6
PRMT3	RNF43	SLC19A2
NT5C2	RP1-170O19.17	CYP11B2
RBM4	RP11-199F11.2	LOC101927974
MLLT4	RP11-389C8.2	C15orf61
WEE1	RP11-465B22.8	PSMB8-AS1
SUOX	RP11-554J4.1	MAN1B1-AS1
PPP3CB	RP11-585P4.5	ZNF573
FAM195A	RP11-796E2.4	PTMS
MTX1	RP11-930P14.2	PTGR1
MAFB	RP11-97C16.1	UCHL1
RPS6KA2	RP13-270P17.3	REV3L
RELL1	RP3-368A4.6	NEO1
NSMAF	RP4-612B15.3	PMFBP1
HS2ST1	RPS24	IRF1
TSR2	RPS6KA2-AS1	SET
GLYR1	S100A1	TMEM123
MALT1	SCARNA15	C12orf4
GPALPP1	SEC31B	GPR171
NOL3	SH3BP4	CECR6
SIX5	SMARCB1	FAM110B
ALG8	SMIM1	LOC101927150
VCPKMT	SNHG19	TNFSF8
DIAPH2	SPEF1	SEC24D
RPS27	SRD5A1	DAXX
LOC153684	SRRM5	SFMBT2
LTA	STRBP	HOXB7
RP1-102H19.8	TAPT1-AS1	SARAF
PSME1	TBC1D19	SRCRB4D
OVGP1	THUMPD3-AS1	NFKBIE
UBASH3A	TMEM179	FBRS
HLA-F-AS1	TMPRSS4	CBR3
GTF2IRD1	TTLL1	KCNJ1
TATDN1	TXNL4B	SLIT2
ATP6	USHBP1	UBTF
WWP1	ZBTB6	AGRN
TRAPPC13	ZCCHC4	ZNF207
DYRK1B	ZDHHC13	SCAF1
MAP3K3	ZNF566	TMEM151A
ZNF562	ZNF767P	SMA4

TNNI1	ACSL4	CLSTN1
MRAS	ANP32A-IT1	DLEU2
MED4	AX746823	KLHL21
ZNF529	BC022892	CEBPZOS
PUS7	BMS1P5	CADM1
LTK	CCDC88A	LOC102724017
LCORL	CORO6	ROBO1
LFNG	CTB-181H17.1	C5orf28
CNPPD1	DNAAF2	PHAX
TMEM38B	EP300	TMEM255A
SRP9	GTF2F2	TMEM138
EXOC6B	HYMAI	LINC01000
DEDD	KCTD21	KPNA2
FAM217B	LIMS1	DENND2D
ACTB	LOC101928806	HAND2
THEM4	MGEA5	TTC39B
LA16c-380H5.4	MZT2B	COMMD8
BC047484	NADK2	CCPG1
SLC4A8	NPDC1	AP1S2
PSMA7	NPTN-IT1	ADAM28
LIN52	NUMB	APOOL
DUOXA2	PELI1	HIBADH
TBCE	PMS2P5	BMS1P6
KCNK6	PPP3R1	RP6-99M1.2
MYH9	PRPF39	CXCR6
APPL1	RFWD3	CLTC-IT1
MAPRE3	RP11-138A9.1	GBE1
STXBP5	RPA4	AGAP4
UBXN8	RPL36A	TP53INP2
GAS2L1	SEC14L1P1	ACOT13
LOC283335	TMEM44-AS1	XYLT1
GPC2	TTC8	TCF7L2
SUPT6H	ZNF329	KDM1A
USP54	ACRC	WDR11
HS3ST1	ACTRT3	AC068039.4
PSMD7	ACYP1	KBTBD8
TMPO-AS1	AEN	ZNF430
CDKN2A	ALG12	TBC1D9
EIF2A	ALMS1-IT1	SNORA21
JAK3	ARC	IFNA1
GORAB	ARL5B	FLT1
LINC00174	ASH1L-AS1	ACTL6A
DNAJB6	BRAF	LCOR
PSMD14	BTBD3	FUBP3
TBCB	C12orf50	CMC4
ALG14	C14orf182	H2AFY
BTNL8	C1orf233	NLRC3
EFNB1	CCDC30	RP11-339B21.15
FERMT3	CD99P1	LINC01137
TUBA1A	CHCHD6	C6orf211
FAM110D	CHRNA5	PPIP5K2

DAAM1	CLK1	WDPCP
NONO	CMPK2	CENPL
SERAC1	CNN2	CSGALNACT2
RNF39	CNST	C19orf33
PCBD2	CRYAB	TMEM102
SMAP1	CTD-2286N8.2	LOC100289333
BTBD6	CTH	JUN
ITFG2	DDX60L	CHI3L2
EXOC3L4	DEPDC1B	DENND3
CXCL16	DLEU1	KIAA1715
LOC284926	DNAJA4	DENND5A
GRK1	DNAJB1	AY940074
DCAF15	DNAJB4	RETSAT
LARP6	DNAJB9	MVD
BGLT3	FAM126A	SWAP70
ZBTB22	FCHO1	GZMM
LOC101929177	FEM1B	LOC441124
CCT6A	FEM1C	STAT2
TERF2IP	FGD5	IQSEC1
SRPX2	GCNT3	RP11-998D10.7
SEPT8	GID4	KIAA0907
NUMBL	GNE	ATP9A
MICU1	GPAA1	PTPRCAP
OS9	GSDMD	CBLL1
SLC38A7	GSTZ1	HEATR5B
TRAF2	HES1	BARX1
ZFAND1	HIST1H2AM	XRCC2
CCDC71L	HMGCS1	NFAM1
TAF7	HSPA13	RP2
ZDHHC5	HSPA2	HSCB
UNC13D	HSPH1	TAP1
GLA	IER5	SCUBE1
E2F6	IGF2BP3	GBAS
SLC30A7	IKZF5	TMEM91
FOXD3-AS1	INSIG1	FCAR
AC005523.3	ITCH	IL34
MTA1	KATNAL1	ZCCHC7
FAM134C	KIAA1804	MEF2A
HEBP1	KLHL24	STT3B
ELMO1	LAIR1	NKG7
C11orf73	LAPTM4B	NLRP12
PDGFRB	LDHAL6B	IFRD1
COPRS	LGALS1	TUSC5
MRPL3	LGALS9	LOC100505715
FBLN2	LILRB1	THEMIS2
SNX29	LILRB4	HES2
DBT	LINC-PINT	LYPLA1
EFNA4	LINC00116	CD1B
PGLYRP4	LINC01181	RP11-379F4.6
F13A1	LOC100132735	SGK1
UPF1	LOC100134822	LOC158960

NTAN1	LOC101928487	C10orf35
PPM1G	LOC101928894	MAP4K3
FCER1G	LRCH1	TDP2
ARHGEF19	LRIF1	AGPS
TSPAN10	MASTL	RAPH1
EIF3J-AS1	MEP1A	PDE4A
TBCA	MTUS1	CMTR1
FBXL6	N4BP2	TRIM33
LOC100128644	NDUFS3	RPS16P5
GATC	NMT2	RCBTB2
SLC17A5	NR2C2	ARMC5
ADCY8	NT5C3B	GNG10
LOC100506730	NUTM2A-AS1	NUDT19
CCDC84	OGFRL1	TMPO
FUZ	OTUB2	RP11-1094M14.11
NAPSA	PCOLCE	XCL1
ARF5	PDK1	TBC1D24
CD7	PIGA	NRD1
EI24	PIR	FLVCR2
PLEKHF1	PLA2G16	NXF3
NAT10	RAB30-AS1	CFDP1
C22orf46	RGS2	GTPBP2
MAN2B1	RHOBTB3	NIP7
RP11-288H12.4	RLF	PITPNA-AS1
C21orf91	RRAD	FAM63A
STAM	RRN3P2	TRIM17
TIMM9	RSAD2	SEPT5-GP1BB
MANEA	SIRPB2	SLC25A13
ZNF492	SLAMF9	ZNF721
UQCRC2	SNX16	ITPKB
ZNF493	SOD1	RP4-773N10.4
CEMP1	SPINK1	SEMA4B
CFL2	STON2	LACTB2
SNAPC2	SUMF1	AP006222.2
GPR85	TAF1A	RP11-690I21.2
ADAR	TAF1D	NOD2
CKAP5	TDG	HK2
SLC22A3	TIMM23B	NAB2
SCNN1B	TLK1	CLN8
RAB18	TMEM191A	KMO
MRPL32	TMEM205	RILPL1
PATZ1	TNFAIP8L2	BC033164
ONECUT3	TSC22D3	ZBTB43
KIAA1875	TSPYL2	GRAMD4
IL12RB1	TTC4	ST20
PRR19	TUFT1	KCTD9
ZNF282	TWSG1	CHUK
PPP1R13L	UHRF1BP1L	SCAF4
CES3	UNC93B1	ARHGEF1
RP3-486D24.1	WBP4	RPPH1
WRB	ZNF281	RP3-522P13.2

B9D2	ZNF354B	LINC00657
C2orf16	ABCB10	PITX3
PROC	ABRACL	TDRD7
ODF2	ACADM	UBLCP1
BAG5	AGL	CELF6
ALMS1	ANXA2P1	COX17
PINLYP	ARFGAP3	ZBED5-AS1
FHDC1	ARL4C	WTIP
EEF2	C6orf57	SNX8
FAM229B	C9orf64	DNAJC6
RASEF	CHMP5	LINC00339
TUBA4A	CMTR2	C14orf142
LOC100507156	CTC-459F4.3	DUSP4
KLF7	DBR1	RPL7AL2
MS4A7	DCK	CMAHP
CILP	DEK	POMP
MPG	DHFRL1	CPOX
TMEM14C	EIF1AX	KATNBL1
PODXL2	EOGT	B4GALT1
DHX35	FAM174A	LOC100506119
ULK1	FPGT	UBXN7
TCF12	GINM1	ARAP3
MED31	GOLGA2	ISOC1
MAVS	HEATR5A	TIAM1
LOC285095	IL18	DGKH
EIF2AK4	KAT2B	GLTPD2
NGLY1	KTN1	SLC44A1
CD79B	LAMP2	PPP5D1
LTB	MAN1A1	LINC00028
TRPM5	MAP4K2	RP11-846E15.2
CREBL2	MGC70870	RIN1
ZBTB42	MITD1	PLSCR1
SLK	MOB1B	CTSS
C20orf141	MORC3	CRYL1
NRM	MSMO1	GLIPR2
CISD1	NCEH1	KLHL42
PIK3AP1	NOTCH2NL	C19orf68
DNAJC9	PARG	FBXL19
PBXIP1	PLAGL1	IGFBP7
NUDT12	POC1B	TPCN1
FAM13B	PPIL1	KRT3
XRCC3	PPM1B	SSC5D
ZC3H18	PRICKLE3	PKD2
LOC101060691	PRKAA1	ZHX1-C8orf76
CDC42EP4	PTGR2	LTB4R2
DOHH	RAP2C	LINC00900
BRIX1	RECQL	RP11-6I2.3
ZFP36	REEP3	PLEKHG4
RAB11FIP5	RP11-488L18.10	C9orf142
LOC100289098	SACM1L	ROM1
SORT1	SCP2	RNF11

DCAF13	45915	SPAG9
ARPC1A	SGMS1	NABP1
ZNF611	SLC25A43	NDUFA5
AAMDC	SPPL2A	ATG16L2
CLVS1	SRD5A3	ST6GALNAC6
SP2	SRFBP1	AC005256.1
LOC647070	SRSF11	GGCT
NEK10	SUCLA2	RASA3
TEFM	SUCLG2	SLC25A33
SDR39U1	SUGT1	APOA4
CLDN7	THOC7	MYO6
ZC3H8	UBE2D1	POLR1B
FBXO44	UFL1	TRMT2B
PIK3IP1	USP32P2	CLSTN3
DCUN1D4	VPS41	LOC100133130
CD164	VPS4B	HES6
C17orf59	WBP5	FAAH
B4GALT7	WDR47	RGAG4
PPM1N	ZNF217	ZFP92
ST3GAL4	ZNF267	CUL2
NAA50	ZNF366	LOC340085
ADPRH	ZNF518A	LINC00998
VPS13C	ZNF862	LAP3
IREB2		SETD1A
LINC01231		METTL7A
USP34		EID3
CLDN5		SEC24A
RP11-209A2.1		REXO1
HOPX		CRYGEP
C19orf70		FMO5
FGD6		PSD
PDGFA		GLIDR
CYTH1		XPO1
C16orf87		DUX1
FAM171A2		ALKBH4
CCDC50		MPC2
FLJ38717		GPR144
B3GALT6		VCL
TPPP3		TUBB2A
C12orf49		HCG26
ASMTL		SEPT1
SH3BP1		MYLIP
PEX10		BC044596
NADK		BTN3A1
ABCE1		RP1-118J21.25
TFDP2		FCF1
AP006216.10		FMNL3
SPR		PYY2
MKNK2		DLG4
TOR4A		TBL1X
ASPDH		ATP8B2

MARC1
STRAP
NME3
SLC36A1
ZKSCAN4
ATG2A
FLT3LG
TAOK2
EMC3
GBGT1
LBH
MAN2B2
COPE
PDGFD
CDIPT
RDH5
CALY
ANAPC7
ST3GAL2
HDGF
G3BP2
PPIL4
CCR10
ACLY
ARSD
SNPH
PKIG
PINK1-AS
H2AFZ
BEST1
ADH5
EPHB4
LOC102723661
TMA7
SZRD1
LOC101928476
C1orf109
MGC12488
ATHL1
RUVBL1
C14orf80
NMUR2
MTFR2
NUP153
PDCD10
PYDC1
CAMSAP3
CTD-2008P7.1
LOC100996255
FAM96A
LAMTOR5-AS1

ZBED6CL
COMP
TFEB
UBA2
STXBP3
LPPR3
USP13
DGKG
MCM3
ARID2
FNBP1
SVIL
NEBL-AS1
ABCC4
MGC10814
FNIP1
OPTN
LOC100287497
IGFALS
C1orf122
RP11-61L19.3
EMC2
AKAP5
KRBA2
C9orf172
CPNE3
CHST13
ARHGEF5
A1BG-AS1
C1D
NSF
KCNJ10
LYPD5
ZNF654
KIAA1919
CA5BP1
SLC38A9
EGFL7
BLOC1S6
CDC42EP2
PPP1R18
ETNK1
TSFM
INPP5F
HLA-DOA
PLBD2
TEX2
RB1CC1
LPP-AS2
ABHD4
CDRT15

NAB1	MICU2
PSMB4	PSMD2
NR5A1	NSUN6
C11orf72	LRRC16B
LOC100130428	POMC
BCL9L	TMEM262
NACAP1	NFKBIL1
MLLT10	GBP1P1
DDAH2	RP11-305K5.1
ABHD16A	ROMO1
ACD	UBALD2
ZNFX1	ITGAX
LOC101928378	FAR1
VPS8	CD247
MTRF1	TRIM52
PRKD2	RP11-272D12.1
HDDC3	VDR
PTPN3	SND1-IT1
LRRC40	ATAD3C
LOC101929681	LOC100289230
TRAPPC6B	OR7E156P
MGST1	RRN3
CCM2	NKX2-8
LOC101926967	SIMC1
MLLT6	ALDH3A1
LOC100287098	GNLY
ACAD8	TSPAN32
ZSWIM8	PNOC
OIP5-AS1	EGLN2
MIPEP	COL9A3
FARSB	CHORDC1
LSM12	RNF135
SNRNP200	PHYH
PNPLA2	ZCCHC6
MTMR3	FRY
SLC38A1	LOC100131170
BROX	TMEM57
PGF	FBXL8
SLC5A8	ZNF277
BC039122	CEP68
LRRFIP1	ANTXR2
USP46	CPEB2
IGLL1	FURIN
ATF5	FAM3D
SEPT7	RPS23
ADAMTSL2	LOC646214
ZW10	SPATA25
SMC4	CYP2W1
ASCC1	EFR3A
PRKRIR	RNFT1
SLTM	ADCK5

CEBPG
ABR
LRRC10B
CCNDBP1
GTF2A2
HCLS1
DPM1
RNF220
YTHDF3
IGF2BP2
LOC102723845
LMNB2
GAS5
CCDC178
DNAJC5
EMC10
TPCN2
RIOK3
MZB1
BAHD1
LOC101927420
TMED10
SRPRB
PHKB
VGF
RUSC1
TADA3
SEC24B-AS1
RTN4RL1
OSGEP
FRMD4B
TYK2
FAM161B
PARP12
LOC100505540
KLHDC9
PLEKHG3
ANO6
EFEMP2
DRAM1
GS1-124K5.9
RP3-334F4.1
BRPF1
MTPAP
ZNF763
ITGA10
PREX1
PRSS36
RPH3A
LOC100505478
CCL17
CRY1
RP11-436D10.3
GPATCH2L
OCEL1
EBLN2
AC005306.3
ARF1
CELP
ADAT1
RELT
CLDN9
TTC36
VEZT
ADAMTS14
FAM210A
PHACTR4
LIMD2
TOPORS
LOC101927330
LCN1
CAMSAP2
LINC00176
GDF2
ROBO3
IFI27L2
AFF4
TPST2
TTI1
HEXIM1
GUCA2A
VRTN
IL13
KCNG2
BCL6
NAA16
LOC101927278
ZBED4
LOC101060510
IGF2-AS
HSF2BP
CLIP4
CHD2
LTV1
TLX1
WAC-AS1
RAB13
NDFIP2
PERM1
SIPA1L2
ANKS3
DEGS2

ELK1
NUDT15
HLA-E
RHEBL1
PIGO
CSPG4
ADAM19
PRKAR2A
SIN3A
HGC6.3
AX747031
TCAIM
MGAT3
EPM2A
ZNF687
LOC100134040
C8orf60
KAT8
STX6
MPV17
CXCR5
HHIP-AS1
TPBGL
GPR25
ATXN3
CCAR1
ST18
FAM13A-AS1
USP31
XRCC1
NXN
MEI1
KIAA0754
CRKL
EMC6
MED6
RGMB
RNF26
TREML1
CC2D1A
PFKL
RP11-1191J2.5
APH1A
RABL3
LOC100506713
ATP6V1E2
LOC101929284
PLEKHB2
SLC25A17
RPL32P3
POFUT1
LENG1
NUP98
CAPN2
FBLL1
ZNF883
MIR34A
PXN-AS1
LOC157860
CELA2B
RP11-432J9.6
FAM126B
DLL4
ABCA7
ABCA1
ASPH
IFNGR2
SIK3
C3orf62
PODNL1
ZNRF3
MYH13
RP11-727A23.11
RP11-3304.1
ZNF205
PWAR6

IQCC
REEP1
CCNI
ZNF701
CYP2B7P
IER2
SLCO3A1
MECR
RBFOX2
RBM4B
ZNF683
FPGS
MMACHC
KLHL6
EIF3E
LIX1L
ABHD3
FRS3
TONSL
PITRM1
DSE
ZNF696
NT5C
CIDECP
SETD9
AMIGO1
MOAP1
STAG3L3
FLYWCH2
TLE6
TIMM8A
DVL3
OSTC
BCL11B
CYP4F12
CCDC106
MRPS25
LRFN3
CTC-338M12.4
FAM186B
CENPW
RNF130
SPATA18
LOC284023
JAKMIP1
USP14
SUV420H2
PPP1R3E
REPS1
GOLT1B
BTN2A2

CTD-3028N15.1
TTTY11
SCPEP1
HLA-G
DIXDC1
TRAFD1
OR8G1
BEX4
AX747730
LOC100507540
RAB31
ZDHHC16
ALKBH5
TCERG1
SGPP1
HK1
SLC38A5
CEBPE
TMEM238
RP5-892K4.1
MSI1
VKORC1
AC016999.2
DDX24
LINC00294
PSMB2
RP1-20208.3
MRPL18
MFSD11
LOC101929718
NUDT22
NALCN-AS1
ZNF334
SRSF4
DENND1B
CTD-2083E4.7
LSAMP
OTOR
SOCS1
NYX
FOPNL
FNDC8
EDN2
RP1-199J3.7
RGS14
PDS5A
ERCC4
LIM2
BBS2
THUMPD2
SERINC1

NOTCH3
HPS3
EIF3J
SLC7A6
AOC1
USO1
PRKCH
PIP5K1A
ANXA3
ARL9
LOC101928134
MSRB2
ZNF671
NPW
TLR10
RBM47
MRS2
RNASEH2A
RP11-203B7.1
F2
PEX5
CIRBP
CDH1
ACAP1
SUGCT
SCML2
RFX2
ATG4A
AC004692.5
SLC25A24
DSC2
PCDH1
DNAJC4
SRP54
LOC100287525
PDE7A
PAN3-AS1
PITPNC1
EIF3M
GATM
RPF2
LIN7C
CRIPT
NEUROG3
SPRYD7
PAIP1
MRPS35
HMCES
ADIRF
XPO5
TMEM125

ZNF358
NMNAT1
PDZD7
CDX2
UBE2J2
MTIF2
SLC16A11
FAM106A
RANBP2
ZCCHC10
USB1
BSN-AS2
HDGFRP2
RAD23B
MTG1
OTUD5
ELMOD3
LRP1
RP11-66N11.8
UPK3B
RDM1
RNF170
VIPAS39
GALNT2
LINC00705
CHMP2B
LINC01049
LOC441461
C9orf85
UNK
NOD1
CEP83
PRUNE
SYNGR2
TPST1
SPRYD3
NAGS
CYCS
RP11-109G23.3
ADAM33
KLF16
ARNTL
TNRC6C
FAM207A
HSD17B10
MAMSTR
ARL15
FGFR1
GUK1
BLOC1S2
PRELID1

SSR3
SYNJ2
RP13-20L14.1
FAM208B
LRRC47
MLXIPL
PSMD6-AS2
COMMD4
OGFR
SLCO4C1
FGD3
RP11-79P5.2
NFE2L1
CEP128
TMEM219
RGL4
UHRF2
HOXB4
SMURF1
KPTN
PPP2R5B
EML3
MEF2D
TM7SF2
AC074212.6
HBB
OSBPL2
ZNF229
TXNRD1
CLDN14
FAM91A1
MRPL33
TRMT12
PLEKHG4B
CCDC28B
DUS2
RP1-130G2.1
ALAD
NPC1
IL13RA1
ZMYND12
TSPAN3
PDCD7
METTL8
LOC151174
HSPA9
MOSPD3
C9orf53
GTF2B
MTDH
RTKN

LOC101927534
FBXW12
EBF4
PHYHIPL
FABP2
SURF2
TG
FLJ42627
PTP4A3
LOC101928787
ARFGAP2
CYFIP1
DHX58
RP11-235E17.4
DOK1
NME6
CNR2
ST3GAL4-AS1
CCBL2
LOC286009
TRIM54
SIRT7
SLC2A1
KLHL7
GRAP2
AFG3L2
SHB
ZNF418
BTBD18
TMEM65
ACBD4
DCLRE1B
PLEKHB1
SEMA3B
FAM86A
APOL2
AP4S1
CNIH4
FAM3A
RDH11
KMT2B
LOC101927620
ZNF736
CDCA4
ASNSD1
LRRC45
BCL2L11
LINC00544
DUSP9
MAP3K5
XPR1

UBL4A
MAPK8IP3
NKTR
SEPT6
NKX1-1
ZNF765
LOC101927513
TRIM24
TRIP12
CCDC127
PNISR
BICD1
LNX2
SCOC
TNKS2
LOC102725017
BACH1
PPP2R4
ZNF574
LITAF
DCBLD2
TESK1
PPA2
COL16A1
ATP6V1C1
SEC61B
JTB
CRCP
CTD-2587H24.10
POLRMT
PPP1R9B
PDGFRL
YTHDF2
KIAA0247
DCAF10
NUP210
LINC01359
ADI1
GGACT
COA7
HTATIP2
PDIA2
ADRA2C
ADSL
PCBP4
ZNF75D
HYI
ANKRD2
LINC01082
HOXC8
GRIN2D

SLC35E1
CIDEC
CD96
EVA1B
PHF21A
CST2
MYBL1
NAALADL1
CNTROB
ZXDC
ZNF431
CD4
DNAJB13
WDR77
ETV2
PPP2R5E
COG6
LOC285191
SPACA4
OR51B2
TBC1D7
ZBTB11
PTRH2
SLC25A28
ANKHD1
RBP2
GGNBP2
RP11-195M16.3
SPATA5L1
C14orf166
PDYN
NPM1
CRYGC
NOTUM
MOCS1
RP11-498C9.17
MRPL37
POGZ
ERVW-1
LRRC71
PSMD12
AC025442.3
RP11-338N10.1
CCDC63
TMEM101
PTDSS1
CDCA8
MROH1
PQBP1
LOC101928635
SPRY4

FAM49B
EPS8L1
SMAD4
MBD4
IDUA
LOH12CR1
SSRP1
TMEM144
NCOR2
GNA12
RFPL3
BC070118
GBX2
EPHA2
ADARB2-AS1
KIAA1279
PI16
ITM2A
CENPN
LOC101929406
RBM10
ERGIC2
WDR81
CECR5
C3orf38
RNF13
TUBB7P
CELA3B
ST13
RFXAP
ITPR3
SCAF11
FASTKD3
SBNO1
ZNF669
FOXJ1
LINC01023
GATSL3
FAM103A1
TTLL13
DNAJC9-AS1
DKFZp779M0652
ATP5S
SMG9
GGT7
LOXHD1
TCAP
ZNF787
CD22
GHITM
FOXO4

CDH15
VWA5B2
LOC101929683
HIC1
TMEM126A
LOC102724561
NTRK1
ADAMTS15
PHLDB3
GTF3C1
ENTPD2
CLCC1
ACTR3
UBQLN4
DYNC1I1
