# Supplementary material for: Bioinformatics Approach to Identifying Molecular Targets of Isoliquiritigenin Affecting Chronic Obstructive Pulmonary Disease: A Machine Learning Pharmacology Study
Source: Int J Mol Sci. 2025 Apr 21;26(8):3907. doi: 10.3390/ijms26083907 (PMC12027559; doi:10.3390/ijms26083907)
Supplement: Supplementary file 1 [file ijms-26-03907-s001.zip › tableS2.pdf]

Table S2 COPD target gene lists obtained from the database

Only GeneCards	Only CTD	GeneCards AND CTD
POT1	THY1	PTEN
DNAH5	COL3A1	BRCA1
CFTR-AS1	SDC1	BRCA2
LOC111674472	KRT15	ADAM33
SOD2-OT1	TNFAIP3	FLCN
MIRLET7C	BNIP3	KRAS
PGR-AS1	SRRM2	CDKN1B
MEN1	CTNNA1	IL17A
CYP4A22-AS1	CASP7	BRIP1
LOC107063610	TRADD	MUC5B
MLH3	STAR	HNF1A
CERNA3	LIF	CYP2C9
LEPQTL1	PMAIP1	LEP
DCTN4	DLL4	MBL2
MRE11	ATF2	NOS3
EXT2	MT3	TSC2
PHOX2B	TNFRSF11A	TSC1
SOS2	MAP3K14	IL4
AIP	LIFR	CCL2
HOXB13	EPB41L1	VEGFA
FANCB	KITL	MUC5AC
SHOC2	HNRNPAB	HFE
TMEM127	PER2	ERCC2
TERC	PDK2	ERCC1
MIR155HG	NDC80	C5AR1
CYP2A6	ADGRE5	PLA2G2D
MIR199A1	PSRC1	LTA
LINC02605	FOSB	ATM
COPDA1	SPON2	CDKN2A
LUCAT1	ANPEP	PTGS2
SAT2	TRAF1	NBN
DNAJC18	MYBPH	BARD1
LINC00824	LAD1	XPC
HHIP-AS1	SERPINB2	ERCC5
MIR195	MIR21A	STK11
BDNF-AS	FOXM1	MSH6
CYP4A22	CPT1B	CSF3
CYP4Z1	CD72	CCL5
MIR4713HG	GFOD1	NFE2L2
PIRC66	PLEKHM1	CDK4
LOC126805899	CD47	MET
LOC111674475	GRB10	RET
LOC111674477	TM7SF2	HRAS
LOC113633877	PIMREG	AXIN2
LOC113664106	PLXNB2	APC
TERF1	IL24	FANCA
BPI	DCBLD2	MLH1
MIR142	SETD1B	PMS2
CSMD1	CIITA	ERCC3

MSTN	REL	MSH2
SCNN1B	PIK3R1	LIG1
INVS	ABL2	POLD1
DNAI1	CCL8	WRN
OFD1	AQP1	CDKN1C
GAS8	TGFBI	ERCC4
DRC1	KLHL21	EXO1
DNAAF19	MRPL4	PMS1
HFE-AS1	IER2	CAV1
LOC111674463	PTHLH	NOS2
CHRNB4	MTA1	CCL11
MIR664A	ERH	ALB
PDCD1	MXD1	GATA2
TAFA2	OLR1	RNASE3
JAKMIP3	RHOBTB1	CYP1A1
LINC00928	TRAF3	SLPI
CYP2F1	IRF2BPL	TLR2
AZU1	REEP2	XRCC1
DDX1	SLC8A1	NPPB
CCNH	SPC25	CXCR2
LCN1	CLDN1	MTOR
MIR181D	CBFA2T3	PDGFRA
MIR218-1	MS4A1	SMAD4
MIR543	RFTN1	FBLN5
ASXL1	MFN1	HYDIN
CETP	SLC3A2	EZH2
MIR199A2	TH	ALK
OIP5-AS1	NFIL3	SDHB
TRD-GTC9-1	ENDOG	CXCR3
HTR1D	LENG8	EDN1
RB1CC1	INS1	SFTPC
SSTR3	PLCG1	NFKB1
AMT	MT1	ICAM1
ADH1C	BCL2L12	CYP2C8
PARP4	CASP6	HSPA1B
SLAMF1	MAD2L1	CYP19A1
CYP3A7	PDK4	IL18
ABCA6	CLDN11	SERPINE1
SELENOF	ACVR1B	CXCR1
ZNF350	FBXL6	SIRT1
TMEM169	SLC7A8	SOD1
TSPO2	PGAM1	ADIPOQ
ZNF230	TEAD4	PRTN3
IGF2-AS	SPTBN1	IL33
MIR190A	ETV4	RAF1
MIR203A	GNB1	RAD50
MIR128-1	TBXAS1	TOP2B
MIR24-2	CASP2	RASA2
MIR1-2	PLCB1	FNIP1
LINC03111	BTG2	TNFRSF1A
PWAR1	BBC3	SOD3

COPD	TPT1	DNTTIP2
SNHG5	ITGA6	SOD2
TINCR	KIF23	EFEMP2
LINC00229	PXN	NPPA
IGHE	CLCF1	ENG
MIR183	TNFAIP8	HIF1A
MIR1246	TRNP1	BMP6
SKIC2	E2F7	DSP
MCM9	HMMR	CAT
ZBTB24	SLC39A9	HTR2A
SLX1A	GOT1	CYP1A2
MIR208B	CDKN2B	CHRM3
CCR8	DDIAS	ATP2A2
CRLF2	UCP1	HSPA1L
MIR16-1	GDA	CYP2J2
BCYRN1	PFKFB3	GCLC
MIR16-2	TEK	MMP2
MIR4785	PHLDA1	CCR5
LNX1	SNRNP70	SFTPA1
MYPN	CFB	NOTCH1
HTR4	CAVIN2	MIF
MIR29B2	CORO1A	XRCC5
MIR1248	ITPR2	CBL
SALRNA3	PRDX3	SMARCA4
SALRNA2	KLK10	NF2
CDH15	MMP11	EPCAM
B3GAT1	PPP1R14A	SDHA
OPCML	PER1	MUTYH
SIGLEC9	BCAR3	MIR146A
IL36B	EIF4G1	BMPR2
MIR302D	GINS2	NOTCH3
MIR497	SRSF5	ABCB1
MIR218-2	NUDT1	IL2
MIR320E	KRT8	PPARG
MIR625	KLF16	FGF7
MIR203B	RUFY1	SLC6A4
MUC8	PF4	CSF2
MIR6501	DNM1L	GYPA
LOC110806262	IDO1	TP73
ADGRG6	IKBKG	OGG1
CCDC38	MBD1	ARNT2
NCR3	PRR5L	TOLLIP
CFDP1	PTPRU	PCNA
ARMC2	SLC2A9	IL5
SPATA9	UHRF1	CHEK2
LINC00310	JUNB	MAP2K1
HDAC8	PBK	PTPN11
F11	SNHG29	BUB1B
CYP4F2	RUVBL2	WT1
IL12RB2	NDUFA4L2	CYLD
NR2C2	TRAF3IP3	XPA

FBXO11	PTPRC	XRCC2
SERPINA7	FGR	FN1
TFAP2C	MAPK10	TBX4
CRYGD	HTRA2	MIR125A
UTS2	TLE3	CYP1B1
POC1B	HK2	CTLA4
LGALS13	RNF213	MCAM
MT-CO1	SLC35F5	KIT
MYNN	CREM	SDHAF2
NT5DC1	BCOR	SRP72
ZKSCAN5	CTSK	P2RX7
ROMO1	CABYR	CDKN1A
SIGLEC14	PYCR2	ATG7
APELA	HNRNPH1	SERPINA1
MIR204	CIT	RAD23B
MIR26A1	GALNT10	DNAAF4
MIR128-2	GALNT3	CD4
MIR130B	GSTA2	SERPINB1
MIR134	ARHGAP19	SP1
MIR181A1	TNRC18	BCL2
MIR133A2	ATXN7L3	CD40
MIR26A2	E2F1	DEFB1
MIR410	LAMB1	AQP5
MIR454	GCH1	PSMA4
MIR873	COX2	XDH
SPAAR	MAP3K8	CXCL1
MIR133A1	NFAT5	MIR21
MIR518B	NPR3	GDF15
SNORD24	EPS8L2	IL1RAP
MIR508	KLF13	CHRNA4
NNT-AS1	PLXDC2	IL4R
MIR105-1	BMP1	CASP3
MIR380	KIF14	SCNN1A
MIR766	TNFSF4	COL4A1
FIRRE	CENPA	COL4A2
MIR524	DUSP3	INPP5E
MIR765	EFNB1	BRAF
IL6-AS1	TUBA1A	MAP2K2
MIR124-1HG	TRMT10A	BMPR1A
MIR941-1	LGALS1	NRAS
MIR1260A	MCM5	PRKAR1A
MIR3202-1	MYLK	SOS1
VPS9D1-AS1	LPIN1	RUNX1
MIR4476	MST1R	DICER1
TTTY4	OIP5	IKZF1
MIR105-2	COL12A1	MITF
EIF3J-DT	BTBD3	NF1
MIR941-2	FAM53B	POLE
MIR941-4	MMP10	PPM1D
MIR4736	TNFAIP2	BAP1
MIR634	MCM2	CEBPA

SALRNA1	PERP	DKC1
LINC01414	AP1S1	EXT1
MIR1233-1	EPOR	FANCC
MIR4316	CCND2	FANCD2
MIR941-3	AR	KITLG
LINC01822	AKIRIN1	PAX5
MIR1233-2	CPNE2	PRF1
MIR941-5	FOXF1	VHL
MBL3P	IGSF6	CDC73
MIR5007	PLSCR4	DDB2
MIR6072	POU2AF1	ETV6
MIR8079	PSTPIP2	FANCL
LOC106728418	RHOF	FH
TRA-TGC7-1	AK2	GPC3
F9	EAR1	MAX
SERPINA4	PYCARD	POLH
SERPINA12	COL5A3	RIT1
CTRB2	RIN1	SMARCB1
SERPINA9	HERPUD1	SDHD
TMBIM4	BAD	FANCG
USP15	GAS1	GREM1
AEBP2	MCF2L	NSD1
FBXO38	MSLN	OAS1
INTS12	NTN1	PALB2
OR12D3	SLC22A1	CD70
PAPPA-AS1	PCF11	DDX41
CRMA	PHYHD1	NSUN2
LINC01940	PPTC7	NTHL1
MIA-RAB4B	PPP3CA	RAD51C
CYP2F2P	CD163L1	REST
DNMT3AP1	NDRG1	SDHC
GNG5P5	HMGA1	FANCM
KRT18P51	PMP22	LZTR1
RPL36AP26	TFPI	SBDS
LOC101928387	APCS	SUFU
MT-CO2	CHST15	CEP57
MIR184	COX5B	DIS3L2
MIR151A	FAM43A	FANCE
LINC01590	UGP2	FANCI
MIR1236	DENND3	ICOSLG
RPL31P11	INHBA	RECQL4
DNAH8	EPHA2	RRAS
VKORC1	MAT2A	SLC39A7
CDON	HMBS	SPRED1
GHSR	RSAD2	TINF2
MYLK2	PSTPIP1	FANCF
PDE6A	SSTR4	RAD51D
TAOK1	CMPK2	RHBDF2
CPA1	DLGAP5	ANKRD26
FBL	SPHK1	SLX4
LMX1B	CAPN3	SAMD9L

MAPKAPK5	IFT80	JUN
NDUFA10	MARK2	CXCL10
NR2E3	MYL2	TTR
SETDB1	SP110	ALOX5
SLC14A1	ITGA7	MIR34C
STAG3	MT2	CTNNB1
CNGB1	E2F3	IGF1
CRABP1	KDM6B	XRCC3
DEPDC5	TBXA2R	AKT1
DSTYK	AP2A1	LPL
KCNIP1	ARHGEF16	TF
NEUROD2	HGS	NOS1
RING1	IL20RB	CYP2E1
ATL1	LIPG	NAT2
CNTN3	NKD2	CLCA1
ELAVL2	SETD6	CHI3L1
HLA-DRB5	SLCO1B1	APEX1
KIF3A	TPM4	XBP1
NBAS	CTNNAL1	APOE
PABPC4	HIGD1A	SLC22A5
SPPL2A	CCRL2	SLC22A4
EIF3E	DLG4	TET2
IL17B	SART1	MIR133B
KANSL1	SLC29A1	CYP2D6
KIF3B	TAFA5	HDAC9
LLGL1	LAPTM5	NQO1
MORC2	HSD11B2	IL12A
MTMR3	NEFH	PVT1
NR2C1	GBP1	CCR4
RFX3	ARL6	CTSG
TRAF3IP1	KIAA0930	SELP
ANAPC2	USP22	PIM1
ARHGEF28	RASGRP1	ABCB11
ATF7	CLCN7	NR1H4
CERS6	FCHSD2	MAPK14
CPSF6	KDM4B	IL1A
EMSY	NLRP12	CCL3
FAM3C	NUSAP1	TIMP2
LEMD2	ZEB1	CS
RBBP5	MAP3K6	SMAD3
TASP1	BHLHE40	IRF1
TSHZ3	TNFAIP8L3	LCN2
BNC2	ATG2A	NFKBIA
COPS8	BBS2	GATA3
CRPPA	MIB2	BAX
DRAM2	MIEF1	PDE4D
PDZRN3	TNK2	TICRR
RFX6	STX3	MYC
SLC5A11	GAS6	TGFBR1
VTA1	PRKCB	GC
YEATS4	NR4A1	MMP1

ZNF184	CD40LG	ADRB1
CTU2	CTNND1	ALDH2
GTF2H4	IDI1	RIN3
RAB3IP	S100A11	PARP1
SF3A3	CDK5RAP2	GPX1
ANAPC5	SLC39A11	RSPH1
ASB13	SLC16A3	RSPH9
ATP6V1G2	WSCD2	RSPH3
BTBD1	PDGFB	IL6R
BTN1A1	EFNA1	CCR3
BTN3A2	ERF	DNAAF2
GOSR1	PPP1R13L	HGF
HLA-DOA	PPP1R16B	ITGAM
HLA-DQB2	BMP2	CAMP
INPP5F	NUPR1	NLRP3
OTUD7B	SCD1	PLAUR
SCGB2A1	ITGA5	FGF1
SLC10A7	CEP55	PHB1
SYNPO2L	COLGALT1	F2
TUT1	CPT1A	CYBB
UBAC2	EYA3	CD80
VPS52	INPP5A	EGF
CEACAM4	SLC20A1	ADM
CPA5	CDCA5	COL1A1
FKBPL	EFNB2	HMGB1
HORMAD1	APP	PON1
HOXC4	ABCC3	CD14
L3MBTL3	MYL12A	IL7R
MKRN2	SLC43A3	EPO
NUFIP1	ATP5F1A	FLT1
RANBP10	OCLN	FPR2
RTRAF	CEBPD	FTO
SHKBP1	EEF2K	TNKS
TM9SF4	NCAPD2	ARNT
TMCO5A	TNFRSF25	C4A
AFG1L	ANKRD16	HLA-C
ASCC2	PBX4	IGFBP6
BCL7C	CHST1	AKR1A1
DCHS2	NDUFS5	HTR1B
EAPP	HSPA2	MEST
GET1	OSMR	SCUBE2
INSM2	CTLA2B	TNIP1
MBOAT2	FAM167A	AHNAK
MTHFSD	PTP4A3	RERG
NUP42	S100A14	RNF150
PPP4R4	SOCS4	HCG27
SRFBP1	ACACB	ATF6
TESK2	ABLIM1	INS
TPRN	DBN1	H19
TRMO	EIF1	IGF2
C12orf43	MTUS1	GSTM3

CAPZA3	RFC4	NOD2
DNAJB8	VAT1	VCAM1
FAM168A	GAP43	F3
HLA-DOB	EVA1A	MIR126
TAPBPL	TIMM17A	UCP3
TEKT5	TAGLN	CBS
ZNF629	DDX3X	IRF3
CCDC7	ETV5	NR3C1
CFAP70	ACKR1	GPT
CHST9	C5AR2	CEACAM6
CTAGE1	MEFV	ABCA1
EFCAB5	STRN4	FBXO32
FBXL22	SYCE2	MDM2
LRRC37B	TMEM63B	RAD51
NOL4L	UPK1B	ERCC6
SEC61A2	ATG12	XRCC4
SGF29	RPS6KA1	RAD52
SNRNP48	SORBS1	ADORA2B
THAP4	TIFA	HADHA
TIMM21	DLD	CD86
TTC23	FCGR2B	GSTCD
AFTPH	EVPL	CYP11A1
DNAH12	PARPBP	CYP4B1
DYDC1	EIF4A1	IGF1R
DYDC2	PRDM1	CHEK1
FAM118B	KIF20A	MMP8
GYPE	BORA	MMP7
H2AC16	TMEM47	CTSS
H4C13	DDX5	IL17F
HOXC5	SLC25A44	S100A8
OTULINL	ID1	MIR223
RALGPS2	SEMA3C	TGFB2
SPPL2C	SLC7A7	STAT3
SUPT7L	CARD10	KCNK3
TMEM129	HR	KCNN4
TSBP1	PIK3R2	EIF2AK4
UBE2Q1	PLEK	BDKRB2
C1orf127	SFXN3	FAS
DNAJC27	HSF1	FGF2
FUT11	ANKRD1	SLC26A4
LRRC37A2	CHKA	MUC2
NUDT13	SMC4	DEFB4A
SLC25A51	TRIP13	MIR145
TMEM170A	MAP2	SERPINI1
YLPM1	AGBL2	TFEB
ZNF319	B4GALT7	BCR
ARL17A	ST8SIA1	PLK1
ASB14	CD109	CCNA2
C15orf40	IL4RA	POLB
DTHD1	PLCD1	RAD54L
MICU3	CHORDC1	LIG3

PGBD1	IFI27	MSH3
TIGD6	ZNF30	RAG1
TRIM40	MAP2K6	TERF2
USP54	KCND3	HUS1
ZNF546	MLPH	WRAP53
ACTMAP	NOTCH4	AHR
CYB5D2	PPID	TNNT2
METTL4	SEMA4D	IL15
RAMAC	ZNF563	COMT
SPHKAP	KCNH2	REN
TMEM51	GLS	IL3
TTC9B	CAMTA2	IL9
C6orf136	CEP83	LBP
EXD3	DIRAS2	FGB
FBXO46	TMEM214	TNS1
OR2B2	CTPS1	IL12B
PROX2	HIPK2	GCLM
PVRIG	ANKRD10	IL16
ZNF311	KCNK5	ANXA1
LY6G5B	RALGDS	PTGS1
C1orf162	SRGAP3	TRPV4
C5orf34	TBC1D2	MSR1
KCNJ18	CCL6	CX3CL1
KHDC1L	COTL1	PTX3
OR10AD1	SLC16A6	GHRL
ZNF391	TNFAIP6	IL15RA
ZSCAN9	DUSP5	CD27
C6orf47	MINK1	EGLN2
FNDC9	SNX18	OPA1
GPALPP1	SUMF2	RAPGEF3
OR5V1	UBE2V1	FUT8
SCGB1D4	DNAJC2	PLA2G6
WDR64	FAM107A	TNFRSF11B
AGAP5	GDI1	VIP
MEIKIN	LYAR	MYO1A
ZSCAN23	NEURL4	TFAM
C5orf52	RHBDF1	TREM1
CCDC26	SACS	IL22
KRBOX5	TMEM126B	NCF2
SMIM23	DGAT2	VEGFC
SPDYE3	MEIS1	PAPPA
TMEM267	VASN	NOTCH2
TNXA	GNAS	ACVRL1
RNF32-DT	AGRP	PLG
C1orf185	EMC8	EDNRA
C5orf47	FAM133B	BMPR1B
GOLGA6L4	SYT8	ABCC6
HLA-H	MAP2K3	DVL1
LINC00470	RHOC	FCGR2A
POM121L2	IFI44	SLC9A3
FAM167A-AS1	GJB3	STX1A

FBXL19-AS1	HINT1	SMAD9
GOLGA6L10	TNFSF15	SARS2
GOLGA6L9	TGFB3	CAV3
TNFSF12-TNFSF13	AOX1	CENPF
TRIM39-RPP21	G0S2	DNAH11
CARMN	ACAP1	CEACAM3
HCG22	PROCA1	RPGR
LINC02693	TMEM132A	CCNO
POLR1HASP	ZNF608	SPAG1
MAPT-AS1	FGD4	ABCC10
SCAND2P	RHOU	DNAL1
COL4A2-AS2	TREM2	SLC6A14
PSORS1C3	KRT5	NME8
GATA3-AS1	P4HA1	CLN6
PMS2P1	CLDN5	DNAI2
CARINH	ENC1	ZMYND10
CASC17	BACH1	CCDC40
CYP21A1P	SHC1	CFAP298
HCG25	SLC25A4	RSPH4A
MIR548B	CDC34	SLC26A9
SNORA48	ELOVL1	CCDC39
TET2-AS1	GTPBP2	CCDC65
TSPY26P	DDX27	CLCA4
CYP2G1P	REXO4	DHX34
HLA-F-AS1	RFNG	DNAAF1
HOXC-AS1	ADGRE1	DNAAF11
HSP90AB2P	CAR3	DNAAF3
LINC00322	FBXL19	ODAD2
MDC1-AS1	PPP1R42	TAMM41
MIR1825	CORO2A	DNAAF5
CD27-AS1	KRT4	DNAH1
CHRM3-AS2	SAT1	ODAD1
CTBP1-DT	DLX4	DNAAF6
DNAJC27-AS1	SDF4	HMOX1
ELN-AS1	SLC35G2	VDR
FGF10-AS1	STRADA	MGST3
GVQW3	SUN1	HYKK
H2BC20P	CORO1C	MGST1
LINC01370	PTPRG	CCND1
LINC01381	ZBTB10	AVP
OR2B8	EGR2	CD274
SNORD117	TACC3	TLR6
SUZ12P1	ABI3BP	TNFSF13B
TGFB2-AS1	ASAP1	CXCL11
LINC00649	CENPM	IL27
MIR4708	KRT19	MIRLET7D
PSMB8-AS1	CDH16	CASR
SNORD16	NRG2	ATP2A1
SPRY4-AS1	RNF149	HSD17B4
TBX3-AS1	SCEL	EZR
ASTN2-AS1	ANLN	CBR1

CALML3-AS1	CACNA1C	MATR3
HCG18	TUBA1B	MX1
HLA-J	CRH	STAT6
IPO9-AS1	CH25H	GZMB
LINC01505	KIF18B	NAMPT
LNC-LBCS	PRKD1	CCR2
MIR1227	PTBP1	MIR320A
MIR4733HG	SEMA3A	ERBB3
SMIM2-AS1	SPAG9	WNT7A
USP3-AS1	TBX21	LOX
ARMC2-AS1	RGCC	RARG
CPEB1-AS1	SOX2	EYA1
HCG4	ADAP1	ANXA11
LINC01254	LDB1	BTC
LINC02038	LPCAT4	GPNMB
LYPLAL1-AS1	SLC25A32	ATP2C2
MIR3945HG	TMC4	ATG4B
MIR4693	TP53I11	BMAL1
MPDU1-AS1	DHCR24	SUMF1
PPIEL	FKBP4	CAMK1D
PPP1R3B-DT	PHLDA2	DNER
CT66	RACGAP1	SIGLEC1
DOCK9-DT	ATOSA	ASRGL1
HLA-DRB6	ATP5F1D	EHF
LINC01470	CD276	NTM
LINC02569	PRKAR2A	ZFP36L1
LINC02869	PTPRE	ACYP1
LINC02991	SLC46A3	APIP
SMAD3-AS1	UBE2D3	CCHCR1
COX10-DT	ZFHX3	ORMDL3
HCG4B	NHLRC1	PRDM15
HLA-DQB1-AS1	POLR1B	SCLT1
KU-MEL-3	EFEMP1	FPR3
LINC00501	MEF2C	KAZN
LINC00578	CDC20	ORAI3
LINC00886	ABCD2	PELI2
LINC01091	KIF4A	PSORS1C1
LINC01162	OAS3	PSORS1C2
LINC01804	TBK1	TMEM254
LINC01844	AMMECR1	MIR99AHG
LINC01876	CEP78	LINC00299
LINC02026	CHFR	IL10RA
LINC02288	FOXK1	S100A9
LINC02354	GNPTAB	FGFR1
MIR548Q	PKN1	CASP8
NIPAL4-DT	RAB25	SCARB1
NRIP3-DT	ETS2	SLC2A4
RPLP0P2	CDCP1	APOB
RPSAP52	CSRP3	CYP2C19
SNORD124	HIBADH	PTH
TIPARP-AS1	PDPN	GSTO1

VESTAR	PROS1	DUOX1
ZNF204P	MIR434	SHBG
ZSWIM8-AS1	ZNF845	GPR182
B4GALT4-AS1	ARHGAP21	ANXA5
ENSG00000202479	CABIN1	TLR9
GRPEL2-AS1	GRAMD1A	EGR1
HLA-L	NAP1L4	SIRT6
HSP90B3P	PRR7	RTEL1
KLHL7-DT	CCNF	LACTB
LINC00402	CDH5	LMNB1
LINC00670	MIRLET7I	GDF11
LINC01093	SLFN4	CXCR4
LINC01239	USP2	TNFRSF10A
LINC01322	ATP6V1B2	IFNGR2
LINC01748	BMF	MIR34A
LINC01975	EFHD2	AGT
LOC100287290	MEF2A	PTMA
LOC100287329	TENT5A	TLR3
LOC730183	CTSB	LEPR
PPT2-EGFL8	ENO3	MTHFR
SAMD12-AS1	GNAI2	WWOX
SAPCD1-AS1	RBM47	CYBA
TNS1-AS1	WDR1	CHUK
TRIM31-AS1	BAG5	PIK3CG
VN1R10P	HOOK1	KNG1
ZNF652-AS1	PORCN	THBD
ADAMTS19-AS1	RAC3	STAT1
BMS1P4-AGAP5	VTCN1	IFNAR2
CDRT15P1	ARF1	IGFBP3
COX11P1	SREBF2	SPP1
EHBP1-AS1	C1S	CST3
GLUD1P3	FBXO30	LTB4R
GOLGA6L5P	ITGA4	CCL20
GUSBP5	MAP3K1	CXCL5
HLA-DRB9	SAA3	SEC24C
HLA-K	VASP	MAPK1
HLA-V	VSNL1	ADA
IER3-AS1	MFSD9	SRC
LINC00933	ZBTB25	C3
LINC01153	KIF3C	HSPA5
LINC01509	CSRP1	MUC1
LINC01581	KPNA2	HP
LINC01752	FILIP1L	TXN
LINC01807	PSMB6	CXCL2
LINC01933	FAM83A	ESR1
LINC02020	CENPE	SLC6A3
LINC02576	HDC	DRD2
LOC100506444	MAOA	CYCS
MIR4432HG	ATG16L2	FASLG
MIR4734	EHD4	KEAP1
MIR5680	IFT122	KL

MIR762HG	IPCEF1	MTR
RPL17P39	LZTFL1	RIGI
RPL21P44	MAZ	CCN2
RUNX2-AS1	MRPS22	MTRR
RUNX3-AS1	NFE2L3	BHMT
SH3PXD2A-AS1	OSBPL5	AHRR
SYNPO2L-AS1	PABPC1L	CCL18
TSBP1-AS1	PARVB	MIR206
TSHZ3-AS1	PHYH	ACD
YBX3P1	RHEBL1	THSD4
ZNF747-DT	SH3D21	HSPB1
ABHD15-AS1	SNCA	SERPINC1
BMS1P4	TIMM44	PTGER2
C5orf34-AS1	COL5A2	TTN
FILNC1	SLC6A9	NEU1
H2AZ1-DT	CAMKK2	MIR17
HLA-W	COX4I1	ERBB2
LASTR	DEPDC1B	FOXO1
LINC02005	GRIA1	GSK3B
LINC02017	PKMYT1	TFRC
LINC02863	PLAC8	CYP17A1
LINC02934	PRICKLE1	HDAC1
MICD	PRKAB1	TYMS
MIR1289-2	GSTM2	LRP5
MRPS35-DT	CIZ1	MGMT
SNHG26	IMPDH1	CASP9
TOB2P1	KSR1	BRD4
AZGP1P1	MIR222	IGF2R
CAMK2G-AS1	PCBP3	ITGAL
CEP72-DT	SH3RF3	SLC19A1
CHD1-DT	ZBTB7A	DRD4
CPMER	CCNG1	PTGER4
EEF1A1P9	ARL4D	STAT4
EMSY-DT	BBOX1	COX5A
ENSG00000249209	CUX1	CX3CR1
ENSG00000268584	DBF4	TFF3
ETF1P1	NCAPG2	TRIM63
GSTCD-AS1	GCOM1	CXCL9
HLA-U	KLHL17	GSTO2
IGFBP-AS1	EVA1B	CXCL12
LINC01267	FNTA	PIK3CA
LINC01603	HCLS1	FOS
LINC01810	INPP4A	MMP14
LINC01818	RNF144A	DPP4
LINC01833	TMC7	CFH
LINC01937	BCL3	ALOX15
LINC02082	ETS1	CD81
LINC02284	FCER1G	COL18A1
LINC02463	RBM39	CYP27B1
LINC02533	RGS1	GHR
LINC02540	ACACA	IL5RA

LINC02571	WDR97	AKR1C3
LINC02676	KIF2C	BAK1
LINC02677	DKK3	LGALS3
LINC02757	GRK5	TSG101
LINC02778	H2AC6	ADIPOR1
LINC02919	IHH	GLRX
LINC02924	SOAT1	IGFBP2
LINC02938	SPC24	SELL
LINC02941	SLC7A5	BAMBI
LNPPS	ADGRD1	GZMA
LOC101929577	BLOC1S2	IGFBP5
LOC102723604	IBTK	GPX3
LOC102724684	CALM1	DIO1
LOC102724877	DAB2	IL11
LOC105374901	CEMIP2	MBD2
LOC105376805	CLDN7	RGS5
LOC124900452	STK17B	SAA1
LOC124904611	TCF7	IL23A
LOC158434	ATP5MJ	CCL1
LOC439933	STK33	RGS17
MICA-AS1	TEX11	RAB15
MICE	ZFP41	MIR29B1
MIR4464	ZNF410	MIR1-1
MIR6731	CCNE2	MIR374A
OXCT2P1	DGAT1	PRKCA
PABPC4-AS1	KYNU	B2M
PDE3A-AS1	NFKBIB	CD36
PDGFDDN	ARPC4	GGT1
RPL7P6	CD247	RHOA
RPS20P4	GAPVD1	PRDX6
SENP3-EIF4A1	LIPH	VTN
SMG1P5	MAN2C1	MUC4
SNORD32B	MLLT6	MUC6
SNORD65B	NSMF	MIR23A
TSPAN14-AS1	RETNLA	MIR98
UQCRHP1	SLC29A2	MIR146B
DIRC3-AS1	TRMT6	INSR
DUSP8P5	AMIGO2	CDK5
ENSG00000225335	ETFB	SLC2A1
ENSG00000230730	NFIC	AURKA
ENSG00000231953	EGLN3	CSF1R
ENSG00000234389	GBP2	JAK3
ENSG00000236304	ALDH9A1	IFNGR1
ENSG00000242795	ANTXR2	LDLR
ENSG00000243089	DAG1	CTH
ENSG00000248647	MAPK11	GSR
ENSG00000250659	METRNL	PGR
ENSG00000250740	ADRA2B	FKBP5
ENSG00000251259	ARPC5L	RAC1
ENSG00000253406	CLUH	ABCC2
ENSG00000254649	CNTNAP1	CALCR

ENSG00000254664	FCMR	CDC25A
ENSG00000254810	FHOD1	CDC25C
ENSG00000255126	MAP4K2	CUL3
ENSG00000255237	MOCS1	DHFR
ENSG00000255250	MYL12B	ESR2
ENSG00000255446	NADK	HMGCR
ENSG00000256433	SERTAD4	IRS1
ENSG00000256462	SPATA20	RXRA
ENSG00000257058	METTL17	TYR
ENSG00000260271	LRRC74B	APAF1
ENSG00000260597	XLR3B	BIRC3
ENSG00000260608	ADCY7	CASP10
ENSG00000260911	DHRS4	CDC25B
ENSG00000261474	DLL1	CYP24A1
ENSG00000261783	DSC2	FOXP3
ENSG00000266469	GPC1	GLP1R
ENSG00000269843	PMVK	GPX4
ENSG00000270175	WSB1	LCAT
ENSG00000272140	CFD	LRP6
ENSG00000273049	TNFRSF9	MASP1
ENSG00000282278	ADAMTS15	NCOA3
EPC1-AS1	AP1M2	PIN1
HCG17	ELOB	PLA2G2A
LINC00709	PWWP3A	ROS1
LINC01830	SBNO2	ABCC4
LINC01997	TXNIP	ALAD
LINC02305	CENPU	APOA2
LINC02386	GUCY1B1	BCL6
LINC02459	MKI67	CCND3
LINC02468	SLC13A3	CDK7
LINC03017	TRA2B	CHRNA1
LOC100289230	PKHD1	CTSH
LOC101929563	ARHGAP33	CYP7B1
LOC105370887	CLBA1	EPHX2
LOC124900382	GBP7	FBXW7
LOC124901882	IFT22	LIPC
MIR3171HG	MISP	RXRB
MIR4282	ASPM	TACSTD2
MIR4456	DUSP8	TGM1
OR2E1P	OXCT1	TP63
PPP1CB-DT	ADAM15	ABCA7
RNU1-27P	COL17A1	AKR1C4
RNU6-588P	EGFL6	AMACR
RNU6-653P	FUT4	DRD1
RNU7-55P	SKA3	FUT2
RPL29P12	TTC9	HSD3B2
RPL37P19	NFATC1	ALOX12
RPL3P2	UCHL1	CGA
RPL7P1	DTL	HSPB8
RPS27P25	NEAT1	MYO5A
RUSF1-DT	MARCKSL1	OCA2

STAG3L5P	RANBP1	RNASEL
ZNF602P	SYNE1	ABCA5
ZNF603P	ZYX	AIMP2
BTF3P7	IFIT3	ARHGDIB
COPS5P1	PFKP	FOXC1
CSPG4P10	AATK	FZD7
ELOCP2	ATP5MC3	GGH
ENSG00000199332	CCDC68	HSD3B1
ENSG00000202533	CCNL2	IGFALS
ENSG00000205537	MRPL17	IGFBP1
ENSG00000226756	SEC14L1	MBD4
ENSG00000226842	SLC16A9	PAK6
ENSG00000228302	SYTL4	UGT1A4
ENSG00000229990	TEX264	APOA4
ENSG00000230694	ALDH3A2	LITAF
ENSG00000233754	CKAP4	LMO2
ENSG00000234793	CLDN18	OPRD1
ENSG00000253252	INTS14	TFF1
ENSG00000253519	MIR30D	GPX2
ENSG00000254497	OXTR	GSTZ1
ENSG00000258344	PTPRK	HIF1AN
ENSG00000259605	RBL1	HSD17B1
ENSG00000259713	RRM1	HSD17B2
ENSG00000259767	SDK1	IL13RA1
ENSG00000260077	SLC11A2	MPDU1
ENSG00000260267	SYS1	MTHFD2
ENSG00000260586	ZNF714	MYBL2
ENSG00000260773	ACP5	SLC4A2
ENSG00000261762	ALDH1A3	ALDH1L1
ENSG00000262766	CKS2	ATP1B2
ENSG00000263603	CALB2	BGLAP
ENSG00000265554	HILPDA	CBR3
ENSG00000267114	AHCTF1	GSTA4
ENSG00000269514	CLN3	RGS6
ENSG00000271716	DNAJB5	SOAT2
ENSG00000272050	GALNT15	TEP1
ENSG00000272501	NUCB1	CXCR6
ENSG00000272540	CAPN1	LMOD1
ENSG00000283782	SLC38A2	SEC14L2
FAT1P1	TIPARP	SLC23A2
H2BC16P	ANKRD37	SLC30A1
HCG9P5	CAMK4	TTF2
LINC01799	GART	APOM
LOC105375423	LIPA	GPRC5A
LOC124901221	RYR1	NFKBIE
LOC124903317	DYNLT2B	NUBP2
MIR6819	IFITM6	PECR
MIR6833	MIR30E	IL13RA2
NAP1L1P1	PCNX3	NINJ1
PAIP1P1	SHFL	SEPTIN2
RN7SL49P	AGO4	SLC16A4

RNU4-28P	AKNA	SLC23A1
RNU6-1208P	ARHGAP23	SLC30A4
RNU6-1216P	BEX1	TP53I3
RNU6-195P	CASP12	NUBP1
RNU6-920P	DMKN	SRA1
RPL19P2	FGF13	ZFPM1
RPL21P108	REC8	CSTF1
RPL29P1	PCK1	SELENOP
RPS13P5	COL7A1	TCEAL1
RSL24D1P6	EPB41L3	PCTP
TXNP1	FDXR	DHDH
ADIPINT	KIF11	KRT23
AK3P5	LMCD1	SPAG16
BCLAF1P1	UQCRC1	SLC39A2
BNIP3P1	ACO1	RMDN2
CAND1.11	DYNLT3	MREG
DNAJC8P1	FBXO3	MIR186
EEF1A1P19	MYO7A	MIR24-1
ENSG00000201207	PAN2	BID
ENSG00000205041	PRICKLE2	VAPA
ENSG00000226169	RHOJ	CTSD
ENSG00000226526	VPS13D	HSP90AA1
ENSG00000226816	ZNF543	HSPD1
ENSG00000228737	RFC3	IL1R1
ENSG00000231421	E2F8	PARK7
ENSG00000234913	NYNRIN	SCNN1G
ENSG00000235445	PELI3	TGM2
ENSG00000236772	VMA21	VCL
ENSG00000238221	HSD11B1	LTF
ENSG00000244757	HHEX	RECK
ENSG00000247934	ACTR2	CKMT1B
ENSG00000248245	CBX6	MIR155
ENSG00000248373	CCNB2	MIRLET7B
ENSG00000251775	CDK5RAP3	HLA-DPB1
ENSG00000253194	FABP1	HHIP
ENSG00000254094	H1-10	BMAL2
ENSG00000255021	MIR22HG	TUG1
ENSG00000255108	MYO18A	PDE3B
ENSG00000255118	RBM15	CYP2R1
ENSG00000255508	UNKL	FAM13A
ENSG00000255558	ABCG2	CYP46A1
ENSG00000255910	ORM1	TNF
ENSG00000256364	ANP32A	MMP12
ENSG00000259007	ASPH	EPHX1
ENSG00000259805	MTSS1	CXCL8
ENSG00000260304	ATG9B	ELANE
ENSG00000260579	BTG1	MEG3
ENSG00000261257	CD177	FENDRR
ENSG00000261487	MS4A6D	LINC-PINT
ENSG00000265443	PGAP6	TNFRSF1B
ENSG00000268434	PHF24	KRT18

ENSG00000268797	THAP3	VWF
ENSG00000269148	ZNF296	FGF10
ENSG00000269256	B3GALNT1	PRDX1
ENSG00000272236	BANP	GSDMA
ENSG00000274578	BCL9L	FEN1
ENSG00000281883	CD164	MCM3
ENSG00000285713	CDKAL1	DAXX
ENSG00000285783	ESAM	MDC1
FGFR3P1	EXOC3	RPS17
HMGN2P11	GEMIN4	MSH5
LINC02398	HBA-A1	TCOF1
LINC02549	MAPK8IP3	RNF4
LOC100130207	SELENOS	RAD51B
LOC105369591	SH3BP4	STN1
LOC124900426	SNX29	NSMCE2
MMP24-AS1-EDEM2	SNX8	ERCC6L2
MTCO3P1	ST3GAL3	H3C12
NOP56P1	ELF3	NGF
RN7SL316P	FZD2	NTRK1
RNA5SP96	HNMT	MAPK8
RNU6-229P	PPIF	PDE7A
RNU6-234P	ST3GAL5	CP
RNU6-351P	CFL1	CD8A
RNU6-758P	XIAP	A2M
RNU6-823P	PRKAG2	CANX
RNU6-850P	RAB31	TGFBR2
RNU6-929P	SLC39A10	RELB
RNU7-124P	SLC9A1	FGF23
RNY1P5	ATXN2L	CALCA
RPL10P3	CYP2A4	NNMT
RPL14P4	EPHB4	PPARA
RPL30P3	HECW2	TIMP3
RPS4XP9	ITPKB	RAC2
SCML2P2	KRT80	PDHA1
SERTAD3-AS1	PTRH2	MIR27A
SPON2-AS1	SLC25A24	CHIT1
TMX2P1	PEG10	AREG
TRD-GTC5-1	CSNK1G2	SNORD15A
UBA52P7	GPR135	TBX1
WDR45BP1	SLCO2B1	HLA-DPA1
WDR82P2	TSPAN11	RREB1
YWHAQP6	WIZ	MCIDAS
ATF1P1	CACYBP	GFI1
C1DP3	CAPN15	PLA2G7
CFL1P2	CKS1B	CHRM2
DNM1P51	DUSP2	PPARGC1A
ENSG00000093100	DYNC1H1	CYP3A5
ENSG00000220256	NABP1	IFNB1
ENSG00000227602	ATP6V0B	PIGR
ENSG00000228226	OSBPL8	MUC16
ENSG00000252136	RTN4R	IL32

ENSG00000255872	UBE2L3	MIR191
ENSG00000259555	YTHDC1	MIR29A
ENSG00000259683	MECP2	MIR221
ENSG00000264825	NCAPG	TNNI3
ENSG00000268069	TMT1A	COL6A5
ENSG00000271581	EPS8L1	GJA1
ENSG00000271787	DDC	DNM2
ENSG00000272788	ADAMTSL4	PSMB8
ENSG00000273046	ANKH	DES
ENSG00000273090	C1QTNF6	ITGB6
ENSG00000273368	EXOSC5	TAP1
ENSG00000273387	INTS8	MYH9
ENSG00000274737	LMF2	TARDBP
ENSG00000275263	LRRFIP1	PSMA6
ENSG00000277971	LSM4	SFN
ENSG00000282951	LY9	TAP2
ENSG00000286311	LYPD6B	ANXA6
ENSG00000286898	MIDEAS	BAG3
ENSG00000287497	MXD3	HLA-DQA1
ENSG00000287676	PDLIM7	PSMC4
ENSG00000293569	PI4K2B	CLIC1
GUSBP8	PKN3	PSMD3
HP09053	PLEKHG5	DMBT1
LOC101928272	SSRP1	HLA-E
MIR6074	TMEM259	SNRPA
NCOA4P1	ZNF185	AIF1
POLR2LP1	MAPT	CALML3
RN7SKP167	CCDC89	RPLP2
RN7SL354P	CRELD2	RNF5
RN7SL551P	MYH10	CTSC
RNU1-146P	RPL6	ADRA1D
RNU5E-7P	CSGALNACT1	EDEM1
RNU6-1244P	CYSTM1	HLA-B
RNU6-1339P	IGHM	HDAC6
RNU6-247P	LYVE1	EP300
RNU6-401P	NAGLU	MERTK
RNU6-502P	NUDC	WNT5A
RNU6-560P	PNO1	CREB1
RNU7-196P	CPS1	FGA
RPL29P17	FOXA1	ITGB2
RPL36AP20	MAT1A	PTPN6
RPL6P18	ACOXL	SQSTM1
RPS29P13	ASPRV1	RORA
RPS4XP18	CAPS2	ATF4
RPS6P12	CDH24	CD28
RPSAP2	INPP5K	FOXO3
USP8P1	POM121	MYOD1
ZDHHC20P2	PRR12	CHRM1
ZNF619P1	TEDC1	CD163
lnc-C9orf92-4	TMEM139	CCR1
ATP5MC1P8	KIR2DL3	PDE4B

ATP7BP1	IER5	FABP4
C1DP2	PKIA	PPBP
ENSG00000207173	RGS12	CA3
ENSG00000224431	RHBG	PTAFR
ENSG00000226329	SUSD2	OMP
ENSG00000229836	TNFRSF14	MIR132
ENSG00000230521	TRIM14	MIR211
ENSG00000236890	TSPAN7	MIR148A
ENSG00000237669	UQCRQ	MIR483
ENSG00000260082	EIF2S2	MIR193A
ENSG00000261349	GPD2	TGFBR3
ENSG00000276384	TG	ABCB4
ENSG00000286248	HS3ST2	TMPRSS2
ENSG00000287054	ANXA8	CDIPT
ENSG00000287982	AUTS2	FGFR2
ENSG00000288853	HIVEP3	PIK3CD
ENSG00000289311	SLC44A2	APOA1
ENSG00000289406	ZCCHC24	MMP13
ENSG00000289644	EVI2B	GATA4
ENSG00000290928	FAM193B	TNFSF11
H3P40	KLHDC4	CYP3A4
HMGN1P19	STARD9	MAP3K5
HSALNG0141674	TUBD1	CLU
HSALNG0142110	SBK3	NEDD4
JKAMPP1	SERHL	CUL1
LINC03028	MAFF	CYP2B6
LOC101928004	ASF1B	AOC3
LOC124901105	KIF15	PPIA
LOC124903544	GLUL	TGIF1
LOC124909486	QSOX1	TNFSF10
LOC126859646	CCL12	CD9
MTCO3P40	CHTF18	CDH13
MYL6P5	COL4A6	SELE
NONHSAG009182.2	HDAC11	WNT2
RN7SL222P	NUP50	ATG16L1
RNA5SP215	PHLDB1	CCR6
RNA5SP63	SERINC3	CUL4A
RNA5SP87	SKI	CYSLTR1
RNF6P1	LIMA1	FCGR1A
RNU4-69P	SLC7A2	HBA1
RNU6-214P	TUBB2B	POSTN
RNU6-276P	HMGB2	SELPLG
RNU6-718P	BCL2L15	VEGFB
RNU6ATAC28P	BFAR	RETN
RPL22P18	DSG3	ADAM8
RPL23AP44	LY6G6C	DIO2
RPS6P23	MAMLD1	CCN1
SUMO2P2	PPIL2	NUCB2
TUBAP15	TMEM79	CCL13
lnc-ACTRT3-5	VSIG4	IL36A
lnc-ASB13-5	FADD	CXCL14

lnc-ATP13A4-4	APOLD1	NUF2
lnc-CAMKK2-6	CPN1	MARCO
lnc-CNTLN-6	PAG1	BPIFB1
lnc-COL8A1-3	S1PR2	MALAT1
lnc-GLIS3-2	SORBS3	MIR143
lnc-HASPIN-2	TUBB4A	MIR106B
lnc-HFM1-4	MAP4K4	MIR10A
lnc-HLA-C-2	NR5A2	MIR196B
lnc-HOXC4-2	CCNB1	MIR20A
lnc-IRF1-1	FOLR1	MIR210
lnc-LEKR1-5	LCE1C	MIR574
lnc-MLPH-5	LCE1D	MIR99B
lnc-NDST2-5	MGL2	MIRLET7E
lnc-PLD1-2	MIR188	MIR377
lnc-RTRAF-2	PLK2	RTEL1-TNFRSF6B
lnc-SHB-1	SELENBP1	RARB
lnc-SLC25A51-5	ARHGAP45	BCL2L1
lnc-SLC25A51-8	ATP5PB	CHGA
lnc-SYF2-6	CLK4	PGF
lnc-SYF2-7	CLMP	ITGB8
lnc-TIAL1-1	LAT2	CASC15
AKAP8P1	PAPOLA	GLCCI1
BCAP31P1	RFC1	PDGFRB
ENSG00000215380	SYT12	IL2RA
ENSG00000255018	GIGYF1	MECOM
ENSG00000255585	NDUFAF3	HAVCR2
ENSG00000271547	NTMT1	HLA-A
ENSG00000289191	SMIM7	CHRNB1
ENSG00000292277	ABTB2	ITGA1
EXOC5P1	ADGRL2	HLA-DQB1
HLA-DQA1-AS1	EIF5A	MMP15
HSALNG0000794	EXOSC2	IL18R1
HSALNG0116321	GDI2	IL1RL1
LOC107986012	TFCP2L1	MFAP2
LOC124900868	TNFSF9	MICB
LOC124901481	C2CD5	CDC123
LOC124905027	ATP1A1	LRMDA
LOC124907776	RPS6	PLAGL2
NME2P3	THRB	ASB1
RNA5SP94	CLK1	ZKSCAN3
RNU7-159P	FGD2	RAB4B
RPL12P46	GGA3	JCAD
lnc-AKR7A2-3	KCNQ1OT1	HNF1A-AS1
lnc-ANKRD27-10	MRE11A	GRIN2B
lnc-BID-1	NUP62CL	PRKACA
lnc-CASKIN2-1	PMF1	PRKCD
lnc-CCDC38-2	PRAM1	AGTR1
lnc-CCDC7-11	RGL2	NT5E
lnc-CORO6-4	TMEM161A	AXL
lnc-CYP2A6-4	UBALD1	EIF2AK3
lnc-DYDC1-12	ZDHHC5	FZD4

lnc-ENPP4-4	JAG2	PLAT
lnc-FAM208B-7	N4BP2L1	PRKN
lnc-FILIP1L-6	SLC25A29	USP7
lnc-HIST1H2BN-2	PLK4	ACHE
lnc-IGF2BP3-1	CAPG	CD79A
lnc-INSM2-5	FOXP1	CHAT
lnc-LY86-4	SLC39A14	KAT2B
lnc-LYPD6-10	CCN4	MFN2
lnc-MOAP1-2	CGN	NRG1
lnc-NAXD-2	MPDZ	OPRM1
lnc-NNT-3	NDUFA4	PRKCZ
lnc-NRM-1	PIK3R5	HDAC5
lnc-NUMBL-1	PTGFR	MALT1
lnc-PABPC4-1	RPS6KB1	PINK1
lnc-PPP1R18-1	SHANK3	TLR5
lnc-RPS21-4	SLC12A4	WNT4
lnc-RUNX3-3	TMTC2	ACADS
lnc-SERP2-5	GLDC	BECN1
lnc-SIGLEC1-1	FAM53C	C5
lnc-SOX15-4	MAEA	EIF4EBP1
lnc-TFDP2-12	PGBD5	HRH1
lnc-TFDP2-13	PIGC	APOH
lnc-USP35-19	SH2B2	C3AR1
lnc-YEATS4-5	C19ORF48P	GALC
lnc-ZCCHC10-2	GFAP	KLF5
lnc-ZFP36L1-9	OGDH	RBP4
piR-38344-006	CAPN5	ADCY2
ENSG00000219201	CCDC86	AHSG
ENSG00000237550	CPLX2	APLNR
ENSG00000251473	MEX3B	DUOX2
ENSG00000254755	RASA3	E2F4
ENSG00000269069	SAFB	FOXG1
ENSG00000278733	ECE1	GRIK5
ENSG00000279108	NCOR2	IL23R
ENSG00000285619	SDF2L1	NTF3
ENSG00000288473	TFPI2	SNAI1
ENSG00000288813	TP53BP2	SOCS3
ENSG00000291129	ACADM	SOX5
ENSG00000291336	ACOD1	ST6GAL1
ENSG00000291338	ADAMTS14	ULK1
ENSG00000295324	GPR4	BNIP3L
ENSG00000296026	GTF2F2	MDK
ENSG00000296790	HIC2	NLRP1
ENSG00000298170	HOXA3	NPC2
ENSG00000298396	MT1E	NR1I2
ENSG00000298672	MYL3	PDCD1LG2
ENSG00000299012	NOVA2	RIPK3
ENSG00000299042	SYTL3	RORC
ENSG00000301589	TIMM50	SLC22A2
ENSG00000303969	TMEM41B	TNFRSF8
ENSG00000303982	TMPRSS4	UBC

ENSG00000305534	ZC3H7A	ITIH4
ENSG00000305770	ATP6V0A1	NFATC3
ENSG00000306007	CD22	PLA2G10
ENSG00000308496	DSCC1	CD58
ENSG00000308556	NOP2	CLDN4
ENSG00000310246	NUP93	KMT5B
GTF3AP1	POLG	MYF5
HSALNG0001756-001	SAA2	RAPGEF4
HSALNG0001938	SMAD5	BAIAP2
HSALNG0005167	UBE3A	CD83
HSALNG0006025	ADGB	CELSR1
HSALNG0006986	RIIAD1	EFNA2
HSALNG0013887	RTL8B	FFAR2
HSALNG0013889	UPK2	HCRT
HSALNG0014115	XIRP1	KLRD1
HSALNG0022208	ACAD11	MAP1LC3A
HSALNG0024737	DCAKD	MCM3AP
HSALNG0028087	ISG20	PIM3
HSALNG0028791	LIPE	PTGER1
HSALNG0030576-002	PRR11	CAP1
HSALNG0030876	PTPRS	IL36G
HSALNG0030878	TMEM37	MRC1
HSALNG0031687	POR	MYOG
HSALNG0031688	AMMECR1L	NBR1
HSALNG0032705	BMP3	KLF15
HSALNG0034443	CASKIN2	CCL26
HSALNG0037590-004	E2F6	CXCL16
HSALNG0044839	EDC3	DBP
HSALNG0044846	MED16	KDELR2
HSALNG0045244	MIR27B	NLRX1
HSALNG0045724	PRMT8	WFDC2
HSALNG0045755	PSMG4	ABHD2
HSALNG0045781	TEDC2	IL1F10
HSALNG0045902	WDR90	RAB32
HSALNG0046123	CRY1	SFRP5
HSALNG0046128	RPS27L	EFS
HSALNG0046567	TJP2	FUNDC1
HSALNG0046734	SEMA4F	LHX9
HSALNG0047910	UBE2C	FBXL16
HSALNG0047911	AMOTL2	GULP1
HSALNG0048776	CSPG4	MAPK15
HSALNG0048920	G2E3	HHLA2
HSALNG0048923	PHF19	CYP2A7
HSALNG0049180	RPL21	FAIM2
HSALNG0049181	SHISA2	TRIM16
HSALNG0049193	ZC3H12A	CDKN2B-AS1
HSALNG0049213	HMGCS2	MIR212
HSALNG0049243	ZFP966	MIR339
HSALNG0049249	DST	KIR2DS1
HSALNG0049408	JUP	MIR129-2
HSALNG0050329	MIR1224	MIR197

HSALNG0052607	MIR374B	MIR19A
HSALNG0053445	PPP1R27	MIR335
HSALNG0053897	BRD7	MIR582
HSALNG0055754	CLEC3B	SNHG8
HSALNG0055755	EFCAB2	MIR423
HSALNG0063305-001	GRM2	MIR361
HSALNG0075370	ITFG2	MIR1307
HSALNG0079213	KHNYN	MIR642A
HSALNG0081057	KNOP1	JAG1
HSALNG0082040	MRPS31	ATP7B
HSALNG0084599	RAB24	AFP
HSALNG0087989	RBM10	MAN1B1
HSALNG0087993	SLC25A28	OTC
HSALNG0091326	SYDE1	FAH
HSALNG0100587	TRIM11	GPT2
HSALNG0100588	UBAC1	SERPINA6
HSALNG0105091	ZFP69	SERPING1
HSALNG0111817	PHB2	ATG5
HSALNG0113537	TK1	SPINT2
HSALNG0115661	BIK	ATP8B1
HSALNG0117864	EBP	PNPLA3
HSALNG0117867	CYP27A1	ASGR1
HSALNG0126605	TMC5	CTRB1
HSALNG0126606	FLOT2	SERPINA10
HSALNG0126608	KLF7	MBOAT7
HSALNG0128259	MSI2	HSD17B13
HSALNG0129557	SH3BGRL	SERPINA11
HSALNG0129576	ST8SIA4	PPP1R12C
HSALNG0131531	BTF3L4	TM6SF2
HSALNG0131532	CCDC88B	SERPINA2
HSALNG0133406	CD200R1	AKT2
HSALNG0134941	ENSA	DNMT3A
HSALNG0141703	MMP28	HDAC4
HSALNG0141836	MRPS2	PLAU
HSALNG0141916	NPRL3	MAP3K7
HSALNG0142104	NUP85	SHH
HSALNG0143438	RHBDL2	BMP4
HSALNG0143806	PEBP1	ADCY3
HSALNG0144033	ADGRB2	FGG
HSALNG0144247	NFE2	PDE3A
HSALNG0144853	RFC5	MYCN
HSALNG0145450	SEPTIN4	NR4A2
HSALNG0146771	PLOD2	AIMP1
HSALNG0146880	CFAP161	P2RX4
HSALNG0147388	SAXO2	BRD2
HSALNG0147610	ALDH1A1	CADM1
HSALNG0147679	RUFY2	CTTN
HSALNG0147972	ARL5B	RAP1A
HSALNG0148037	CALHM2	TBX5
HSALNG0148984	CANT1	AZGP1
HSALNG0149127	CDCA3	PKN2

HSALNG0149391	DBI	GAB2
HSALNG0149401	DLX3	KCNE2
LARP1BP1	FRMD6	PRMT7
LOC105370032	LGALSL	STING1
LOC105370259	MRPL16	ADAM19
LOC105374775	MRPS16	AP3D1
LOC105377891	PPL	LTBR
LOC105378230	PPP1R9A	SMPD2
LOC107986583	RRP7A	BLVRB
LOC107987087	SLC47A2	SFRP2
LOC124901121	SGPP2	COL8A1
LOC124902513	ATP6V0D2	MSI1
LOC124902926	GPAT3	PIEZO1
LOC124902939	SPRR4	RBFOX1
LOC124903542	ADAMTSL5	TBCK
LOC124904947	CCNJL	MAML3
LOC653631	EED	PLA2R1
MIR12130	GPR161	TNFSF12
NONHSAG046902.2-001	HAUS2	ASTN2
RNU7-155P	MAP3K11	PPT2
SNODBsnoDB1676	SPRYD3	RGS20
SNODBsnoDB1822	SSH1	GLIS3
lnc-AGER-2	TASOR	HEYL
lnc-AMZ1-8	TSEN15	PDZD2
lnc-ANAPC2-1	WDR47	TENM4
lnc-ANKRD27-9	WWP2	FCHO2
lnc-ANXA6-4	AURKB	LINGO2
lnc-ARHGEF38-1	CPT2	SOX30
lnc-ATP2A2-4	AMD1	FEZ2
lnc-CASP9-5	BZW1	CCDC91
lnc-CFAP44-2	CD48	SPATS2L
lnc-CNIH1-3	CSF2RB	COMMD10
lnc-CPA1-1	DSTN	TMEM200A
lnc-CPQ-1	GAB1	ZKSCAN4
lnc-CRYAA-17	GYS1	ZSCAN31
lnc-CYS1-4	KCNB1	RAB4B-EGLN2
lnc-DIRC3-3	OSBPL3	ELN
lnc-DMWD-1	SEMA3B	TGFB1
lnc-EED-4	SMPDL3A	MMP9
lnc-EEF1G-1	TROAP	SERPINE2
lnc-EGLN2-2	CCDC78	PDE4A
lnc-FGF18-4	ABLIM2	TERT
lnc-FLT3-2	RCAN1	GSTP1
lnc-FPR3-1	MBD6	TLR4
lnc-GATA3-19-002	NLRC4	IREB2
lnc-GATA3-20	RAPGEF1	CFTR
lnc-GFRA4-1	EPB41L5	CRP
lnc-GPATCH2-8	MRPL12	AGER
lnc-GPC2-2	KRT17	IL6
lnc-HACD2-7	NTRK2	HSPA1A
lnc-HESX1-2	DEPP1	TP53

lnc-HIST1H1B-1	FDPS	SERPINA3
lnc-HIST1H2BN-1	ATP5IF1	CHRNA3
lnc-IER3-6	CAP2	SFTPD
lnc-ITGB6-4	DRAM1	SCGB1A1
lnc-KIF5B-6	GFPT2	ACE
lnc-KIN-10	ORM2	ADRB2
lnc-LTBP1-1	SLC7A11	CHRNA5
lnc-ME3-4	BCAT1	TSLP
lnc-MECOM-1	CPNE5	EGFR
lnc-MFAP2-1	FUOM	IL1B
lnc-MOAP1-1	KANK3	IL13
lnc-MOAP1-3	KIFC2	GSTM1
lnc-MSC-6	MARVELD3	LTBP4
lnc-NFKBIL1-9	MCPH1	IL10
lnc-NMBR-5	MESP1	SFTPB
lnc-NR1D1-3	SEMA4C	HDAC2
lnc-NR2C1-5	SLF1	HSPA4
lnc-P2RX7-1	INAFM1	LTA4H
lnc-PITPNB-4	KCNK7	MPO
lnc-PLAGL2-6	KLK13	CDH1
lnc-PLEKHA5-3	KRT83	TIMP1
lnc-PLEKHG6-5	LMTK3	PTCH1
lnc-POU5F1-3	LRRC46	IFNG
lnc-PXYLP1-3	RNF227	MMP3
lnc-RASSF10-9	RTL8C	GSTT1
lnc-RFX3-5	SERHL2	WAS
lnc-RSPH6A-1	ZFTA	EPX
lnc-RUNX3-2	ADH7	IKBKB
lnc-SERP2-16	AKT1S1	ABCC1
lnc-SERP2-6	BMPER	PKM
lnc-SERP2-8	CPNE8	GLA
lnc-SERP2-9	PDE7B	CDC42
lnc-SFTA2-13	SERPINB5	ROCK1
lnc-SOX30-4	SLC2A3	VCAN
lnc-SPATA31D4-7	SORBS2	LOXL2
lnc-SYT13-4	ST13	NR1H3
lnc-TAMM41-4	TRIM25	THBS1
lnc-THG1L-1	ZBTB16	DOCK1
lnc-THRA-1	SREBF1	HAMP
lnc-TMCO5A-3	DUSP7	LOXL1
lnc-TMEM129-2	IFITM1	PECAM1
lnc-TMEM267-1	RBP1	MCTP2
lnc-TRIM10-1	APOL3	AP2A2
lnc-TRIO-1	ATOSB	OSBPL2
lnc-TSPAN18-4	CBARP	PGAM5
lnc-WRB-1	GLT1D1	FNDC5
lnc-ZKSCAN3-3	KLHDC8A	CHID1
lnc-ZNF296-6	NDUFB11	MIR185
lnc-ZNF366-12	SGCG	MIR192
lnc-ZSWIM8-5	SLC10A6	MIR31
piR-30695	SLC25A42	MIR503

piR-31470-418	UBE2A	NALT1
piR-32214-479	UNC5A	MAPK3
piR-32287-088	ADAM10	ACE2
piR-33433	ARL4C	TAC1
piR-33458	ARRDC2	HAX1
piR-33947	CSE1L	CDK1
piR-34255	IL2RB	LIG4
piR-35002-056	MPZL2	POLI
piR-35674-292	NDUFB8	REV1
piR-36680	NEK6	HBB
piR-36774	NSD2	PI3
piR-36940	PALMD	FGFR3
piR-37468	PLA1A	CDH2
piR-37841	PRKAB2	DPYD
piR-38352-232	UAP1	BCHE
piR-38512-004	NEK2	HDAC3
piR-40336	TTLL12	SLC1A2
piR-40392	CHAF1A	CAMK2G
piR-41306-090	DCT	LIMK1
piR-41387	ELOVL3	MAP2K4
piR-41719	IKBKE	RAB7A
piR-44536	LBR	ADCY5
piR-45037	MRPS18B	CD44
piR-47162-172	OAS2	CSNK1A1
piR-48209-507	ZEB2	CSNK2B
piR-48456	CNTD1	DMPK
piR-49322-618	EPGN	GABBR1
piR-50443-044	H2-OB	PFKM
piR-50443-151	INKA1	POMC
piR-50675	NLRP2	RRM2B
piR-51137-090	ZNF23	THRA
piR-51912	ZNF562	ATP2B2
piR-52079-043	FIG4	BMP7
piR-52223	FSD1L	CACNA1D
piR-52276	MNT	CDC7
piR-52368-115	NAALAD2	CSTB
piR-52465	POLR3H	GATA6
piR-54299	RBCK1	GFPT1
piR-55045	AHDC1	HEXB
piR-55088	APBA1	HNRNPA1
piR-55194-394	NMD3	KIF5B
piR-55589	PLEKHG4	NR1D1
piR-56531	RP2	PGD
piR-56759-279	TOR1AIP2	POU5F1
piR-56979	TRDN	PPP2R1B
piR-57133-391	TSFM	RUNX2
piR-57285-002	ZMIZ2	TBX2
piR-57461-083	ARHGAP26	TWIST1
piR-59241	DHX58	ATXN3
piR-59547	KRT1	C2
piR-59897	PPT1	CDH11

piR-60812	RRP12	CDK12
AIDAP3	S1PR1	DLST
DUX4L45	CEP350	EHMT1
ENSG00000293114	COLGALT2	FPR1
ENSG00000293698	GNG4	HDAC7
ENSG00000294161	KCNC3	HLA-DRB1
ENSG00000296114	RPS6KA4	LYZ
ENSG00000296338	UPK1A	PPP1CB
ENSG00000296577	ATP5F1E	PYGL
ENSG00000296597	H3C4	SIK1
ENSG00000298029	KNTC1	SLC6A1
ENSG00000298189	LPAR2	SV2A
ENSG00000298460	MCHR1	TBL1XR1
ENSG00000298534	MMP17	TCF7L2
ENSG00000299593	NIF3L1	APPL1
ENSG00000300924	REG3G	BCL2L11
ENSG00000303287	SARM1	CSNK2A2
ENSG00000303819	VNN1	EHMT2
ENSG00000303850	ID3	ELOVL4
ENSG00000304564	CCNE1	HLA-DRA
ENSG00000305444	SAMD1	HMGA2
ENSG00000305663	ZNF44	IDUA
ENSG00000306329	STEAP1	PPP3CB
ENSG00000306359	FHDC1	PTPRA
ENSG00000306870	LRRC8B	SLC6A6
ENSG00000307517	PBDC1	SMARCE1
ENSG00000307923	RAP2A	ANTXR1
ENSG00000308058	SLC9A8	ATP13A2
ENSG00000309966	TSR1	ATP5PO
ENSG00000310186	U2AF2	C4B
ENSG00000310207	VPS35	CFL2
ENSG00000310337	BCL11A	COL6A3
HSALNG0009726	COLEC12	CRYAA
HSALNG0009727	IGKV14-111	DFFA
HSALNG0009728	IRF6	DLC1
HSALNG0010635	NPTX1	DOT1L
HSALNG0013187	PIK3C3	FZD3
HSALNG0013645	PLS1	HLA-G
HSALNG0022823	S1PR3	HTRA1
HSALNG0023536	SGMS1	IL17RD
HSALNG0023718	SPDL1	ITGA8
HSALNG0023719	VMP1	KAT8
HSALNG0024331	LHB	MAP3K12
HSALNG0025039	LSS	MAP3K20
HSALNG0026865	CLIP2	MGLL
HSALNG0031369	AASS	NCOA2
HSALNG0034444	ARID3B	NDUFA12
HSALNG0035227	CCDC85A	NNT
HSALNG0036164	COPS4	NRP2
HSALNG0036200	GNAO1	PICALM
HSALNG0036208	ILF3	POFUT1

HSALNG0042661	JADE1	PRKD2
HSALNG0044902	LRRC40	RUVBL1
HSALNG0046133	MRPS7	SENP1
HSALNG0046568	NUTF2	TBX3
HSALNG0048445	PAK4	TNFSF13
HSALNG0048965	PEAR1	TRIO
HSALNG0048968	RAB34	AMPD3
HSALNG0049191	RAB3C	APOL1
HSALNG0049192	RAB43	BCAR1
HSALNG0049239	RAVER2	BSCL2
HSALNG0049345	SLIRP	CALD1
HSALNG0049346	TOMM20	D2HGDH
HSALNG0049370	TPST2	DHX16
HSALNG0050781	XPO6	DLG2
HSALNG0051214	HK1	ESRRG
HSALNG0053115	LAMC2	ITPKC
HSALNG0056643	TPX2	KCNQ5
HSALNG0059985	FAM110D	KIF1B
HSALNG0062602	FAM229B	LETM1
HSALNG0062604	NXPE2	LGMN
HSALNG0067413	AFF1	MED1
HSALNG0076229	SLC16A5	NARS2
HSALNG0079166	PAX6	NDUFV2
HSALNG0081214	CHD2	PPP1R1B
HSALNG0083620	IRF8	RUNX3
HSALNG0094230-003	JDP2	SDC2
HSALNG0094277	KIF22	SFRP1
HSALNG0094551	SYNM	SPRY4
HSALNG0094569	ATG14	STX4
HSALNG0100593	CHIL3	SYT2
HSALNG0101509	CLCN6	TLE1
HSALNG0103272	CNTFR	TNXB
HSALNG0105090	DYNC1I2	TRIM32
HSALNG0107483	PACS1	U2AF1
HSALNG0107726	PHLPP1	VARS1
HSALNG0108050	PLCB2	VARS2
HSALNG0110308	RIC8A	ATP2A3
HSALNG0110309	SH2D3C	BMP5
HSALNG0111812	TBL3	EMP2
HSALNG0111819	TPPP	FGF18
HSALNG0112278	TPRA1	GAK
HSALNG0113992	WIPF3	GM2A
HSALNG0119962	ZFC3H1	HMGCS1
HSALNG0121255	CYP26B1	ID2
HSALNG0123135	RGS16	KPNA3
HSALNG0126147	TCF4	MAFB
HSALNG0126152	MSMO1	MMADHC
HSALNG0126153	ANK2	P2RY6
HSALNG0131536-002	NUP210	RPS14
HSALNG0135214	RPL15	RPS26
HSALNG0143222	GADD45B	ABCF1

HSALNG0145445	HSPA6	AP3B2
HSALNG0147483	ADGRA1	BACH2
HSALNG0147488	GIMAP1	DDX20
HSALNG0148998	SPRR2D	FLOT1
HSALNG0149747	STX10	FRS2
HSALNG0150710	AAMP	GNA12
LOC100422640	ANKRD22	HDLBP
LOC101927971	ANXA4	HOMER2
LOC102723568	ARHGAP31	MEIS2
LOC102723765	KCTD10	PCM1
LOC105370003	MYO9B	PROCR
LOC105371984	NECTIN2	RASGRF1
LOC105372711	NFKBIZ	RERE
LOC105372790	REEP4	RPL19
LOC105372804	STXBP2	SETD1A
LOC105374286	SULT1E1	SLC24A4
LOC105374894	TMEM30B	SLC30A10
LOC105374998	TPMT	SMPD3
LOC105375704	IL6ST	SPEG
LOC105376106	STC1	STX1B
LOC105376240	MFSD4B	TCAP
LOC105376806	SCN5A	TGFB1I1
LOC105377459	SRGN	TNPO1
LOC105377462	ZMYND8	TSEN2
LOC107985994	GSDMD	ADGRV1
LOC124900747	ATP6V1E1	AFF4
LOC124901389	CDKL5	AKR7A2
LOC124902783	CPXM1	BCL9
LOC124902784	EARS2	CACNA2D3
LOC124902795	FAM124B	CBX5
LOC124903267	GPRIN3	CCT2
LOC124903363	KCNIP3	CEP41
LOC124903364	MYCT1	CLIC5
LOC124904637	NUBPL	CNOT1
LOC124905976	OASL2	COQ8B
LOC124905989	SLAMF7	CPEB1
LOC124906355	TRAPPC9	DDAH2
NONHSAG045583.2	GMNC	DDX39B
NONHSAG046902.2-002	SPAG11B	EML4
SNODBsnoDB2073	ABCA2	FOXRED1
lnc-ABI1-8	APRG1	GTF2I
lnc-ALG9-1	CNN3	HSD3B7
lnc-BCAR1-3	MKNK2	IFT81
lnc-CHRNB4-3	RASSF4	KCNMB1
lnc-CNPY1-12	SYNPO	MN1
lnc-COL6A3-7	TXN2	MRC2
lnc-COPG2-5	ARMC3	NET1
lnc-COX10-2	DIABLO	NPAS3
lnc-DAW1-2	GP5	NTN4
lnc-DDX1-9	RTN4RL1	PCBP2
lnc-DIRC3-4	TJP1	PEX26

lnc-DIRC3-5	APLN	POLR2L
lnc-DLST-1-001	CAPRIN1	PPA2
lnc-DNAH5-4	CELF5	RASGRP3
lnc-GALNT12-2	COPS5	SEMA6A
lnc-GATA3-19-001	DDX42	SH3GL3
lnc-HLA-DRB1-3	MAP3K3	SMUG1
lnc-HMGN1-2	MDM1	SON
lnc-HOMER2-3-002	NXPE3	SOX11
lnc-KCNQ2-6	PLA2G4B	SVIL
lnc-LLPH-5	USP43	SYN2
lnc-LRMDA-6	TRP53	SYN3
lnc-MKRN2OS-2	PLK3	TAOK2
lnc-NAP1L5-4	HPCAL1	TCF7L1
lnc-NR1D1-33	PPRC1	THG1L
lnc-NTN4-4	RAMP1	TNRC6A
lnc-PVRIG-1	SLC37A4	AAAS
lnc-RAB28-6	ACAA1B	ADAMTS7
lnc-TIGD2-6	AP2S1	ARHGEF3
lnc-TMEM267-2	ARGLU1	B3GALT4
lnc-TRIM15-2	CTDSP2	CC2D2A
piR-30091-163	KCTD5	CCNT1
piR-31936-005	LDLRAP1	CEPT1
piR-32214-583	LRR1	CLIC4
piR-32264	LRRC17	DGKB
piR-32677-458	METTL1	DGKG
piR-33303-154	NAT8L	DYNC2H1
piR-33614-090	NDUFS2	EEF1G
piR-33804-054	PKNOX2	EHBP1
piR-33826	SLC35B2	FKRP
piR-33978-008	SYTL1	GNG2
piR-34093-011	SYTL5	GPX5
piR-34822-045	TNNT3	HERC1
piR-34822-244	KRT9	HIVEP2
piR-35564-034	AKR1E2	HLA-DMB
piR-36240-110	APOBEC3G	HMGN1
piR-36393-498	CD300LG	ID4
piR-36531-017	CHMP7	IFT43
piR-38351-331	EFCC1	KCNS3
piR-38352-172	NGP	LRRC32
piR-41008	SCGB3A1	LTB
piR-41183	UPK3B	MORF4L1
piR-41306-189	LMO7	PADI3
piR-41306-195	MVD	POMP
piR-42384	PSEN1	RHCG
piR-42491-197	PTK2	RND3
piR-42694-089	SEMA3E	SLC5A3
piR-42777	TRIB1	SLC7A6
piR-43083-141	CD19	SRCAP
piR-43099-560	FKBP3	TRIM27
piR-43105-241	GBP6	TRIP11
piR-43106-297	GPBP1	UBR4

piR-43106-342	HELZ	VPS45
piR-43107-030	IGSF11	AFAP1
piR-43583-524	MRPL38	AP4M1
piR-44851	PAQR5	BAG6
piR-44878-010	PIAS3	BOC
piR-47628-090	S100A16	CASZ1
piR-47628-120	CARHSP1	CHI3L2
piR-47796-002	LPP	CPQ
piR-48553-160	PFKL	DOCK10
piR-48695	CYP51	EFL1
piR-48773-005	HPGD	GALNT12
piR-48916	PLD1	HLA-DMA
piR-49423-064	SDC4	INCENP
piR-50443-201	CYP2D11	INPP5J
piR-50444-346	MIR652	ITPK1
piR-50444-422	ZNF699	KLHL7
piR-52079-101	MACF1	MED13L
piR-52388	NID1	MICA
piR-53006	RPL17	MYO9A
piR-53177-009	ACAA2	NCALD
piR-54592-283	CD33	NIPAL4
piR-55281-583	COL5A1	NOVA1
piR-55650-604	EEF1A1	NUMBL
piR-55655-370	IQUB	OASL
piR-55655-532	MCEMP1	PELI1
piR-55948-035	ABTB3	PLXNC1
piR-56022-529	APBB3	PRSS23
piR-56197	EDA2R	RSPO3
piR-56759-241	ESYT2	SLC14A2
piR-57133-360	JPT2	SLCO4A1
piR-57133-427	KIF26B	SLMAP
piR-58297-301	MLXIP	SMTN
piR-58997	MRPL23	TSEN54
piR-59297-224	MTMR4	UPF2
piR-59412-008	POLR1D	ZFPM2
piR-59769-220	PREB	AAGAB
piR-59769-457	RASAL2	ABHD16A
piR-60146-109	RPL41	AKAP6
piR-60597-069	RTN2	AP3M1
piR-60985-126	SIPA1	ARHGEF17
piR-61029-088	SNRPD2	ATP5MC2
piR-61101-576	SOX13	BMP8A
piR-61240-088	SOX7	CAPZB
piR-61945-028	TRAPPC6A	CD53
piR-61945-302	YTHDF2	CENPB
ENSG00000299180	BOK	COL15A1
ENSG00000300700	F13A1	COPZ1
ENSG00000303489	GLS2	CRYGS
ENSG00000303941	GSDME	ECD
ENSG00000307550	MELK	EEFSEC
ENSG00000308052	MYBBP1A	EIF3B

HSALNG0013192	APEH	ERC2
HSALNG0019782	PREX1	GALK2
HSALNG0022798	CAPZA1	GATA5
HSALNG0022800	CEP152	H1-5
HSALNG0030563	DEF6	HESX1
HSALNG0049127	ERGIC1	HLA-DQA2
HSALNG0049430	FMN1	HMCN1
HSALNG0086173-001	GLIPR2	HNRNPUL1
HSALNG0089708	GLIS2	IER3
HSALNG0091489	GORASP2	IL18RAP
HSALNG0093267	LGALS4	KIFC1
HSALNG0107761	MANSC1	LSM2
HSALNG0119622	MGAT1	LSM3
HSALNG0142605	MON2	LY75
HSALNG0143659	PBX2	LY86
LOC101928570	RAP2B	NEBL
LOC105372017	SKA2	NELFA
LOC105372926	TCF25	NEURL1
LOC105377721	HNRNPU	NPNT
LOC105377855	LSP1	NSRP1
LOC105378523	NPAS2	PARP6
LOC107986462	PVR	PES1
LOC124902368	RPL10A	PKNOX1
LOC124902741	STEAP4	PPP1R10
LOC124907774	KCND1	RBFOX3
LOC124909356	IFIT1	RBMS1
SNODBsnoDB1224	C2CD4B	RELT
lnc-MICA-7	NCCRP1	RNF220
piR-32314-014	NRSN2	RNF40
piR-33432-200	TRIM17	SCARF2
piR-33605-171	ZNF83	SENP3
piR-37972-677	BICDL2	SF3A1
piR-43107-111	GIMAP5	SH3TC2
piR-48325-029	SYVN1	SLC36A1
piR-52680-075	TRIM46	SMG6
piR-56037-030	ARHGEF2	SRRM1
ENSG00000293947	CAD	SSR1
ENSG00000298046	ECHS1	TMTC1
ENSG00000298114	FAM83D	USP3
HSALNG0066075	NT5DC2	WDR4
HSALNG0081753	STARD4	ZFP57
HSALNG0094280	FYN	ZKSCAN1
HSALNG0113311	PDCD4	AFF3
HSALNG0132819	ICE2	APCDD1
LOC101929774	ATL3	ATF6B
LOC105370195	CD37	BCAS3
LOC105377327	CRLS1	BTN2A1
LOC105379147	CTSF	C1GALT1
LOC105379164	MTMR7	CALML5
LOC107986051	NECTIN4	CCL28
LOC124901340	NMNAT2	CNTN5

LOC124903348
LOC124904367
LOC124904603
lnc-USP33-3
piR-32677-129
piR-36393-135
piR-42700-014
piR-42700-035
piR-43099-141
piR-45504-142
piR-47132-002
piR-48348-061
piR-51878-031
piR-55654-324
piR-55655-136
CR1
ADCY10
IL9R
NKX2-1
SMC3
SMARCAL1
AICDA
PARP2
ERCC8
RNASEH1
NHEJ1
RPL35A
TOP1MT
DHX36
SMARCAD1
SMC5
CDC5L
CUL7
H2BC21
PIAS4
RECQL5
H3-4
MYSM1
RMI1
SHOX
SPO11
DDX47
SLCO6A1
CCDC8
FAAP24
RNF212
BOD1L1
DCLRE1A
ETAA1
H3-5
HELB
NPLOC4
SCAP
SF3B3
TPD52L2
CPM
NISCH
ADD3
FERMT2
LRP2
SRSF2
TNS3
CHIL4
KLRA4
STAT5B
ADCY9
BAG1
GLRX3
MAPK6
SCARA3
TMEM158
ADORA3
CD3E
CD59
COPS2
EHBP1L1
FAM117B
LCP2
OLFML1
POPDC2
PRXL2B
RNASEH2B
RPL39
RRBP1
THEMIS2
CAPSL
CDIN1
CRACDL
DGKQ
DMRTA2
GATD3
MS4A6C
NCKAP5L
PRKAG3
TEKT1
UPK3A
WDR55
AKR1B1
FSHB
DOCK9
ARPC1B
FMO1
CPA4
CPEB4
CSAD
EXOSC10
FRAS1
HOXC6
KIF13A
KLF11
LRIF1
MFHAS1
MYOZ1
NAV1
NDST2
NMU
PFDN5
PIDD1
PILRA
PLB1
PSMG1
RIOK1
RSRC1
SETDB2
SF3A2
TRIM31
VPS54
WDR73
ZNF74
GDPD3
GPC2
KLF12
MAML2
OTUD4
PPARGC1B
RNF34
SESN1
SH3PXD2A
SRPRA
TRIM39
TXNDC5
ACAD10
ADAMTSL3
ALKBH5
BLOC1S5
CHIC2
COL20A1
CRIM1
CRLF3
CROCC
DPH7
EPC1
GPX6

TERB2
SWSAP1
MDH1B
MAJIN
TMEM203
H3-7
LLCFC1
MEG8
BIVM-ERCC5
DNAAF4-CCPG1
HIRA
WIPF1
DOCK8
TMC8
DNAH9
DYNLT5
KCNJ1
PIK3C2A
RAB5A
ALG1
GCDH
SLC26A3
CLCN2
FCN2
PSMD4
RARS1
ALOX12B
SLC52A3
TREH
YME1L1
ABCC11
AHSA1
ASAH2
CTNNA3
PRSS2
ABCB10
ABCB8
P2RY4
SGCB
NCBP1
SELENON
SNX27
CLPS
GUCA2A
ACRV1
LTN1
KRT33A
DNAJB12
ASIC5
DNAJB14
DEFB104A
NAV2
RASSF1
MYEOV
AOPEP
BUD31
CACNB4
CLEC4E
DCTD
GABPB1
NXPH3
PCBP4
PXDC1
RPL29
SLC16A11
SLC45A4
TAF4B
TRERF1
TRIM47
UHMK1
VPS13B
WDFY1
NPM1
GLIPR1
KNL1
MYOF
NOP56
VAV3
CCDC9B
LAPTM4A
UBE2J2
CPLANE1
E4F1
NAT9
TUBGCP6
WFS1
DMXL2
FBXO31
GPC6
KLC1
MAT2B
MBTPS1
MTFR2
PRR13
RBM19
ST6GALNAC2
FMO2
LBH
LRG1
PYGM
SERINC2
KIF20B
HOXC8
ME3
MLEC
MTG2
PLEKHG6
PWP2
RBP7
RCAN3
SCARA5
SEPTIN8
SHB
SNRPF
UVSSA
ARHGAP42
ASAP2
ATP8B4
CFAP43
CXXC4
DMRT2
DPF3
FCHSD1
FNDC3B
GNL1
HPCAL4
HSF2BP
LST1
MPHOSPH9
MRPS27
MRPS6
MTCL1
NFKBIL1
NUDT5
PHF11
POLR1H
PPP1R12B
PPP1R3B
PRRC2A
PXYLP1
RAB11FIP4
RPS6KL1
RPUSD4
SLC66A1
SSR3
SUPT3H
TMEM219
TNP1
TRIM26
TSPAN14
UNC119B
USB1
ZBTB38

MUC3B	KRT10	ZNF668
MIR323A	LRRC37A3	ANKRA2
CFM1	PRLR	CFAP20
LOC113219471	CD2AP	CFAP44
LOC113633875	CD3G	CLASRP
LOC113633876	CLEC2D	COL21A1
LOC108491823	COL8A2	DMWD
LOC111674464	GABRP	ELK3
LOC111674465	GBP3	GRPEL2
LOC111674466	IMPACT	GSDMB
LOC111674470	KCTD1	HOXC10
LOC111674471	LYSMD2	LYPLAL1
LOC111674473	STEAP2	MEGF11
LOC111674474	ZSWIM6	MEGF6
LOC111674476	C2CD3	MICAL3
LOC111674478	CYTOR	NAP1L5
LOC111674479	LSM1	NELFE
LOC113523647	TRIM16L	NIM1K
LOC113604967	ZNF24	NUFIP2
LOC113664107	FLACC1	PLA2G2E
MIR451A	H2-T23	PLRG1
PWAR4	HEPACAM2	PSAPL1
SOCS5	IL36RN	RBMS3
IL17C	IQCD	RHOBTB3
NAF1	NIPA2	SERTAD1
DEFA3	PIGZ	SIDT1
MIR125B1	PPP6R2	SSH2
MIR125B2	RPS2	SYMPK
MIR486-1	HNF4A	SYT13
RPL36A-HNRNPH2	AHRRA	TBC1D10A
LTB4R2	ANKHD1	TCF19
IFNL1	ANO6	TMEM106C
MIR196A2	ARL8B	WDR20
MIR92A1	ETV1	WHAMM
MIR519D	GAS2L3	ZCWPW1
MIR92A2	LAT	ABLIM3
LINC00612	LY6D	ARHGEF37
MIR570	NRN1	CCDC69
MIR486-2	PALM2AKAP2	CCDC97
TRV-AAC1-4	PLXNB1	CELF6
TACR3	POLR3E	CLDN23
SNRPN	PROSER2	FGD6
BHMT2	RAB38	GFRA4
GNGT1	RASD2	MED24
PCDH15	RNF128	MED7
CHRNB3	STRA6	MFSD1
CYP3A43	UBE2S	MTMR11
DAAM2	UQCR11	MUCL3
TRIM71	VRK1	PCYOX1L
PLXNA4	NR3C2	PHRF1
DEFA1	PRDX5	PPP1R18

AFAP1L2	INIP	REM1
CNTLN	MXD4	RNF166
TAS2R1	MZT2A	RPAP3
RHBDD1	RIPK1	TMEM260
RLN1	AGO2	TRIM15
ZBTB9	FLNC	TRIM62
SOWAHB	SLCO2A1	TSPAN4
LINC00336	CCDC88A	ZBTB42
MIR140	CHAC2	ZDHHC18
MIR429	CRLF1	ZNF131
C15orf32	EML1	AFAP1L1
MIR200B	INTS6	B3GNT8
MIR373	PSCA	BET1L
MIR101-1	SLC38A10	CD2BP2
MIR124-1	SRPK1	CDC20B
MIR199B	THRAP3	CNPY4
MIR675	TRMT61A	COL28A1
MIR103A1	CDR2	DENND2D
MIR144	FBXL20	GPSM3
MIR379	GCNT4	H3C11
TTN-AS1	MDP1	HOXC9
MIR1343	PANX2	IP6K3
MIR320B1	POGK	IRF2BP1
SNORD44	RUSC1	KANSL2
MIR320B2	TDP2	MSL1
CCL15-CCL14	TLCD5	NRM
LIMASI	TTYH3	PAQR8
LINC00882	TUBGCP2	PRR14
MFF-DT	DYNC1LI2	RAPGEF6
PWAR6	EFTUD2	RBM33
MIR612	FBXO2	RSPH6A
TRE-TTC3-1	FCAMR	SEC22A
HSALR1	FGD3	SLC45A1
KIAA1614-AS1	KDM6A	SSNA1
MIR4485	KMT2D	TSPAN3
MIR4443	MASTL	UTP15
IGES	OLFML2A	VGLL4
RPL34P26	PAK3	WDR46
ISCA1P6	PPP1R2	ARAP2
RPS28P1	SRPX2	CCDC61
STARP1	TBC1D16	CMSS1
RPL23P11	TTC28	DTWD1
RPL6P14	ZBTB43	EGFL8
IFN1@	EIF4A2	ENDOU
NQO1-AS1	STAT2	FSTL4
PRKACB	PDLIM5	GPATCH2L
GABRA2	SNORD56	PAN3
KLK1	ATP9B	RAPGEFL1
MC4R	CBR2	RPAP1
KCNH5	CCDC57	RRP15
SLIT2	CDC14B	SLC35F3

EMX2	CES2B	SLC48A1
FGF4	CFAP52	TRMT61B
GNAT2	CHAD	ZNF740
MSX2	ICAM4	DDIT4L
TRPS1	KRT75	FAM184A
CD96	LAGE3	FBRS
RHOH	RBM45	GPANK1
SNCG	SNAP47	INO80E
C4BPB	TANGO2	MICOS10
DSCAM	TGM3	NELFB
LIPF	TMEM167A	OXCT2
LMX1A	WDR82	PILRB
MIPEP	ZMYND19	PRXL2A
NRG3	BBS1	RAB20
RYK	ADRA1A	RGS7BP
SIGLEC8	OGT	RUSF1
SLC27A6	RBM3	SELENOM
SLC30A8	AMPD2	SYF2
VAMP8	CLYBL	TM6SF1
IAPP	DPP9	TMEM50B
IGSF1	FXYD3	TRIM10
MAP3K13	HOXA5	VIT
MMRN1	KCNE3	ZC2HC1C
PPIG	LHFPL2	ZNF362
TRPC5	MAP1A	ZSWIM8
CLDN6	NONO	ARHGEF38
FUT9	PLCD3	ASB8
KCNJ9	PRKAR1B	EBPL
PCDH10	SLC16A12	HSPA12B
RPH3A	SLC44A4	IFFO1
SNX9	SP4	MIGA1
CADM2	TDG	PDRG1
USH2A	TIMM10	PHF13
LRRTM1	TNS2	PLEKHJ1
NETO1	CYP6G1	RANBP6
RGS8	GPS2	RASGEF1C
SEZ6L	HES4	SH2D4B
SOX1	ADAMTS1	SPDYA
TMEM30A	SNHG7	SPPL3
KCTD13	TSC22D1	TIGD2
KHDRBS2	ALDH1L2	TMEM80
PPFIA2	SCARB2	TMPPE
ADPRH	GMPR	USP35
CSNK1A1L	HLCS	VWA7
EIF4ENIF1	MAMDC2	ZBTB12
RNF41	MRTO4	ZNF689
ZW10	NDUFS8	ZNF747
BRINP1	PLPP2	ZNF780A
IL26	RNF13	AMZ1
MUC7	RNMT	CFAP54
NMUR2	TC2N	CORO6

PTCHD1	ELK1	CTXN3
C14orf39	ARL11	DCAF4
MAGEC2	C1QTNF7	GAL3ST2
ZWINT	COG2	HAUS3
BTBD16	ELAC1	IQCH
DMAC1	FAM216A	MBLAC1
FEV	MEA1	PID1
GPR15	MED29	PRDM11
MAGEC1	MIR20B	RASSF10
ZNF536	MRPL36	SERP2
CCDC174	OLAH	SERTAD3
LRRC49	PLPPR2	ZNF764
OTOL1	SLC23A3	ZNF780B
LEPROT	UBE3B	ZSCAN12
OSBPL6	YIF1B	CASTOR3P
SCFD2	CYSRT1	CCDC184
SYT9	KRI1	FAM98A
XIRP2	NCKIPSD	HMGXB3
ATRNL1	CAPN6	LLPH
BARHL2	CDCA2	PLAC9
BRINP3	LRRN3	PRAG1
CYLC2	LUC7L	SAPCD1
JRKL	MADD	SPMIP8
LIPM	ME2	TRIM4
MAP3K19	MNS1	XKR9
TAS2R14	NADK2	ZCCHC10
CFAP47	PLOD1	ZKSCAN8
SPINK13	SAMD9	ZNF785
TDRD10	SF3B4	ZZEF1
USP17L2	SLC27A3	RSRP1
ZNF607	SLC38A5	SERTAD2
DLEU7	TANC2	EPOP
LRATD1	ZNF821	PRR3
C12orf42	H1F3	QSER1
DEFB127	PRNP	ZSCAN16
INSYN1	CMBL	ZSCAN26
C4orf33	GLI1	FAM227B
DEFB118	GSTM4	PRR14L
OR12D2	NOLC1	RUFY4
DDTL	PAICS	TMEM116
DCDC2C	PDLIM1	ZNF688
DCAF8L1	KIF9	LY6G5C
LINC02914	ATP5MG	MIR615
PPP1R2C	ATP6V1B1	MKRN2OS
MIR130A	BBS5	CCDC192
MIR196A1	BCAM	MIR4435-2HG
EPB41L4A-DT	CALCOCO2	TEX41
HULC	CCDC12	LINC00243
LRRC9	CDC42EP4	NPHP3-AS1
MIR138-1	CELF1	MIR648
MIR148B	CMTM3	LINC00857

MIR200A	EGFL7	MIR3936HG
MIR370	ELOF1	NPIPB13
MIR491	EML3	CROCCP2
MIR99A	FAM3D	DM1-AS
ANP32CP	GDF2	HCG20
HOTAIRM1	IGF2BP1	ITPK1-AS1
MIR409	IQSEC2	MBL1P
HOXA-AS2	KCNAB2	SNORA52
MIR103A2	NACA	SMIM33
MSL3B	PCSK1	ZKSCAN8P1
MIR217	PPP1R16A	CYP2B7P
MIR365A	RAB11FIP1	TNPO1-DT
MIR885	SBK1	LINC02547
PCAT1	SEMA4B	SNRPF-DT
FOXC2-AS1	SEMA4G	CYP2T1P
LINC00482	SERPINB1A	LINC01679
MIR124-3	TOX2	FAM86B3P
MIR153-1	TSPAN18	RPL7P22
MIR422A	WDR6	NPM1P35
MIR500A	PTTG1	CSF1
MIR517A	BST2	SESN2
MIR532	GTSE1	MIR150
MIRLET7F2	PNP	ATR
PCAT2	RPS27	DNMT1
CCAT1	AMDHD2	GAPDH
LINC01405	ANG	RARA
MIR101-2	ASAH1	MYB
MIR124-2	CDC42EP1	HPRT1
MIR153-2	CNTRL	SIRT2
MIR502	EEF1E1	UNG
MIR551B	FKBP1A	CCL17
RPLP0P6	NCAPH	PIF1
MIR194-2	NDUFV1	ADAM17
MIR320C1	PHACTR2	ENTPD1
MIR450A1	PPAN	ALDOA
MIR637	RAP1B	AHCY
LINC00861	RASGRP2	SMPD1
LUZP6	SEPHS2	IL17RA
MEIS3P1	SLC25A36	CEBPB
MIR1226	SLC4A5	EPRS1
MIR613	UQCRH	ADCYAP1
MIR656	GALNT4	LMNA
THRIL	CCNG2	PHGDH
MIR889	CCDC9	PRKDC
SNORD50A	CLEC4N	TOP2A
UBE2DNL	CUTA	RPL5
MIR320C2	HARS2	TP53BP1
MIR519A1	HECW1	TOPBP1
LINC00314	LYRM9	PLA2G4A
MIR1301	MOB3C	ENO1
MIR517B	OTUD5	GRN

MIR548D1	ZBTB22	TLR7
SNORD38B	COL6A2	ADORA2A
HIF1A-AS3	E2F2	TPI1
MGC12916	FUS	BSG
MIR1197	IRAK2	CSTA
MIR1208	SLC39A8	NAT1
MIR1293	CD3D	STUB1
MIR2113	CLIP4	PDIA6
MIR300	DEDD2	CCL22
MIR3147	FAM114A1	ARRB2
MIR365B	HIF3A	GRK6
MIR421	ITPA	CALR
MIR450A2	PDCD11	LDHA
MIR519A2	PLEKHA6	ALPL
MIR548D2	PLXND1	ITGB1
MIR630	SAR1B	P4HB
MIR2110	SLC37A2	SGK1
MIR3148	SLC43A2	GSN
MIR558	STIL	MYD88
ZDHHC8BP	STON2	PGK1
GOT2P1	TGOLN2	PIK3CB
KLRK1-AS1	ALAS1	YWHAE
LINC01924	ASS1	FASN
MIR3123	FAM124A	HSPG2
MIR3182	IRS2	ITGAV
MIR448	PCDHB22	MUSK
RNU2-1	ADRA1B	ADORA1
SNORD49A	PDK1	ANXA2
TRC-GCA24-1	RBM25	CAST
MIR650	ARID5A	FLNB
MIR761	CDC42EP2	GPI
CYP51A1P2	CSRNP1	HSP90B1
MIR2054	DDX17	MDH2
MIR3163	FGFR4	NME1
PDE9A-AS1	GEM	YWHAB
PLEKHA3P1	HNRNPK	YWHAZ
TTC4P1	ICA1	CA4
GLYATL1P2	KCNMA1	DDIT3
LOC440982	MCU	IL1R2
NASPP1	MYO1C	MYH6
PTMAP5	NDE1	TKT
RPL10AP3	NFIX	TRAF6
RPL21P50	OSR2	VDAC1
RPL21P94	PHKB	HYOU1
RPL31P12	PIK3AP1	PTGIR
RPL36AP29	PLEKHA5	ATP5F1B
MIR6504	SGO2	P2RY2
RPL31P54	SHMT2	PDIA3
RPS17P12	TMC6	AKAP13
AKR1B1P2	TPPP3	RACK1
COX5BP1	WASF2	VDAC2

CYP51A1P1	CILP2	ANXA3
GNPATP	DOP1A	SP3
MIR548AY	GPR137	HSPE1
MRPL49P2	GRTP1	MSRA
PRDX1P1	H2AC4	PTGES
RAC1P1	H3C15	MARCKS
RAC1P5	REXO1	MVP
RPL21P99	RHOT2	PSMC6
RPL29P6	SH2B1	TAGLN2
RPL31P31	SYNE4	VAMP3
RPL9P21	ZNF385B	RPL32
RPS9P3	THRSP	SEPTIN7
SLC6A6P1	INHBB	LPCAT1
ST2	NPC1	NHLRC2
FAM8A6P	ROCK2	MUC3A
HPRT1P2	ATP11A	MIR30B
HSPA8P1	C2CD2	ABL1
HSPD1P9	CCT6A	CDK2
HSPE1P16	JCHAIN	ACTB
LIN28AP1	NPM3	KAT5
MIR5708	NRBP2	SMARCA2
MIR6852	PITPNC1	TOP1
MRPL50P1	RASL11A	BUB1
MRPL57P6	SPTLC3	CTBP1
NFYAP1	TUFT1	RPL11
OR7K1P	ZCCHC7	WEE1
RPL29P29	SUGT1P1	DCTN1
RPL36P16	CSRP2	MCM4
RPL6P25	EIF2S1	RPA1
RPL7AP53	FMO5	RPS19
RPLP1P9	GMNN	UBE2I
RPS12P24	NDUFS1	ATRX
RPS23P3	PPP2CA	BUB3
RPS26P13	RETSAT	CDC45
RPS27AP4	AASDH	PML
RPS3AP9	AP5Z1	PNKP
RPS3P6	ARTN	SUMO1
SRIP1	CEP250	UBE2N
SUCLA2P2	CTRL	HELLS
VENTXP2	CWC25	PIAS1
VN1R95P	DGCR6	TDP1
IPMKP1	FAM161B	XRCC6
ISCA1P2	FOXJ2	DDB1
LOC101928236	GBA2	FMR1
NUP50P3	IQCE	HERC2
RNU7-51P	KCTD20	NCL
RPL31P20	LGI3	RAD21
RPL36AP10	NKAIN4	REV3L
RPS15AP40	NUDT14	RFC2
SGCEP1	PITPNM3	UBE2T
TBCAP1	SCO1	USP1

KRT8P16	TECPR1	APTX
RPL34P25	YBX2	ATRIP
PSMA7P1	ZNF224	CDA
LOC101928912	MAST3	CTBP2
LOC102724802	DYRK3	DCLRE1C
LOC107832851	ELOVL7	DHX9
CFAP221	GOSR2	H2AX
	GPRC5C	MAD2L2
	GSTP2	MCM6
	IFRD2	NHP2
	IVL	POLL
	KRT16	RAD17
	MAP7	RBBP8
	MAP7D1	RPA2
	MDN1	SRSF1
	PHF20L1	TOP3A
	PLBD1	BANF1
	SLC20A2	CCNA1
	TNKS1BP1	CDT1
	WDR77	PCNT
	ZMYM3	PRIM1
	TGTP2	RNF168
	ZNF761	RPS24
	CDCA8	SAMHD1
	CHAC1	TREX1
	ABI1	DDX11
	APBB2	DNA2
	ATF7IP	PARG
	ERMP1	POLD3
	GDF10	POLQ
	HEMK1	RECQL
	KANK4	RNF8
	MIDN	SETX
	PLA2G4C	SSBP1
	PSMD2	SUMO2
	PUS1	TWNK
	STAU2	ZMPSTE24
	TMED2	HLTF
	TNFAIP8L1	MCM8
	TNNC2	MUS81
	TPCN1	NOP10
	UBE2B	POLM
	ABHD8	RAD18
	H3C10	RAD54B
	BIVM	RAD9A
	CAMSAP3	TIMELESS
	FBXL12	UIMC1
	FBXO41	CDCA7
	FHAD1	CLSPN
	KCNE4	FIGNL1
	MGRN1	GINS1

MNDA
MRPL53
PAF1
PCDHAC2
PKHD1L1
SKIC3
SMCR8
STK16
ZFYVE27
CD38
DHX40
H2AC11
MAMDC4
MGST2
MRNIP
SLFN2
SRP54
TMEM132D
ACP3
ADI1
AVPI1
CFP
COX3
EPHA1
IL2RG
KANK2
NUAK1
PLP2
PRRC2C
SDS
SIVA1
SRRT
FIS1
NSUN5
SLC19A2
C1QB
JUND
RND1
USP18
ENTPD8
INO80B
MFSD10
PCDH17
PLEK2
SLC38A1
ACKR2
AKAP8L
DISP2
EFCAB11
EFNA5
FBRSL1
HFM1
NEIL1
RFWD3
RPA3
SP100
TOP3B
USP25
YBX3
FAN1
H3-3B
MSH4
PAXIP1
UBE2V2
DDX18
GEN1
MCM10
NEIL3
OBSL1
RIF1
SUPV3L1
TIPIN
CTC1
EME1
ERCC6L
H2AC20
HELQ
MTREX
TTF1
WDHD1
XAB2
CGAS
DDX39A
NOL8
CENPS
FBH1
LSM11
MPP2
POLN
SMC6
SPRTN
TCHP
WRNIP1
APLF
FAAP100
H3C13
POLD4
TDRD3
ZRANB3
RMI2
SPIDR
STK11IP

FCGR1	EXD2
FCGR3	HROB
HAPSTR1	PAXX
HPGDS	EXO5
HS3ST1	H2AC18
KRT6B	CENPX
LSM12	FAAP20
LTK	FSBP
METTL18	COL4A3
MICAL1	MIR494
PHF23	RELA
PITHD1	NCF1
RPF2	NCF4
TBC1D7	PTPN22
TMEM125	UFD1
TNNI2	GP1BB
TOM1L1	JMJD1C
UQCC1	ALMS1
ZFYVE19	ARVCF
ZWILCH	CARMIL2
FOXO3A	CYBC1
H2-OA	MPEG1
KIAA2013	NGFR
AIRIM	GAS5
CCL21A	GRK2
CIBAR2	CSF3R
EHHADH	SAG
AKR1B8	ARRB1
EIF5	GRK3
FBXW5	G6PC3
MEDAG	ACKR3
NQO2	DNALI1
OSBPL7	DNAAF10
PLEKHM2	PIH1D2
SCD	IDH2
HEPH	JAK1
EPPK1	GAA
FXN	VIM
IRF4	FTH1
ITIH5	G6PD
MYL4	KCNQ1
PRKCG	PRKG1
RAB8B	TUBB3
RAPGEF2	ACTN1
RNASEH2A	ENPP1
SLBP	FLNA
ACSL5	GUSB
DHCR7	HSPA8
CXXC1	PRKAA2
KLHL18	SLC12A2
KLHL6	VCP

MAGIX	ACTA1
PISD	EEF2
SLC30A6	FURIN
YIPF4	HSPA9
ASB7	IMPDH2
CCNYL1	ITGA3
CHPF2	ITGB4
RNF123	PDPK1
SHISA4	PKD2
SHTN1	RDX
SNAPC4	SLC16A1
SPTSSB	SLC40A1
TRABD	SLC4A1
TXLNB	SPTLC2
ZNHIT2	TPM1
FAM83H	UBA1
RILP	XPO1
BHLHE41	YWHAG
GATM	ACSL4
GREB1	ARSA
PTP4A1	ARSB
YAP1	CAPN2
COX7B	CLTC
EWSR1	GALK1
FNBP4	GJB1
GPM6B	HUWE1
MTCH2	ITCH
RNF125	MASP2
TUFM	MC1R
YPEL3	MSN
AIFM1	MYH7
GAL	PAH
ADD1	PDP1
MIR450B	PLCB3
SLC26A2	PNLIP
ADSS1	RRM2
DDX60	SERPINH1
HEG1	SLC4A4
MRAS	SLC5A1
NRXN2	ABCC8
PPIC	ACADVL
PPP6R1	ACLY
PSMC3IP	AQP3
CASKIN1	CDH3
CHAMP1	CSK
CIC	ETFA
CNIH2	FHL1
CTR9	GANAB
CYB561D2	GJB2
EDARADD	ISG15
GOLGA8A	ITGA2

HAUS7	KCNJ11
HBA-A2	LAMB3
HOXA11	MYH11
KCNRG	MYH14
MFSD2	P2RY1
MPPED2	PFN1
NEK4	PRDX2
NOP14	PRKG2
OSGEPL1	PSMB4
PANK2	SCP2
PCDHGA3	SLC25A13
PCDHGA4	TPM2
POLR2D	UGDH
RABEP2	YWHAH
RPGRIP1L	ATP2B1
SLC12A9	ATP2B4
SLC26A11	CKM
CCDC87	CLCN3
PNPLA2	DMD
PODXL	EMD
SPDYE2	FHL2
AGPAT3	GJA5
DSG2	GUCY2C
GPSM2	LMAN1
SNTB1	MAP1B
ADAM12	MARS1
ANGPTL2	PKD1
CABLES1	SLC25A3
CHRDL1	SLC9A6
CTSZ	TFR2
PAK2	ABCB7
CFAP251	AMFR
LMF1	ASIC1
PIGQ	CLCN5
KLC2	CTRC
FDFT1	DNAJB1
GADD45G	DNASE1
GNAI1	EIF2B4
LY96	FKBP8
AOC2	FSCN1
C1D	G6PC1
DENND1C	GJA8
FBF1	GLO1
GLB1L	IARS1
GTF3A	IGF2BP2
INTS2	LAMA2
LIN54	LAMP1
LRRC34	NHERF1
LRTM2	PPP1R12A
MMRN2	PRKCSH
PTPRCAP	PRSS1

RNF11	PRSS8
SHPRH	PSMA3
BLVRA	PSMA7
CAPNS1	PSMB1
GPR155	PSMC3
MOV10	PXDN
MSX1	SKP1
SPRED2	SLC1A5
SYTL2	VAPB
TOMM40	AARS1
CIDEC	ADRB3
DHRS3	BEST1
IDH3A	CALM2
LORICRIN	CCK
LUM	CDC37
MEGF9	COPA
PTCH2	ENTPD3
CTHRC1	IL7
GMFB	KRT13
MAPRE1	KRT7
PDGFRL	LAMA5
TRAF4	LAP3
TUBG1	LMNB2
PRMT9	MYL1
ZNF256	MYL9
NAIP2	MYO6
ZNF419	PABPC1
ZNF70	PLEC
ADAMTS10	PSMB5
CD300LF	PSMD14
CNOT3	PSMD7
DDX59	PZP
DUS2	RPN1
FMNL1	S100A10
INTU	SURF1
KCNC4	TRIM21
MORN2	TUBB6
MRPL32	AP1B1
MS4A6A	AQP9
PRSS22	CALM3
SEMA6C	CALU
SLC35A1	CLCN4
SPSB3	DNAJB2
TMEM86B	EXOC7
ZFAND2B	FLII
CYGB	HNRNPC
ARPC5	MEGF10
MKKS	PSMA1
MRPS34	PSMA2
PPM1F	PSMA5
ALCAM	RPLP0

RRAD
SLC1A3
SORL1
STAT5A
TUBB4B
CHRM4
EI24
EPHA4
FLRT3
ICAM2
NAT8
SERPIND1
SPRR1A
TAPBP
TRA2A
TRIM59
SLC10A1
SOX4
SPTBN4
TMEM54
ANKRD34A
ASB4
ATOH8
ATPAF1
B3GNTL1
B4GALNT4
CEP131
FAM193A
FAM219A
FCRLA
FRAT2
GALNT2
GJB6
GPRIN1
HPS4
LPAR6
LRRC58
MAD1L1
MARK4
MEGF8
MPRIP
PAQR6
PRR5
PSEN2
SECISBP2
SKA1
SLC35A3
SPG11
TCTN1
ZBTB41
ZMYND15
ADRM1
AKAP1
AOC1
ARPC2
CAPN10
CDKN3
CKMT2
DNAJA1
DNAJC5
ERLIN1
GJC1
HPX
HSPH1
IGHMBP2
JMJD6
KHSRP
LGALS3BP
LMAN2
LPO
NFU1
NME4
NPPC
PLD3
PSMB2
PSMC2
PSMC5
PSMD12
RBP3
RPL3
SEC61A1
SPINK1
SSR4
SUCLG2
TAX1BP1
TK2
TMSB4X
TPR
TRIM5
UBE3C
UPF1
VPS4A
ACTN3
ACTR3
ASIC3
ATAD1
BAG2
CLTCL1
COPB1
DCTN2
DNAJB6
DYNLL1

ZNF526
ACTG1
MT1A
PTK2B
STMN1
ACSS2
MAFG
CEL
EEF1B2
FBLIM1
FCER2A
KIAA0753
NUAK2
PPM1B
RTP4
TACC1
CCDC154
S100A7A
TMEM201
WDR38
ZNF121
MTIF2
AFG2A
ENTPD7
FGD1
FGD5
H1-3
IL21
KHDC4
ORAI2
PAGR1
PITPNM2
PLBD2
PPP4C
RNASE6
SFPQ
TMEM87B
TUBB1
WDR44
XCR1
ZBTB2
CD52
CENPK
CYP2S1
FGL1
FXYD5
HNRNPM
LGALS8
PDLIM2
USP5
IFFO2
EXOC4
GLE1
IMMT
IPO7
MIP
NTS
PDIA4
PDZK1
PPA1
PRG2
PSMD1
PSMD8
RPS12
SLC4A7
SRSF9
ABCF2
CAPZA2
COPE
COPG1
EHD1
FAF2
GOPC
HLA-F
IFRD1
ITIH2
LMOD3
LRBA
MYO1B
PSMB3
PSMD11
RPL34
RPLP1
SCRIB
SCTR
SRSF4
TRIP4
UGGT1
CCT8
CXCL3
DNAJB4
GGCT
ISCA2
BOLA3
DNAJA2
GRPEL1
KDELR1
MRI1
RAB14
RNH1
SAR1A
SCAMP2

SLC47A1
SPTAN1
TMEM150C
UTP18
KLF4
CFAP157
CBX4
CKAP2L
GCNT3
NR1D2
RABGAP1L
STEAP3
YPEL5
GCN1
HAS3
MAP3K9
SCAF1
VPS8
ZP3
ALG6
BMP8B
FYTTD1
IL34
PIP5K1C
RGS14
SYNE3
TBC1D9B
TMEM268
TRAPPC3
TSPYL4
XYLT2
CNNM3
GARNL3
BACE1
GAMT
GK
CD34
FOXN3
GPR150
METTL25B
MIR351
MORC1
MSANTD2
PLEKHF1
POLR2A
RAD23A
RUNX1T1
ZNF791
SQLE
HSD17B7
RGS4
STX6
SURF4
UNC45A
APEX2
COPG2
DUSP19
ESYT1
KLHL40
MX2
NHERF2
REG3A
AUP1
FLG2
IBA57
SPRR1B
DERL1
DERL2
HRNR
NOP53
PATJ
QARS1
SEC16A
SCT
SERPINB12
ANGPT2
TRPV1
F2R
TGFA
F2RL1
DLK1
SMAD2
LRP1
TRPA1
PDE5A
PPARD
MB
CCR7
CEACAM1
CHRNA7
SLC2A10
SFTPA2
SLC24A3
S100A12
GABPA
DBH
DNMT3B
EPAS1
ARG1
RB1
TLR1
DCN

SMOX
UBR3
FBXL5
GREB1L
IFI204
IPO8
MRPL40
MRPL47
NT5DC3
PLCH2
POGZ
PRX
PYGO2
ROBO3
SBF1
SPIB
TPRKB
TSSC4
VILL
ZFAT
CCT7
GNAI3
GRB14
PRKCQ
RCN1
TRPV6
UGT1A7
CYP1A
ICAM5
LARGE1
LRP3
MEAK7
AZIN1
CLTB
HLF
IFI35
IQGAP3
RPL27A
SF1
STARD13
CTTNBP2
DPH2
ENHO
FGF11
IFT25
IRAG1
JAML
KATNAL1
MICALL2
MRPS18A
NCBP2
ENO2
FOLH1
AMPD1
CDC6
CLEC7A
DUSP1
TRPC3
CISH
HMOX2
CDH23
HBEGF
CEACAM5
CTNND2
CTSE
LTC4S
NOX4
PON2
PRL
GSTA1
CCL4
FSTL1
SPINT1
VSIR
IL1RAPL2
ITLN1
SPDEF
PEDS1
MIR15B
MIR205
MIR424
MIR15A
CREBBP
ERBB4
KDR
FLT3
EPHB2
ITGB3
EIF2AK2
HSP90AB1
LYN
PTPN1
TNC
TRPC6
ITPR1
CHRNA2
COL1A2
IFIH1
MAPK9
PRKCE
CTSL
FOXA2

NCLN	PRKAA1
SAMD4B	SIRT3
SCAND1	VIPR1
SERAC1	WNT3A
TBKBP1	AGL
TMEM184B	ARG2
UBN1	MTHFD1
SLC7A1	PTPRD
CYP3A11	PTPRO
H2-M2	SOCS1
KIAA1191	TSPO
KLHL36	ADARB1
MIR328	ANK1
PIERCE2	C1QBP
TSNARE1	SOX9
UGT1A6	CXADR
WFDC12	HES1
ZNF211	PTGIS
CD101	SHMT1
CFAP20DC	SMAD7
ENTREP2	ALOX5AP
HMGN2	ANGPTL4
IGLV1	CD63
LHFPL6	CHRNA6
MR1	CHRND
NOG	FHIT
NPR2	GADD45A
NUCKS1	GGPS1
NUDT7	HRH2
RNF19B	HYAL2
SCIMP	ITGB5
TNFAIP1	KLF6
NCKAP5	PLCE1
RELN	SLC22A3
SLC25A45	TCN2
TXN1	CHRNG
ACAD8	FABP5
ACRBP	PDE4C
ARHGAP27	PDE9A
BEND5	SULT1A1
CADM3	SUV39H1
CD2	DUSP10
CLIP3	KANK1
COX11	TUSC3
HAUS1	ADARB2
HDDC3	BCL2A1
MAF1	GRP
MLST8	HNRNPH2
NUDT16L1	ITGA11
P2RY12	KLF2
PLCXD1	MAVS

REM2	RGS2
SF3B5	TRPC1
SH2D2A	BICD1
SIRT7	FGL2
SNIP1	IL19
TEX30	PRDM2
TMEFF1	S100A7
TTLL3	PCMT1
UTP3	ATP6V1D
YAF2	CDYL
CAVIN1	FOXA3
HCAR2	MFAP4
PRIM2	MYOM2
TCP11L2	CER1
UTRN	CIRBP
KMT2A	CYP2A13
NSL1	HERC5
CARD6	MYO1D
CLDN12	SVEP1
NIPAL3	BPIFA1
EMP1	CHRNA10
FECH	CRISPLD2
ARPP19	H4C16
MAPKAPK3	NID2
MID1IP1	ENOSF1
UCK2	PPP1R14C
APOL11B	CALN1
ATP8B2	SPATA13
CROCC2	TAF7
EIF4E	NAA80
NORAD	PDCD5
PCOTH	SNTG1
RASD1	VWA8
TUBA4A	IRAG2
ARHGAP4	CLEC5A
CSDC2	DEUP1
DENND2C	NREP
ELL	GNL3L
FREM2	CDRT4
HECTD2	LINC00472
INTS10	MIR7-3HG
KRR1	MIAT
MIR17HG	RMRP
MLKL	SOX2-OT
NDUFC1	MIR22
NINL	MIR378A
NIPAL1	MIR106A
OSTF1	MIR338
PAQR4	MIR484
PDXDC1	MIR542
PHF21A	MIR1275

PINX1
POLR1E
PRRT1
RDH12
RER1
RNFT2
TMEM263
TSC22D4
XPNPEP3
HBB-BS
LCE3D
LMNTD1
MIR149
SAMTOR
WFDC17
ZNF286A
CD55
DMAC2
EBF4
GGN
APRT
ASL
CA12
FAAH
TNIK
DPYSL2
CYP2B10
CACNA1E
CDKN2C
GALNS
IGFBP4
BDKRB1
ANKRD11
CBR4
DTYMK
DZIP3
EFNA4
GALNT14
GJA3
HASPIN
HM13
HMBOX1
HNRNPUL2
HYLS1
KCNIP2
KDELR3
LCT
MAB21L3
MARS2
MIA
MRPL51
MIR663A
MIR205HG
DUBR
CAMK2A
IDH1
JAK2
NFKB2
ACVR1
CDK6
DDR2
SYK
UGT1A1
BDNF
EDNRB
EGLN1
GRIA3
GRM1
MME
PCSK9
TNFRSF10B
GRIK2
MCL1
PPIB
SPARC
TLR8
ACTA2
ADCY1
CASP1
FST
IDE
IRAK1
LEF1
MAPK7
OAT
ODC1
PRMT1
TPO
TSHR
WNK1
ADAM9
ADCY6
ARHGDIA
EEF1A2
FBN1
GABRB1
HNRNPA2B1
HYAL1
IRF7
KCNJ6
LPAR1
MAPK13

NFE2L1	MAPKAPK2
NOXO1	NRP1
NTNG2	PRMT5
PDPR	PROM1
PGS1	RAB27A
SCYL1	SLC18A2
SEPSECS	SMAD6
SESN3	SPRY2
SLC19A3	SYT1
SPRY1	TPM3
SRP19	AK1
TMEM53	ALDH3A1
TMEM67	ANGPT1
TNNC1	CA9
TTC36	CASP14
UAP1L1	COL6A1
UNC5D	CRAT
MCM7	CRYAB
ZFAND2A	CYSLTR2
CACNA2D2	DDR1
LRATD2	GRM7
KCNJ15	GSS
NFKBID	HADHB
RNF152	IGFBP7
NPY1R	ITGAX
CLMN	ITPR3
KIF18A	NME2
LRRC59	PBRM1
ALG14	PSAT1
BRD1	RPS6KA2
CCDC158	RPS6KA5
CSNK1G3	SIRPA
FES	SKP2
HSPA14	TACR1
LRTOMT	TALDO1
MMACHC	TRAF2
MTSS2	UCP2
MYF6	UQCRC2
REPIN1	VAMP2
RTTN	YES1
SEH1L	YWHAQ
ACOT8	ADH5
CYRIA	AGRN
ERFE	ANO1
IL4I1	CD74
KCTD7	ENPP2
KLK8	KCNE1
MAN2B2	LAMA1
NACC1	MAF
NPTXR	MMP16
PSPN	NEDD4L

SEPTIN5	PAX3
SEZ6L2	PEPD
TSNAXIP1	PTPA
TSTD1	RIPK2
ADAM11	SERPINF1
TRPV3	TRPM8
CRIP1	ABCC9
FCGR3A	ACAD9
MAP4	ADCY8
PCSK5	ADCYAP1R1
RPS7	ATF3
CAMK2N1	BTRC
IFITM2	CCKBR
MYH1	CFLAR
RANGAP1	CHD7
RRS1	CKB
SIPA1L3	CRHR1
SLC25A5	DCK
SLCO1B3	DKK1
TEF	DLG1
ACOT1	FBLN1
CD6	FGF9
CLDN15	FOSL1
COL2A1	GPR37
DAPK1	HPSE
EIF2B5	ISL1
ERLIN2	KAT7
GGT5	LAMP2
GOLPH3	LDHB
GUK1	MAN2B1
H2-AA	MAOB
HOXB6	MBP
ILVBL	MEF2D
IP6K1	NOX1
KLHDC8B	NPY
MGAM	NRF1
MRTFB	ORAI1
NLK	PLTP
PIR	S100A4
RAE1	SET
RHOV	SMAD1
RPL7L1	TCF12
TDRP	TNFRSF21
TOM1L2	TRPM7
TSPYL2	TXNRD1
TUBB2A	USP14
WASF1	VIPR2
RPR	ABCG1
CACNA1H	AKR1C1
GSK3A	APOD
IVD	BST1

PLIN3	CCL7
SLIT3	CMA1
TKFC	DGKA
HTATIP2	DPP10
C19ORF12	DYRK2
CFAP210	FABP7
CLEC1A	HNRNPD
DUSP28	LRP8
H2-Q7	MFGE8
SCNN1D	NOD1
SERPINA3G	PCDH19
SLC24A5	PHEX
TMEM238	PIK3R3
TREML1	PPM1A
ZNF114	RAN
ZNF33B	RIMS1
ZNF516	SEMA7A
TMUB2	SNAI2
ARHGAP8	SOCS2
ATP6V1C2	SRF
BCAS1	TCP1
BLK	TLL1
DOCK2	TRAF3IP2
FRK	TRPC4
GNL2	USP10
ITGBL1	ADGRG1
MORC3	AKR1B10
MRPS12	ATP12A
P2RY14	AUH
RNF26	CITED2
ROPN1L	DDIT4
SCN7A	FERMT3
SIK3	FOSL2
SUGP2	FYB1
TAF1	HAS2
TBC1D17	KCNH7
TMEM156	MT2A
WNT10A	NR4A3
WSCD1	PTPN3
LCE3E	ROBO2
NBPF9	SLC16A7
APLP2	TAC3
IMPA2	TACR2
DISC1	TICAM1
BHLHA15	AKAP9
HADH	ATXN7
TCIRG1	BCAP31
PHF1	CCL21
IGKV8-30	CD69
AIFM3	CDK20
CES1F	CHRM5

DARS2	DFFB
DONSON	ELAC2
GPR55	ERO1A
IFT70B	G3BP1
KLK6	HS6ST1
MPV17L	IL1RL2
MROH1	KRIT1
POLR1G	MAGI2
SKIC8	MAP1LC3B
SLC50A1	MCC
SNX3	METTL3
TFB2M	PARD3
TMEM107	QKI
TMEM204	RAB1A
TMEM44	RGS10
TMEM88	RGS9
WDR18	RXRG
ACBD5	S100A6
TCEA2	SEMA3F
ARAP3	SLC44A1
EPB41L4A	TIA1
NPEPL1	TNFRSF12A
PLIN2	TRAF5
ABHD4	WNT5B
COL14A1	ARF4
DIAPH3	ARHGAP5
FOXQ1	CCL19
GCHFR	CD160
IFI6	CD68
KPNB1	CDH6
ORC6	CHIA
UMPS	CLCA2
CACNG1	CLDN2
CIAO3	CNN1
CST8	CXCL13
GARRE1	DKK2
MC5R	DOCK4
OARD1	DUSP4
OTOP1	EFHC1
SMG8	EGR3
SPOUT1	ELMO1
TMEM221	EREG
UMODL1	GABARAPL1
USHBP1	ITGAE
GPAM	IVNS1ABP
AQP2	JAM2
APBB1	KLK5
CAMSAP1	KLRK1
CHCHD2	LGR6
CLEC4D	MTHFD1L
CLIC6	PPP1R15A

CLPB
CPNE4
FAM171A1
FRMD8
FZR1
H2-AB1
HEATR3
PLEKHB2
RDM1
ROR2
SAP30BP
SASH3
SFT2D2
SH3RF2
TEC
VASH1
ZNF395
ABCB1A
ARID5B
ENTPD5
MT1G
NR1H2
SOX17
SHISA7
CYBRD1
SRD5A1
TRMT1
EEIG1
AEN
B9D1
CERS5
CLPP
ERI2
FAM171B
GABRE
GPR68
KIRREL3
MARCHF2
MEPCE
NCOA6
NPHP1
PHF10
RNF157
RNF39
SDSL
SNRNP25
TTPAL
UBE2D2
ECH1
FABP3
MIR425
PRDX4
RAB3A
SEPTIN9
SERPINB8
SGO1
SH3GL2
ST8SIA2
TET3
TFF2
TNFSF14
TSPAN5
XK
ZBTB17
AGR2
CD93
DCDC2
DHX15
HAS1
HAT1
HDGF
HSF2
IFNA1
LPCAT2
LRIG1
LSR
MGAT5
MSMB
MYCL
PCDH8
PCDH9
PDE4DIP
PROZ
RAB5C
S100A1
SERPINB9
SRSF6
STIM2
SYNE2
TSC22D3
TSN
WLS
AKAP12
AKAP5
CREB5
ERP44
FOXJ1
NOS1AP
OXA1L
PLPP3
RARRES2
S100P

ACTR8	SERINC1
ATP6V0E2	SLC52A2
AXIN1	AK7
CACNB3	ANGPTL1
CIPC	ANKRD2
NEPRO	CCL24
SPRR2A	CD5L
ZNF580	CEACAM7
ACSS3	ERRFI1
CYB5RL	ESM1
GPR173	FLRT2
JMJD7	LILRB4
KCNJ12	LZTS1
PLCG2	MAST2
PPP2R3A	MMP25
PRRT4	NAT10
PSIP1	NFYC
RYR2	TEKT3
SLX4IP	TET1
SULF1	THSD7A
TP53INP1	TLR10
ATP1B1	TRAPPC10
MTTP	WDR11
CCDC190	WWTR1
PER3	ABCA13
ARL15	CCNL1
CEP128	CIP2A
CLPTM1	CXCL6
FAM117A	DCD
FAM13C	EYA2
HCN4	FN3K
HES2	GNPDA2
HOXB3	GPRC5B
SFXN5	IL25
SH3BGR	MGAT5B
SWAP70	NECTIN3
ARCN1	NKRF
ARFGAP1	NLRC5
CCDC77	NUDT6
DNMT3L	OLFM4
FAM210A	OSCAR
GALE	PMEPA1
IGSF10	RAB26
IRX4	RGS11
NRXN1	RHOD
PFKFB4	SGMS2
SLC38A4	SLC45A3
SMAGP	TENM3
TFDP1	TES
TM4SF1	ZFP36
TSC22D2	ZNF143

ADGRL3	CDC42EP3
IGK	CEMIP
UBE2D2A	CLSTN2
PRKAR2B	EDIL3
BIRC6	EIF2A
GTF2F1	GEMIN5
NIP7	HOPX
IDS	INSIG2
ADCY4	ISYNA1
ARHGEF10	MOB3B
ATP6V1G1	OMA1
B4GALT2	PDE8A
BCL11B	SERPINB4
CD84	ADGRL4
CLASP1	ARB2A
CRADD	CDHR3
DDX54	COBL
DSN1	DDT
FHL3	MAFK
GFUS	MAPK8IP2
GIT2	MOXD1
H4C9	MPP7
LINGO1	MRPL28
LRP12	MYO16
MAN2A2	NUDT12
NMRAL1	PAMR1
PGLYRP1	PPHLN1
RND2	PRUNE2
RUSC2	PTGR1
SBSN	SOBP
SCN3A	SPCS3
SGTB	SRXN1
SNHG11	WHRN
SYNGAP1	AGBL4
SYT17	AMOTL1
TRIM29	ANXA10
XPO4	ATP10B
KLHL35	CD24
ASTL	CPXM2
CD207	ERP29
CDH26	EXPH5
DND1	FOCAD
EEF1AKMT3	GZMK
FAM174C	HSPA12A
INSIG1	KLRC2
ITPRIPL1	NLRP10
TRMT10B	RCN3
KNSTRN	TACC2
ALDOC	TENM2
F5	ZNF277
FTL	ALG10

PTPRM
CDH4
CSF2RA
NRIP2
ARRDC4
CDC14A
CMTM6
CREG1
ECM1
HRG
MYRIP
PRKAG1
SEMA3G
SP7
UNC5C
VKORC1L1
ADAMTS12
MAFA
NOP58
CES2
MIR23C
TPP1
CAMK1
CCDC122
CEP20
COQ7
DZANK1
EMCN
FAM167B
FAM168B
FOXL1
GIPC3
KLRC1
PDE8B
PIGB
PIWIL4
PROSER3
SLC26A10
TBC1D25
ZNF627
ANKFY1
ARAP1
ARHGAP15
BYSL
CDH22
CPED1
DENND5A
FYCO1
KLC4
MFAP3
MROH6
ANKRD44
ARL4A
CFAP58
CPO
CST1
DNAH6
FBXL7
GMPR2
GPSM1
KLF9
KYAT3
M1AP
POLR2H
RCSD1
TENT5C
VEPH1
ZFYVE28
BTG3
CLEC9A
GIMAP2
SLC38A6
TMED3
ANKRD7
ARMCX2
FBXO33
PRSS35
ABHD12B
DIPK2A
H3C14
MSANTD3
MTUS2
OSGIN1
PRRC2B
SLC5A12
SREK1
TCIM
ARRDC1
HNRNPLL
MARCHF1
NT5C3B
CYYR1
FAM120B
RAP2C
TSPAN17
CRACD
TMEM182
TMEM196
ARPIN
MIS18BP1
DEFB103B
BRD10

MRPL20
MT1M
PALM
PLVAP
PYROXD2
RASAL1
SEMA5B
SMIM15
ZC3H4
ZHX3
ELAVL1
INPP5D
LY6E
PSME1
SLC25A25
TDO2
TSHB
PRC1
NRXN3
TCERG1
SDC3
BCLAF1
ESD
OPTN
SHCBP1
AKAP8
B4GALNT1
CORO2B
DDX23
DNAJC7
GNA11
KLRA8
LDB3
LY6A
NLGN3
NT5C2
NUP160
PDXP
RHBDD2
SAC3D1
SH2D5
SIX4
SMURF1
TMCC2
TSPAN2
VWA1
KIR3DL2
ZSWIM2
BCO1
POU2F2
C1ORF43
DLEU1
MAP10
ZBED5
CCDC71L
SLC35F4
MIR214
XIST
DLEU2
MIR152
MIR200C
MIR326
MIR93
MIR96
MIR107
MIR122
MIR135B
MIR193B
MIR342
MIR34B
MIR449A
MIR9-1
MIR18A
MIR30A
MIR33A
MIR28
MIR29C
MIR381
MIR1247
MIR138-2
RN7SL1
SNHG16
LINC01619
MIR744
MIR1908
MIR449C
MIR937
FAM230B
MIR4732
RNU6-1
MIR301B
TMEM72-AS1
LINC00987
ANXA2P3
BLM
IL1RN
SLC11A1
HCK
GLB1
CD151
ADAMTSL1
FRMD4A

DEPTOR
FAM50B
IGHA
PLA2G4F
SDHAF4
SHISA9
TAF4
TCTN3
TSPAN10
UQCC5
ZNF264
RFX1
TRIM65
BRD8
CALCRL
CCNT2
CHRNE
CIAPIN1
COL4A4
DGCR8
EEIG2
GIGYF2
MRPL13
MYOCD
NEGR1
TIMM13
WDR75
XPO5
CYTIP
IKBIP
MDH1
CLSTN1
GNA15
HNRNPDL
LAMC1
VEGFD
BCL2L13
CXCR5
GMDS
GOT2
PITPNB
ABHD12
ACE-1
CILP
EIF4G3
FOXP4
H3F3B
LOXL4
PADI4
PCLO
PCNX1
CYS1
CRACR2B
ABCA3

PTPN14
RAB2A
SNU13
MYBPHL
A3GALT2
C2CD4C
CD300LB
CHP2
H2AC13
MS4A4A
MYO15A
NDUFAF8
OTUD3
RMND5B
SERPINA3C
SLC24A1
SRMS
ZNF721
PCOLCE2
ATP2C1
CDK8
CETN2
IRAK4
SAPCD2
C1GALT1C1
EIF5A2
GRIP1
GTF2H5
HARS1
MCFD2
MSRB1
NAA10
OAS1A
REXO2
SOWAHC
SPI1
TBC1D5
AKT3
CAV2
SLC2A12
SLCO4C1
TTC7B
COPB2
EBI3
PDGFA
SYNGR1
BGN
HEY1
NASP
TRAP1
ANKRD33B

C11ORF68
C17ORF67
CD79B
COX6B2
CTF1
DNAJC15
ESRP1
GBA1
HUNK
MBD3
NEO1
NOL6
PARD6B
PJA2
POC1A
RPL23A
SASH1
SCAMP5
SPART
SPCS2
SPNS2
TCEAL9
TXNRD3
VPS29
WFDC13
CAPS
FOLR3
ANKRD42
ANKRD54
BTBD6
FBXW10
HRCT1
KRT78
MTCP1
NLRP6
RAB40C
RINT1
ABCB1B
GOLGA8N
PROC
RPL13
SLC6A2
ALYREF
UBFD1
AHCYL2
ARID3A
C2CD2L
CCBE1
CENPN
CHST2
IGSF9

ITGB3BP
KBTBD8
LRP10
MYL7
NAPEPLD
NEURL3
NPHP4
OSGIN2
PLEKHO1
SETD5
SH3D19
TRIM36
USP13
MFSD2A
TRIB3
ALS2
THOP1
CA2
TRH
MFF
SRM
ARID4A
ATXN1L
CORO7
COX7A1
CYP2D22
DENND6B
DTX2
FGFRL1
GAL3ST1
HAP1
HPF1
LEFTY2
MIR100
NDUFA13
NNAT
NT5M
OGFR
PANX1
PLXDC1
RNF208
S1PR4
TFB1M
UFM1
UPF3B
ZBTB6
EHD2
KCTD15
RHOB
INF2
NR2F2

SORT1
EPS15
FASTK
FIGN
SCAPER
SPG7
UACA
WWC2
ARAF
ASB2
BEX3
CENPV
CMKLR1
CPEB3
EIF1AX
EZH1
HIRIP3
MCCC1
NDUFB3
PDZK1IP1
SLC15A3
SLC16A14
SLC25A23
STARD5
CROT
F7
MYLIP
TCAF2
BIRC5
ASB16
FHIP2B
INTS11
KLRF1
KNG2
SMIM24
ZNG1C
ARMC5
EID2B
FRMPD1
HSD17B10
INTS5
LRRC73
PCLAF
PLET1
RAD51AP1
RBM44
STPG1
GSTA3
ACBD4
AP2B1
CHCHD3

DDOST
DDX10
DGKH
ECPAS
ELOC
GMPPB
MARVELD1
MXRA7
RNF24
SCRN1
SDAD1
SPA17
ST3GAL2
STX11
TRANK1
SKN-1
FBXO5
RPL12
BIN1
RBL2
IL20
ALPK1
ANAPC1
ARFGAP3
BCKDK
FLVCR2
GSE1
KCNQ1DN
KIDINS220
MECR
NDUFA7
OTUD1
PIM2
PITPNM1
SERP1
SLC26A6
TLE2
TMEFF2
TRAIP
AHNAK2
CGREF1
PAWR
TINAGL1
TNFRSF19
DCP1B
H2AC15
ABCA9
ERICH5
FAM131B
H2BC18
SPACA6

TMEM234
TRMT44
ZNF598
SPAG5
DNPH1
AIF1L
ARSG
BMP2K
CD302
CDC42BPB
GNAZ
HNRNPH3
HOXB9
KIF21B
MBNL3
MPC1
NKTR
PUS7
TBCD
LEMD1
ANKRD36B
DUT
FBXO27
FOXP2
GRIN1
RCAN2
ADPGK
APOBEC3B
BAZ2B
CDCA7L
COQ3
CYB561
ESPN
FCGRT
GAD1
GPR146
HSD17B8
IRX3
LRRC8C
NDUFB2
ODF2
PCK2
POP1
SH3BP2
SLA
SLC66A3
SMARCD2
SYT7
TBC1D1
TCF21
THYN1

WDR3
ZC3H6
CMC2
CYB5D1
FSTL5
GAB3
GP2
KBTBD6
KIF27
MON1B
MYZAP
PARD6G
SCAMP3
SIM1
TVP23B
BIN3
CITED4
CRTC1
DNLZ
DYNLRB2
FBXO43
HECTD3
MCTP1
MED25
MLLT1
PNMT
TSR2
VPS37C
ZBTB47
LCE3A
CD55B
MIR667
RNASE7
TNIP3
CASP4
GNB2
TIAM1
CTDSP1
CXCL15
FNDC1
FSTL3
ITPRIP
MBOAT1
MST1
MXRA8
NIN
PARP12
PCDH1
PLLP
WDR43
WNT10B

MATN2
CAMK2D
UGCG
PLIN4
CA13
LRRC23
MIR25
MYT1L
POLDIP3
RADX
RBM14
ATG101
BEX2
CACNG8
CBY1
CFHR1
CUX2
DLX2
FBXL18
H2-DMB1
IZUMO4
JSRP1
KLHL5
MED22
NAA60
NALF2
NSG1
P2RY13
P4HA3
PCDH18
PEG3
POU6F1
PRDM6
REEP1
RUBCN
RWDD1
SEC31B
SEPTIN11
SLC35A2
SNX15
TAF1B
TRIM7
CIART
CKAP2
EMILIN1
EMILIN2
MKLN1
PACSIN2
SCN1B
TBC1D15
TMOD2

TST
SEMA5A
GRAMD2A
KIR2DS2
ST14
TUBB5
ATP5ME
BPTF
CELSR3
CTTNBP2NL
EDN3
EVI2A
GRAMD1B
IL10RB
INPPL1
INSL3
KMT2C
OGFRL1
PKIB
SIPA1L1
TBC1D31
TIMM10B
TRIOBP
XPNPEP2
FEZ1
MIR3107
NR1I3
CES2G
PTK6
SUPT6H
ZBTB45
ASPHD1
CUL9
DNASE1L2
FAM181B
GIPR
IFT56
INTS1
KATNIP
MFSD13A
MTMR12
NVL
POC5
RGP1
RPL22
RPP14
SERPINB10
SYT15
TADA3
TNFAIP8L2
TSPAN32

WFIKKN1
ZNF655
KCNJ2
MZB1
KRBA1
SNHG15
AP1G1
CENPI
COMMD1
COX6A1
DROSHA
EHD3
FLRT1
GHITM
HERC6
JADE3
PARM1
RASSF2
SLC25A22
TSPAN12
UBAP2L
CALM4
ZNF487
MMD
LAMP3
MYADM
NRIP1
PC
TUBA1C
APLP1
DERL3
DLGAP4
DNAJC9
ENAH
EP400
FNBP1L
LYPLA1
MAP2K7
PLOD3
SYNDIG1
TRAM1
USP36
CLEC4A1
EIF6
ABCD3
ADGRE4
C1ORF116
CARS1
CCDC17
CIDEA
CPE

CRYBB3
DCTN3
FRA10AC1
FUT7
HCP5
IFI203
LRRC56
MEP1A
NANOG
NAXD
NME5
QRICH1
SAMD12
SWI5
TRARG1
GPX-5
IRAK3
ALDH3B1
ELK4
FABP6
LARP1
MIS18A
MPP1
NUP155
PDHB
PSME2
RPL9
RTN1
TAF15
TAF1D
TIGAR
ZFAND5
GST-4
PLP1
ACSL3
CYP4A14
ECT2
OVAL
SLC5A5
ACBD3
ACER2
AP2M1
BLTP1
CD7
CEP162
CST6
DARS1
EIF4G2
FASTKD5
FIBIN
GRAMD2B

MARCHF4
NSFL1C
NSUN7
NUMB
OLFML2B
PRRG4
PTPRN2
RCC1L
SLC2A6
SNRK
STAG2
TMEM176A
TRMU
CHRDL2
CIAO2A
GPR108
GTF3C6
GTPBP8
KMT5C
LMBR1L
LRCH4
NDOR1
NTF4
PERM1
RASAL3
RTL8A
TMEM18
DNAJC3
PTPRF
SERPINA3N
SLC5A4B
TIGD1
ZNF594
ZNF707
ADAR
CHD3
CSPG4B
GPRC6A
HACD3
IER5L
IRF5
MND1
NACC2
SCARNA2
SLC25A33
SUCLA2
ZFP319
ZNF771
ZNF813
CSNK1D
DYSF

HTRA3
RNF144B
RNF139
MIR375
CCDC30
COQ4
EIF3A
RANBP3
UBA7
ATCAY
ATXN7L2
BRF1
CEP89
DXO
GDPD2
GPN1
INTS6L
KIF6
MRPL27
NHERF4
NUDT8
PARD6A
PODN
PPP4R3A
RBM42
SH3BP5L
SLC7A6OS
STARD3
TARBP1
TMEM104
TMEM115
TRPT1
USP19
VPS28
WFDC3
ATP6V0D1
GLG1
HJURP
SRR
TNRC6B
TYRP1
FOXC2
GRHL1
LGALS2
PGM2
RAMP2
SORCS2
TPBG
UCHL3
NCOR1
COA8

TRO
CDK14
DCX
AGTR1A
B4GALT1
BPGM
CREB3L2
MT1X
PCCB
PRPS1
TECR
TMEM45B
TMEM8B
LFNG
RAP1GAP
CEP120
CHST6
CNIH1
CYB561D1
ERLEC1
FAM32A
HOXB4
KIAA1671
KLHDC9
LIN9
NCR1
PARP10
PCDH12
RAB36
RANBP17
SSH3
TFPT
TRIT1
ZNF93
ZSWIM4
ALDH6A1
ALDH7A1
C7
DCTPP1
DPP7
DPT
GPR183
IGTP
RIPOR2
SRP9
WNT7B
PHLDA3
TRIM3
ASCL3
HBB-BT
SNORA73B

TFAP2A
ZNF320
GARIN1A
GPX7
HEATR4
MS4A4B
PYURF
SLC22A14
ARHGAP29
PRSS12
TANK
TCF3
BCKDHB
DHODH
DPEP1
EIPR1
GJA4
GPATCH4
HS1BP3
LUC7L3
PDK3
PLEKHB1
ANAPC7
CNP
DCUN1D5
DHX35
KATNB1
KPNA5
MAST1
MED26
NOL12
NR6A1
PLEKHH3
POLR2M
PSMG3
RBIS
RPE
RPRD2
SCML2
SLC25A17
TFIP11
TMEM138
TXNDC11
TXNDC12
TXNL4A
WDFY2
WDR37
YIPF3
ZNF532
EPHA10
ACSS1

MIR6722
SPDYE8
ATG3
CARMIL1
CBX7
EEA1
GPX8
LONRF3
RPL22L1
CAMK2B
ARMC6
BCL7B
ENKUR
GPR107
GUF1
H2BC11
HRH4
HSH2D
LRRC2
MFSD8
MRPS10
PNMA2
RANGRF
RGS18
TIMM8A
TMEM168
TRUB2
UPRT
ZDHHC7
CEP43
DAPK3
DCTN5
EPB41L2
FERMT1
GRM5
GTPBP4
HEATR1
HSPB6
NAA40
NKD1
PAM16
PIGL
POU2F1
RADIL
RNPEPL1
RPUSD3
SH3BGRL3
SHISAL1
SNX21
ACO2
ACTN2

ADAMTS4
ANXA7
COL16A1
PIK3IP1
TTK
FAM234B
CRIP2
IGKV6-15
DNAJC9-AS1
GNMT
PLCB4
UPP1
ACAA1
BLNK
C3ORF33
CDV3
COX6A2
DPYSL4
LBHD1
PRPS2
SAMD4A
SRI
ZNF316
MS4A8A
PCDHGB2
ARC
CR2
GVIN1
HSP-60
IFT46
TAT
VPS72
ANKRD27
ATG9A
CNFN
CNOT8
FBXO36
FOXN4
GET4
H2BC6
LCLAT1
MEX3D
MTMR14
MYBPC2
NFRKB
POU2F3
RPL36AL
SCARF1
SEC14L3
SERPINB3
SGSH

SVIP
SWT1
TEX9
TTC19
ZBTB48
CACNA1G
LRRK2
CA8
ZFYVE9
ARHGAP24
DEPDC1
EEPD1
MBNL1
MT1H
MYL6
NXF1
PCYT2
RGL1
RSL1D1
ST3GAL6
NCOA7
LGALS9
MDM4
TRIM24
CRB3
FBP2
NUMA1
PRELID3B
REEP5
AMBRA1
ATG4C
CFAP45
CHST12
CHST4
CLEC4A3
FAM153B
FAM3B
GCFC2
GNGT2
GTPBP3
GUCD1
KCNQ4
L3MBTL2
LRWD1
MED15
MRPL48
NAP1L3
OSGEP
PARVG
PIGF
PIP4P1

PLPPR4
PPCDC
RNF10
RRP8
SLC38A9
SLC9A9
SPATA2
SPATA2L
STX5
SUDS3
TAS1R3
TBC1D10B
TMCO3
UCN
ZGPAT
ZNRF2
CYP7A1
GPD1
MIR674
ATOX1
FAM111A
HNRNPL
OGN
PPP2R5C
PTPN2
RIOK3
SLC41A2
THBS4
FADS1
MIR202
CD1A
DEFB4
CNGA2
FCRL1
PCDHGA5
TMC3
TSSK4
ACTG2
C14ORF119
INKA2
SERF2
AMIGO1
AVL9
BRINP2
CDR2L
CHCHD1
CHODL
CLDN9
CLK2
DCSTAMP
DCTN6

DLK2
EFCAB7
ENTREP3
FAM174A
GHDC
IFI44L
IL17RC
IQCK
KCNF1
KLHL26
LYZ1
MRPS35
MTCL2
MYL6B
NBEA
OSCP1
PCDHGC3
PCGF2
PCID2
PIP4K2C
RBAK
RNF31
SELENOH
SIX5
SLC37A3
SNRNP35
TLN2
WDSUB1
ZFAND4
ZMIZ1
ADAMTS9
SCPEP1
SH3KBP1
SOX18
NCEH1
RNF181
CYP4X1
NR0B1
OSM
ABCB6
ATP1B3
CCN5
CD200
FLG
GCNT2
NRGN
PBX1
PCP4L1
PEA15
RASA1
RPL10

SFXN2
SLC46A1
SLCO3A1
SND1
TPD52L1
SC5D
ARL6IP4
CHST7
DNAJC8
ELP5
PIGU
SNRPC
TESK1
WDR45B
AFF2
BAIAP3
CHIC1
CMTM4
HCCS
IGSF8
MB21D2
NEUROG1
PRRT2
RIPOR1
STRADB
TBC1D23
TRAPPC1
ZBTB46
ZC3H18
BMI1
CENPW
HMGB3
IGF2BP3
KCNK1
LGR5
PPAT
RAPH1
SEL1L3
TRPV2
ARSI
CEP19
NDUFAF2
SRRM4
ACAT1
SPIN2B
MIR151
PCDHGA9
PNPLA6
ADAM5
ATP1A3
KRTAP17-1

STBD1
DACT3
DNM1
FAM120AOS
GIMAP6
KLK9
KLRB1C
KRT77
LPIN2
LRRC14B
PNN
PPP1R14B
RNU12
RPTOR
TFDP2
UBE2U
ZDHHC2
ZFP652
ADPRHL1
BTBD7
CASQ1
CDK5RAP1
COX19
DUSP13B
ISM1
KIF26A
MAK16
MAP3K7CL
MRPL34
MTMR10
MYDGF
PFDN1
SPIRE2
SYT5
TAGAP
TBX6
TMEM59
ZNF829
ACSL1
PAIP2
SMAP2
RFXANK
MANF
SOX12
ZBTB20
KNDC1
ACBD7
FETUB
H2AZ1
INHA
MIR3654

SEC31A
SRSF3
CCL9
ABCC5
SLCO1A1
AURKC
KCNA3
ALKBH7
ALKBH8
CUEDC1
DUSP6
FLAD1
KCTD17
LRRN4
POMT1
RNF167
ST7L
TAF5
ZNF644
ACOT7
AGAP3
B3GALT2
COX10
DDN
DHX38
EFHD1
F2RL3
FUBP3
HDGFL3
MZF1
PDGFC
PIN4
STK19
TCEA3
THSD1
ZNF703
ZNRD2
DOHH
SERPINB7
MXRA5
CDKN2D
PSMB7
RPS3A
SLC25A1
LRRC4C
SLX1A-SULT1A3
TMEM14B
ANKFN1
C5ORF15
CEBPZOS
CRYGC

TRIM30A
TUBB
ZNF684
ZNG1E
B3GAT2
B4GALT4
CCNB1IP1
CLCA3
DPF2
DYM
GPIHBP1
PCDHGB1
PRICKLE4
SLC6A20A
ADGRB1
ARHGEF11
CAMK1G
CNNM4
DCUN1D3
DR1
ELMO3
FCRL2
GCC1
GET3
GRIN3B
GSN-AS1
HEATR5A
KCNC1
MAGEH1
NRG4
PRKRIP1
PRPF18
RPGRIP1
SAMD5
SDHAP1
SLC35G5
SPOCD1
SPOP
TAB3
TMEM71
TPCN2
TTLL5
UCK1
VBP1
WASHC5
CHIA1
H2AC24
PACS2
RPL34-PS1
ZHX1-C8ORF76
ECI2

FNBP1
MXI1
RBBP6
YBX1
CCNI2
ESPL1
LYPD8
MGP
NEDD9
ATP6
PENK
RAB3B
GFRA1
GPR65
GRIN3A
MACC1
NEIL2
RNLS
ZRSR2
ABI3
AJAP1
AP1G2
CCDC28B
CD180
COL23A1
CPNE7
DOC2B
GAN
ITGA10
MRAP2
NHSL3
ODAD4
OGFOD1
PCSK4
PHF12
SMYD4
TMEM256
TNIP2
ZBTB26
ZDBF2
GNL3
HACL1
PAFAH1B1
PAOX
SLFN1
ST6GALNAC5
ASB9
CCDC82
YY1AP1
ALG13
CES3B

CYTH3
DET1
LCE1E
MIF4GD
NOXA1
PATZ1
PKIG
PKP3
RIMKLB
SLC38A7
SPRR2E
TADA2A
THAP11
ZFP39
ZNF414
ARHGAP30
ARHGAP6
ATP13A1
BAHCC1
CEP164
DNAJB13
ELL2
EXTL3
FAM135A
FEM1B
GIMAP8
H3C2
LNPEP
LRAT
NAA20
NCAN
PLEKHG2
PPP1R3C
PRRG1
RASIP1
RSRC2
SCRN2
SNRPB2
UTP11
RIMBP2
CYP8B1
GLI2
HMSD
CHI3L3
EBF1
MYO10
PPP1CA
RPL7
TTTY3
UBE2L6
UNC13A

ACER3
ANKRD29
DMC1
GLDN
LIN28A
MRPL11
PACC1
PHYHIP
RAVER1
RBKS
SLC25A39
SLCO5A1
TICAM2
CMPK1
MAP1S
SMNDC1
STX8
NOMO1
PHF2
TMEM38A
BAAT
CRK
FKBP11
LAMB2
POGLUT1
PPP2R1A
SDCBP
SEL1L
SORD
STC2
GRID1
GNB3
GDNF
SHOX2
CFAP184
CLEC4F
CYP4F3
EEF1D
GPR141
LRRIQ1
MGAT3
MPIG6B
MPST
MYO1G
NEDD1
NGEF
OLFM1
PFN4
RAI2
SERBP1
VSIG8

ZFP94
ZXDC
CARS2
CD300A
CEP95
DCAF7
DCLK2
DHPS
DNPEP
DPP8
FICD
GON4L
HIKESHI
HVCN1
IKZF3
LRRCC1
NBEAL2
NCAPH2
NDRG2
OTX1
PCDH20
PIGN
PTTG1IP
TRIM54
UBLCP1
USP39
ZNF511
ARHGEF5
HOXB2
IGSF9B
MIR2861
PLCH1
SCUBE1
C1R
ENPEP
PTMS
SQOR
NES
UBB
FAM46B
DDX52
LMBR1
NFE2L2A
A4GALT
ACTR1A
ALG8
DCAF13
DOC2A
EIF2B2
GIMAP4
GPR35

HOXA9
KCNJ10
KIF1C
KIF7
LRRC15
PKD1L2
POLR3D
RABGGTA
RBM22
RRAGA
SCUBE3
SDCCAG8
TMEM184C
TMEM40
TONSL
TTBK2
TTC14
VPS33B
ZNF69
AACS
ARHGAP11A
CBLB
FDX1
GPCPD1
PRKCH
VPS18
ZBTB5
ISLR2
SGSM2
LAMA3
LINC02688
C15ORF48
CCT5
EPG5
NCOA4
CFI
CRYL1
CRYM
FARP1
MYO19
ORC1
PKP2
RARRES1
ROBO1
TREX2
DGKE
H2-T24
PLA2G2C
BSDC1
CD300C2
CEBPE

CHPF
COG4
CYTH1
EAR2
GPR18
IAH1
MTURN
MUC13
OAS1F
OR8B1D
ORMDL2
PPP1R13B
PRORSD1
RSPO1
SERPINB9B
SPATA18
TXNDC17
ZNF555
ABCA4
ADAT2
AKAP17A
B3GNT7
CEACAM13
EPN1
MIR3613
NPFF
PPP2R2D
RALGPS1
SERPINA3M
SLC15A4
SOD-4
TBC1D2B
COX1
ERN1
ACTC1
CLOCK
ITK
KDM3A
LRRC1
NDUFS4
PGM3
SLC25A37
VGF
TYMP
VPS41
LZTS2
SYP
RASEF
DYRK1B
ECM2
HAL

IL12RB1
STAM2
VPS33A
ABCG4
AK3
ARHGEF40
BRPF3
CAR14
CCDC25
CHAF1B
EOGT
FBLN2
LDB2
LDHC
NINJ2
RNF7
RRAGD
SAA4
SIAH1
TLCD2
TMEM131
TMEM165
USP4
C1RL
COPS3
DPF1
EID3
GIPC1
LRRC47
MYH2
PLCL1
RNF44
CD5
LINC03040
MYRF
NDUFA11
CACNA1A
CCDC85B
CYB5B
ESCO2
FAM78A
MOK
NTNG1
PHF5A
RHEB
TMSB10
PSMD9
CAPNS2
PACSIN3
TLCD3B
ERICH2

FBXW10B
GOLGA8B
VLDLR
ZNF134
ZNF138
ABCA12
ABITRAM
CHCHD7
CPT1C
DYNC1LI1
FGFR1OP2
GGTA1
H4C6
IRAK1BP1
LY6F
MAGI3
MED10
MRPS14
MUP3
NECAB2
PPFIA1
PRR36
RAB3IL1
SAFB2
SLCO1A2
SNX7
SOCS6
SYNC
TBATA
VNN2
ZNF460
ZNF775
ARK2C
CHL1
GBF1
GLT8D1
HCRTR1
RASSF6
TNN
UBR1
C1QC
EPS8
MLF2
POLR2F
PTPRH
MRPS30
MS4A7
AGTRAP
FIP1L1
IFT74
PPP4R3B

RIPPLY3
CDK10
CLDND1
CLN5
DOK7
DPH3
EPHA8
GBP5
KAZALD1
MED21
MYO1F
NDEL1
NOSTRIN
OSBPL10
OVOL1
PACSIN1
PHC1
PPP6R3
SSU72
SULT1D1
TBC1D24
TMBIM6
TMEM42
TRIP10
USP49
UST
WDR5
YTHDF3
INTS3
PPM1J
ZBTB18
ZBTB40
BCL2L10
IFIT2
PARP3
SMO
SPON1
ZNFX1
CCT3
CPEB2
CREBRF
DHRS9
AAMDC
SERPINA1C
C2CD4A
MIR1894
ZNF215
ALOXE3
CACNA2D1
COMMD3
DRAP1

IFI205
LRRC36
NBEAL1
NUP214
PAM
RAB21
RIMOC1
SERPINB13
SHISA8
SPINDOC
SSUH2
ZC3H12C
ZNF581
ARHGAP10
BOLA1
C9
CENPL
HCFC1R1
HDAC10
HINT2
ING5
KCNN3
KLHDC2
LSM10
MAGEE1
MEIG1
MRPL3
PPP2R3B
PRMT6
RABL6
SLC25A34
STON1
STXBP5
TMEM209
UBA3
USP11
VTI1A
WBP1
ZBTB7B
ZFYVE1
PBLD
FUBP1
GRB2
PARP14
PTGER3
SOD-1
WBP2
CYTH4
BANK1
BSN
CACNB1

GLMP
GRHL3
KISS1R
KMT5A
MRPL54
NAPSA
RGS7
RRN3
SUSD3
TMEM154
UBE2E2
ZMYND11
ARHGAP20
FAU
IWS1
MGAT4A
PPP1R9B
SH3TC1
TRIP6
TUBE1
CEBPG
CYP2A5
INMT
PDXK
FAM47E-STBD1
ADGRF2P
UBL4A
H2AJ
SMIM2
HSD17B3
IGKV12-46
KDSR
LUZP1
ZNF101
APC2
BCL2A1B
GSDMC
KIAA1614
LILRB1
SUSD4
ZFP53
ANKRD23
ARHGAP40
GID4
IQGAP2
LCE1F
MIR92B
SBF2
SLFN8
STX16
ZCWPW2

ZFP366
ARFGAP2
ASXL2
CBLC
CKMT1
CTNNA2
DENND5B
DYNC2I2
FKBP9
FUCA2
GDF6
HBS1L
HK3
KIRREL1
KRT6A
MFNG
MRPS17
MYOM1
NDUFB4
PHC2
PTK7
RAB13
SASS6
SH3BGRL2
SLC39A5
SPATA7
SPTA1
STX2
TNS4
TXNL4B
VAC14
VWCE
WFDC1
DOK4
FRMD3
POLE2
ALAS2
BRI3
CRABP2
IQGAP1
SCOC
SHANK2
ZNF35
ASGR2
MYBPC1
NELL1
ANKRD50
ARHGEF9
CBLL1
CDK16
CERS4

DGKZ
HOXB5
LAMTOR3
MRPS28
P3H3
PCNP
PLS3
PRPF39
SGSM3
SNX4
STK40
SUGT1
TIAM2
VOPP1
YARS2
YIPF5
DENND1A
RNF122
CHMP4C
GALT
HOMER3
RNASE1
OTP
KDM5D
LINGO3
NYX
RBPMS
WNT6
ANKRD45
DYNLT4
IFI27L2
NOC2L
POU5F2
TMEM232
ZFP704
ZPBP2
ADAMTSL2
CAB39
CCDC18
CDK13
CENPT
CRHR2
CYFIP1
DHX37
DOK3
EIF3D
ELAVL3
GNB4
GPD1L
HSPBP1
ILF2

KDM4C
NAGS
PHKA2
RASA4
RHOBTB2
SHC4
SLC6A11
THOC1
TMEM87A
WDR36
GPER1
NKX2-5
ACSBG1
AGFG2
HPDL
SPOCK2
PLXNA2
PSMB10
SMC1A
S100B
MIR1290
SNTG2
ARL5A
ATP9A
NEK7
RAP1GAP2
STMN3
ZSCAN32
AGAP2
ARHGAP25
ATN1
BBS9
CEP-1
CUL4B
DGLUCY
EIF2B1
ERG28
EXOC2
JPH3
LASP1
LSAMP
MUSTN1
NDUFA6
P2RX2
PA2G4
PPIH
PTRH1
PUF60
SERPINA3K
SMG1
SNHG12

SULT1B1
UBE2O
ATAD2
PDCD6
FFAR1
GAL3ST4
JHY
MIR708
NCK1
NOP9
RDH5
ZNF711
ACTRT2
CCDC150
CHADL
DNM3
FSD2
GPR63
H2-M3
IQSEC1
RABL2A
TLR13
ZNF91
CCNY
DEXI
DVL3
GUCY1A2
HDHD3
KDM2A
MANEA
MRPL33
MYCBP
PATL1
PDSS1
RO60
SRP68
STK26
TMEM64
TXLNA
UNC13D
XPO7
ZFX
WNK2
CSNK2A1
FAT1
HES6
POLR3K
THBS2
BAALC
MAN1C1
EFNB3

FAM156B
GPR176
TXNDC16
EPDR1
DNAJB9
DRD5
ARHGAP28
ARMC9
C8G
CENPQ
CIB1
DCHS1
DNMBP
DTNB
EXOSC4
FAM111B
HPS1
KLHL13
MIA3
PICART1
PRPSAP1
RCOR3
REEP3
SMIM6
SNX5
STYXL1
TMEM229B
TMEM39A
TRIM35
WASF3
IGKV6-32
COL11A1
SNORA20
AKR1C14
C1QA
CCDC88C
KLHL42
RIC3
SLC6A8
THBS3
TXNRD2
TEPSIN
ZFP346
MAIP1
MIR1915HG
MS4A4D
SSPO
STFA2L1
TMEM178
ZNF557
BTBD19

CWH43
FAM163B
PNPLA5
SAP25
SPRR2B
TCF24
ZNF770
NUDT21
SPSB4
ARHGEF39
BET1
CEP85
COX8B
DLGAP1
H3C1
HOOK2
KCNMB4
KHDRBS3
MRAP
PALS2
PELP1
PHF14
PLCXD2
RNPS1
SAE1
ZCCHC2
CAND1
SLC17A8
CLDN3
CNR2
NRCAM
TRAK1
DVL2
SIAE
ARIH2
CTCF
DCAF6
FRYL
KRT20
OSBP2
PARP8
SENP6
TOR1AIP1
TRIM13
TRRAP
VPS26A
CNR1
GNG5
IRX1
NRARP
NUP37

PHOSPHO1
RCOR2
SLC30A3
SLC35G1
VPS13C
XPNPEP1
ZFP810
TSPOAP1
ANKRD13C-DT
HOTAIR
POU1F1
SIGLECH
SPRR2F
TESPA1
YJU2
ABCB9
ANO8
ASTE1
CYP4F15
GOT1L1
KXD1
VCF2
ZFP276
G3BP2
PPM1H
RPTN
SHISA6
TFEC
ATP1A2
CDK18
MTFR1
TAB2
TDRD7
WNK4
AOX3
COLQ
DAB2IP
GALNT7
KDM4A
MRPL50
NMB
NOP16
PHTF1
S1PR5
SEC61B
SLC44A3
TLCD4
ANO3
ANXA13
RFK
CYP2C29

OPRK1
PTPRR
AQP4
DDX21
UBD
ALPI
EGR
ZNF549
CLEC11A
EYA4
HTR1A
RPL35
SNRPE
SRCIN1
TAF9
WTAP
ZHX2
BICC1
MPI
MYH3
RBM12
SHROOM3
ATP1A1-AS1
MYBPC3
SEC13
SWINGN
ARXES2
C1QTNF1
SH2D7
EFNA3
RASGRF2
SMIM12
TMEM181
UBXN10
ZNF561
AHSA2
AP4B1
AP5B1
CSF2RB2
FAM185A
MED9
PPP1R36
SCGB1C1
SPAG8
TNFSF18
CCNI
LCE1A
NCK2
PGAP2
RRP9
TENT5B

TOR1A
ARFGEF1
FAM177B
FAM222A
GEMIN6
HACD4
LCE1A2
LSM6
MGAT4B
MKNK1
SLN
TMEM86A
USP24
CD200R4
GDPD5
DDX59-AS1
EPHB6
GCK
CYP2AB1
LCP1
ZFP354B
GYPC
HACD1
IBSP
KLHL29
OSR1
PEX2
SCN3B
SPEN
APOBEC3A
ARHGEF6
ATAD5
BTK
CHST3
DTX3L
EPHB3
GLI4
GLRX2
GOLT1B
HTATSF1
MOGS
UBASH3B
ZNF669
VAMP1
TGOLN1
ZDHHC22
C6ORF62
ERICH3
FBXO48
HCAR1
HIPK4

HMOX1A
LMOD2
RNASET2
ZNF12
AGR3
ANKMY1
APOL7A
FCGR4
MORN5
NEURL1A
RAB9B
SLC25A40
SPPL2B
SULT6B1
TIFAB
TLR12
CD99L2
GBE1
GSTK1
NPIPB3
SOSTDC1
SETD2
ATRN
BRI3BP
CARD11
CDK2AP2
CKAP5
CPNE3
PGP
PSMD6
RGS19
RNF103
SEMA4A
STK38L
TMED9
TRAM2
GABRA1
IPO9
BIRC2
PAK1IP1
PDGFD
VTRNA1-1
HAPLN1
N4BP2L2
NXN
TRP53INP1
ADAP2
AEBP1
ALDH1B1
ARID4B
ATAD3A

CDS2
CSTF3
DDX3Y
GPR153
HEY2
LRRK1
MAN1A2
MCOLN1
NPR1
NPTX2
OBSCN
PELO
PPP2R2C
PPP3CC
SH2D4A
SPHK2
TCEAL8
GABRR2
MIR504
PSG4
CFAP300
CNGA1
FAM89B
GSG1L
LHFPL4
BNIPL
CD8B1
CERS3
CYP4F16
GSKIP
ITGAD
NEK11
PHOX2A
RIBC1
ZNF326
ZNF787
CFAP57
DDAH1
NOCT
RNASE4
PREPL
SMARCC1
AFDN
CAMKK1
CYP3A13
DHX30
DIXDC1
FGF5
JARID2
MOB1A
NUP58

OTUB2
PASK
PHTF2
PRKRA
PTPRB
RIPK4
SLC29A3
SUV39H2
THNSL2
VANGL1
WDR76
YPEL1
AGAP1
DNAJC19
GNS
LRRC8D
NEUROD1
SLC2A13
VPS37B
ADRA2C
BARX2
FMO3
LHCGR
MYBL1
RAET1G
SH3RF1
ABHD11
ABHD14B
CD209
CENPH
KCNJ5
KDM5A
MICALL1
NBPF26
NDUFA3
NEK10
PIEZO2
PKDCC
RPL17-C18ORF32
SH3PXD2B
SIDT2
TMEM171
CCDC24
CNBP
ZNF57
ZNF573
ABCA17
FLT3LG
KREMEN2
NKIRAS2
NRDE2

NTRK3
SLC31A2
TNFRSF6B
UBOX5
VSIG1
IGIP
PTX4
ZFP12
ZFP959
ZNF527
SLIT1
LDLRAD4
API5
CCNC
CHCHD6
HAUS4
NDUFS3
PXMP4
R3HDM2
RAB6B
RBBP7
RFFL
SNRPG
TP53INP2
TSPAN15
RASSF5
CELSR2
ZFPL1
FPGS
H3C6
NAA38
PGLS
PGM5
PLK5
ADAT3
AHCYL1
GINS3
GLYCAM1
GRIN2D
GTF2H1
HIP1R
INTS4
ITGB7
KCNJ14
KIF21A
MAB21L4
MRFAP1
NME7
PGPEP1
PLEKHO2
PPP1R15B

PTGES2
RAB29
RASGEF1B
REXO5
SLC13A4
SLC35F2
TMEM176B
TMEM33
TNFRSF13C
TTC39C
YRDC
AHSA2P
BZW2
CCDC13
CCDC74A
EXOSC1
FBXL15
KRT79
LGSN
PODNL1
RNF32
ZNF43
HAVCR1
PNPLA7
SDCBP2
GCAT
PRPH
STAMBPL1
ABCA8A
ADGRL1
ASCC3
ATP6V0C
DOCK5
FKTN
S100A13
ESRRA
FCAR
HJV
LLGL2
CCT4
IFI47
SLC6A15
ARRDC3
GREM2
SIKE1
STARD10
TTLL7
CHST13
ERMN
BBOF1
CABCOCO1

CARNMT1
CCDC6
COX7A2
CPLX1
FKBP14
LAMTOR4
LIMS2
LPAR3
MAP3K4
MIR1927
MYO5C
NDC1
NDUFA1
NLN
ODAD3
ODR4
PLPP1
PPP1R1A
SLC28A2
SPECC1L
SVOP
TCEAL3
TMEM140
TXNDC15
CWC15
HHIPL1
SOX15
TTC4
ZDHHC6
OTUB1
PIP4K2A
TUSC1
ADAM22
HCFC1
IRF2
PPIL1
PTGES3
SGK3
TEDDM3
UQCRFS1
C20ORF204
DTNA
HEBP2
HS3ST3B1
PRL2A1
SCN4B
SIN3A
SLFN12L
TSHZ1
HSP70.3
IGKV15-103

ZFP558
GAS6-AS1
ATF5
COPRS
CSN2
MUC21
SLC25A20
ECT2L
H3F3A
LRPAP1
RPS16
ZNF467
ZNF624
AAR2
CISD3
DCAF12
ECSCR
GTPBP10
GUCA1B
HES5
ITM2B
PGAP3
RASGRP4
TIGD5
VAV2
ACOT13
APBB1IP
CCDC92
CDK5R2
FMNL2
GDPD1
GRHPR
JPH4
LACTB2
MAMSTR
MRGBP
PRELP
PROSER1
PSKH1
R3HDM4
RCC1
SMCHD1
TOB2
TSPAN13
TTC22
F10
ZFY
ZNF628
GAL3ST3
PRR22
RPL27

CPLANE2
LINC00334
CCT6B
STRA6L
TMEM106B
TOR3A
ARID1B
CDK9
FARSA
FAT4
JADE2
MPG
PLEKHA2
PRG4
ROR1
RPL26
SPN
TUBA8
UBQLN1
ACP1
RPS21
SLC12A5
TOX3
MTIF3
SH2B3
H2-Q10
HGD
MYL5
PDLIM3
PRORP
AHSP
SCN2A
ZBED6
ZSCAN18
ZSCAN29
AKR7A5
ANO5
ARMT1
FAM220A
MINDY4
SCGB3A2
TACO1
TOGARAM1
ZFP1
CASP8AP2
CRNKL1
FGF12
GCNT1
RNF214
RTL5
SLC10A5

TNFRSF10D
TYK2
KHDRBS1
NOC3L
RAB6A
RBX1
TLE4
TMOD1
FJX1
CAR2
MAPK12
MOCOS
NIFK
HSP70
IGSF3
PKP4
TIGD3
WASL
ZNF92
ATP5MF
CES3
F12
FGFBP1
HSPB7
PCYT1A
PFAS
PLAA
SLC43A1
TAGLN3
CNTN1
METTL15
MYCS
AREL1
CLXN
DDX55
EIF2D
IFITM10
MEF2B
PAEP
SNX20
TRAPPC11
TSPYL5
BIN2
CHMP3
COMMD8
CYP2J9
FADS2
FCSK
IGLON5
KCTD2
MIR203

MYLPF
NLGN2
SERPINF2
SNPH
TRIM41
ZC3H12D
ASCL1
NDUFAB1
SNAP23
AGXT
DUSP16
FZD8
GLI3
LMO4
MTAP
NRIP3
PTTG2
RPS9
SLC22A7
SNORA64
SUB1
TMSB4Y
WFIKKN2
CMTM2
FAM221B
FGFBP2
SOD-3
ZFP560
ZFP931
CKMT1A
GNA14
GVIN-PS2
MIR3176
RABGGTB
SMBD1
CNTF
CXXC5
PARP9
CA5B
ITIH1
TNNT1
ADNP
BAIAP2L1
CADPS2
CDC42BPA
GNPDA1
HELZ2
HIP1
KCTD8
KPNA4
NCKAP1

NIT1
NUP98
OSTM1
PLD2
RPS25
SLFN5
TMCC3
GRM6
MZT1
RC3H2
TBR1
ENPP3
FAM53A
KLHL32
MS4A2
TCEAL7
ATMIN
C4BP
CACTIN
EFHC2
ENKD1
IFTAP
IGHG1
LRRC61
MID1
RBM48
SHARPIN
TGM5
TMEM63C
TRAF7
DCST1
KDM8
MSS51
NRBP1
PRPF40B
ATL2
PTPN12
RETREG1
ADGRG2
GOLGA4
KDM5B
RCC2
RPS10
STRBP
MIA2
SLC15A2
RPS5
CNN2
PPP2CB
ARL14
CHD4

COX6B1
DAB1
FAM20C
GRB7
KTN1
ORM3
PDE1A
RDH11
RPL36
RPL37
SLC13A5
TMBIM1
ZFHX4
CHPT1
DACT2
EMP3
FGF22
GP1BA
INHBE
LPAR5
RAB42
SDR39U1
UBP1
ANKLE1
AP5S1
DHX32
DNAAF9
FOXE1
GGA1
HEPACAM
KLHL30
LOXHD1
MINDY2
NUP188
ORC5
SMIM5
TMEM184A
TMEM216
TRIM56
UTP23
ZNF394
B4GALT5
PCYOX1
PON3
RDH10
ANGPTL3
CRY2
EVL
F8
HTT
MAP2K5

PCNX4
RNPEP
SLC27A4
SUCLG1
C11ORF58
HSPE1-MOB4
AHRRB
ATP10A
HCN2
STFA1
C6ORF52
YJEFN3
CTGF
H2AC19
SPRR2A2
CD209A
EIF5B
FRMD4B
GASK1B
RBMS2
SCG2
SKAP2
SKINT3
YAE1D1
BTF3
ELP3
EPB41L4A-AS1
FCER1A
GP9
GPR137B
HPD
KLK3
LRRC25
MAD2L1BP
NMT2
PGAM2
POF1B
PSME3
QDPR
RPS8
ULBP2
WASHC2C
ZFP14
ACAP3
ARMCX4
BAHD1
FAM221A
FCER2
GNPTG
GZF1
HSF4

MSANTD4
MUP5
MYH7B
RNFT1
SLC25A38
SLC35C1
STIMATE
TGTP1
YIPF1
CES1
ACY1
EOMES
FBP1
HOXA1
NR0B2
QPCT
WNT11
ZNF136
ADAMTS2
ASPA
BACE2
GBP4
KCNN2
KIFC3
ONECUT1
BDH2
KYAT1
CASTOR2
CCDC159
PCSK6
PTCHD4
ADIRF
CST7
DOLPP1
GJB4
GRIPAP1
HTR7
SLC22A18
ZNF589
APH1A
ASNSD1
CAR13
CCDC137
CFAP53
DHDDS
DNAI4
GLB1L2
GPM6A
HSBP1L1
KALRN
KCNG2

MARK1
MCCC2
MIIP
MTMR9
PCSK7
PDE6D
PEX11G
PIBF1
PPP2R3C
PYGO1
SERPINB6A
SRSF10
TCF23
WDR13
WFDC21
YPEL2
ALDH1A2
NUDT4
PREP
RINL
SNRPD1
TANGO6
TRIR
SLC2A1-DT
MIR1225
EPB41
AK8
CLGN
COBLL1
EXOSC8
FXR1
INA
KRT17P3
PCCA
PSD3
SULT2B1
TRIML2
TSPAN8
GDF1
ME1
ARL5C
HMCN2
LONP1
LRRC75B
NYAP1
ST6GALNAC1
SCO2
ABCC12
ALKBH1
APBA3
CFAP36

CIBAR1
DDX19B
FAM47E
JRK
KBTBD7
LAX1
MRPS23
NPAT
PIK3R6
SP140L
TEX15
TRPM1
TTC7A
ZNF451
COMMD5
EBAG9
GTPBP6
KIF24
MOB2
NIPSNAP3A
OTOF
PDE6H
PRKD3
TCERG1L
CCPG1
EIF3C
KIF25
MYO1E
PYCR1
RNASE2
SATB2
SPTBN2
ZFP740
EAR6
FAM216B
KRBA2
LCE2B
MIR376A
PRL3A1
ZFP831
C8ORF76
IFT70A1
SAMD14
SGO2A
CTSQ
FOXO
NXF7
PCDHGC5
PSPH
VSTM5
CYC1

CYP39A1
GNE
PPFIA4
PTPRN
RPS13
SLC39A4
SLC6A12
SLK
STK10
YY1
LIMCH1
IFI27L2A
VPS39
YIPF6
CREG2
DENND4B
FAM83G
GATB
IFIT5
MKS1
NPHP3
PIGS
SGIP1
TATDN1
TOR2A
UNC50
ANKRD13D
CARD19
CENATAC
CHST10
COX18
CPAP
CTDP1
CYP4F12
DNASE1L1
FAM171A2
GLMN
IL22RA2
IMMP1L
JKAMP
MRGPRF
MRPS15
PCGF3
PDIK1L
POLR2K
PRND
PTH1R
SEC22C
SF3B6
SLC25A43
TFAP2E

TMUB1
TOMM6
VCPKMT
VPS26B
TJP3
GRINA
PAPSS1
PHLDB2
PLEKHD1
IPO5
MANBAL
RBMX
ACOT4
COQ10B
COQ8A
KCNAB1
LGR4
HC
LARGE2
LSG1
RNF20
ZC3HC1
CTSW
CYTH2
FAM98B
IQCA1
LRRC14
PRSS27
RBM12B
RIC8B
RSAD1
RXFP2
SCRG1
SDF2
TAF10
TAS1R1
TMEM60
VPS37D
ZBTB49
ANKS6
ATAT1
CNTNAP3
DHX57
DSC1
EML6
IGKC
KCNN1
LCA5
LIME1
MAU2
MID2

MPZL3
NPFFR2
OLFM2
P2RX6
RCCD1
SPRYD4
SYCP2
TIMM22
TMEM231
ACSF2
BORCS6
HRK
CYP51A1
SGK2
KCTD12
MAL2
USF1
DENND10
PXDNL
C21ORF58
DIPK2B
NPIPB5
ZNF239
MIR590
OSER1-DT
PSG16
TRBC2
ZNF440
C1ORF35
INTS13
RPA4
SATB1
SEMA6D
AB124611
BCL2L2
COMTD1
DNAJC10
GBP2B
GUSBP11
IP6K2
PDIA2
PRDM16
SRP14-DT
STMN2
TAF6
WDR49
ZDHHC11
ZNF696
ADO
AK9
AP1AR

AUNIP
CYP4F18
GPS1
GUCY1B2
HCN1
KATNAL2
MAP9
MAPKBP1
MRPS25
MVB12A
NEK3
NOTUM
OGFOD3
PI15
PUSL1
SAV1
TAF12
TM4SF20
TMEM35A
ANGPTL7
ANKRD13B
AURKAIP1
DALRD3
DIRAS1
EDC4
ERICH1
GTF2IRD2
RGL3
SLC16A8
IGFBP1B
ACADSB
CYP3A65
DNASE1L3
ETFDH
KMO
MEOX1
PTPN13
RAI14
TRGV5
TRIB2
CYFIP2
MIR500
PYGB
AMH
LSM8
MAPK1IP1L
NOS2A
TARS2
ADAMTS17
AIM2
ANKLE2

ATP23
CNTROB
EDRF1
FBLN7
FMNL3
GABBR2
ICAM3
MRPL58
POGLUT3
PYROXD1
RALGAPB
RBPJ
RHBDD3
SP140
TAL1
USP21
AMY1
ARL10
CCDC180
CLEC2B
COL27A1
CPA3
EMC7
H1-2
IQCB1
MFSD12
MIR3070B
PLD6
PLSCR3
PRRT3
RSPO2
RUBCNL
SNAPC5
TBC1D22A
TMCO4
TMEM11
IRF2BP2
ZFP626
NFATC2
SPSB1
CCDC80
KRT14
TLN1
UEVLD
SEC24D
ARHGEF35
IGKV16-104
LRPPRC
PITRM1-AS1
POLA2
SIAH2

DHX8
IGKV4-72
NARS1
PHETA1
RAET1K
ALG9
NCOA1
THEMIS
TNPO3
LILRB2
NDNF
NUDCD3
PEG13
POM121C
SMIM8
SRPK3
WDR59
ADAM28
BORCS8
CBFA2T2
CCDC15
CELF3
COQ6
COX4I2
DPYSL5
FAM110A
GTF2IRD2B
IFNL2
IPO13
IST1
MARCHF8
MTX1
MYO15B
NGLY1
NKIRAS1
NOL7
PHF7
PHGR1
PTCD3
RD3
RIMS4
SMG9
SNHG20
SNORA73A
SURF2
TBX18
TESMIN
TRNT1
ATP6AP2
BUD23
CNDP2

CTIF
MRM1
PNP2
PPFIBP1
RALA
SLC9A5
RABL2B
SLC16A2
ZNF662
AWAT1
KIR3DL1
PRL8A9
SUMO4
ADAM30
PPP5C
PRODH
RPS15
OLFML3
APOC2
DHRS11
GAS7
GCM1
H6PD
HIBCH
PDCD6IP
SULT1C2
SMU1
BRSK2
ENTPD2
GPKOW
MLIP
PROM2
RNASEH2C
TRIM2
TRNF
ADAMTS20
ALDH3B2
ARHGEF25
ATIC
BATF2
DNAJC16
DQX1
ESYT3
FCHO1
H2BC15
MED30
MRPL57
MTERF2
ODF2L
PQBP1
PTGDR2

SCML4
SLC35F1
SYDE2
TELO2
TRMT12
VPS9D1
CELF2
HBP1
IGBP1
KLHL3
PHKG1
PSMD10
CLIP1
TYROBP
PITX3
ITGA2B
ATXN1
DZIP1
F11R
NDP
QSOX2
RPL13A
B3GNT3
CABP1
ELT-2
GPR84
HSPA8P5
ICOS
NUDT11
TBC1D32
ADCK5
CPSF1
CST4
CXCL17
HINFP
HS3ST6
KLHL12
LY6K
LYL1
N4BP3
NICN1
NUP43
PTOV1
SETD3
SLC4A3
SMTNL2
SPAG6
SPNS1
SYAP1
YDJC
ZFTRAF1

DDX31
DPEP2
ECRG4
GATD1
KLC3
LRRC4
MYOZ2
TMEM223
ULK4
DLAT
HTR2B
NEB
ZNF772
ANKDD1A
BASP1
BCL10
IGFL1
ITIH3
PDE2A
H2-Q4
LINC00673
LINC01278
ZNF418
C8ORF82
MIR376B
TMEM17
TXNL1
ADISSP
CYP4F11
MAPDA
MIR541
PBP2
PLA2G4D
ANKRD13C
ANKRD24
CEP295NL
FN3KRP
PKLR
SLC35G3
SPACDR
AIFM2
ATP5MC1
C15ORF61
C6
CNOT9
COG8
FAM161A
IFNAR1
NBPF14
PSMG2
RPS15A

SERGEF
SFSWAP
SHE
SIPA1L2
SLAMF9
SLC35A4
SPRR2A1
ADGRG3
ARHGAP44
ARHGEF10L
CDK11B
COA3
DLGAP2
EDF1
GAREM1
H4C1
LRRC37A5P
MCTS1
MED27
NAA35
RFX2
SLC7A4
SREK1IP1
ST6GAL2
TM4SF19
VPS25
CFAP74
KIR2DL2
ZDHHC8
CERNA1
EVI2
GPR141B
ICA1L
IGKV1-110
LY6A2
RPL15-PS3
RPS13-PS1
SPEF1L
CEP63
KRT17P1
SIX2
SPATA31F1B
UBE2E2-DT
MIR450-2
MYH4
OCIAD1
TRAM1L1
ANK3
CLCC1
EPS15L1
HOMER1

LRRFIP2
LSMEM1
MIR4707
MTO1
PGC
VSX1
ZFP62
ABHD17A
ARID1A
CALB1
CSTF2T
DDA1
FBN2
IRGQ
POLR2G
PPWD1
SMYD1
ACKR4
ACTR10
ALDH4A1
CERS2
CHTOP
DAW1
ELAPOR1
EMID1
FXYD7
LAIR1
LMTK2
NMNAT3
PLN
PPM1M
PPP2R5B
SCMH1
SDHAF1
STAP2
STX17
ADRA2A
MIR365-2
OR2AH1
ADAMTS5
SMC2
SNHG6
ZNF146
ZNF267
HOXA4
KIAA1549L
SYN1
WASH5P
ADM5
C15ORF62
NEU2

SLC25A46
ANAPC11
C1ORF56
CCKAR
CTXN1
DNAJC13
ELL3
GGA2
MIER3
MMGT1
RRP1
SGCA
SIGLECG
AKIP1
C6ORF89
CARF
CMAH
CYP2A12
FTSJ1
GALNT13
IRGM1
METTL5
NHLRC3
NIPSNAP3B
PIGT
PLD5
RAB2B
RMND1
SBK2
SLF2
STMND1
TIMM8A1
TMEM70
ZDHHC20
GJC2
H2AC23
IRF9
LCN8
SCAF4
SRP14
ZBTB11
ZFHX2
ZNF836
METTL2B
SH3BP5
CACFD1
ZFP787
DUOXA1
MDR50
RTN4RL2
SFT2D1

BRSK1
KISS1
L1CAM
MAL
NFAM1
SMOC1
STOM
DRAXIN
FRAT1
FZD9
HDDC2
KIF16B
RFLNA
ABRACL
APOO
ARFIP2
CNOT7
DGKD
DSC3
DUS4L
GPAA1
HTRA4
LMAN2L
MARVELD2
NAP1L2
NRROS
PUS10
PUS3
PWP1
RASL10B
SHROOM1
SHROOM4
SNAI3
TMEM120B
TRIM9
ZNF827
FLYWCH1
GJB5
GRIN2A
MRPL52
NKG7
PSME4
SCAMP4
WWC3
ZBED3
ZFP64
YARS1
ELOVL6
ZMAT3
AQP7
MIR939

IFT88
MPP4
AMZ2
BTBD9
CEP68
DCLK1
DHRS7
HOXD12
MOAP1
MTCH1
NOL10
POMGNT2
PTPMT1
RPP38
RTCA
TMPRSS6
TNR
USP6NL
CDKL2
CRNN
FGF14
GMIP
INAVA
LDAH
NSG2
PHKG2
POLR2I
RPP40
SLC35F6
WDR19
WDR81
ZFP628
ZFP90
MIR638
CD209B
DDX11L2
GARS1
LILRB4B
LTBP1
MLXIPL
APOF
ATAD3C
CCDC175
DIMT1L
IGSF21
LTBP2
MAPK1IP1
PLAGL1
SNORD101
TXK
ZNF611

ZNF649
ZNF701
AMY2B
FUT6
PCDHGA12
SH3BP1
SLC52A1
PEX7
PRCD
TTC34
WWC2-AS2
ATP8A2
AZI2
LINC00313
EIF4B
FOXRED2
GRAP2
KCTD9
LEPROTL1
LETMD1
LZTS3
METTL21A
MTMR2
PHAX
PSMB9
RFTN2
RWDD2A
TOMM22
USP45
ABTB1
ALG3
ARHGAP39
CD1D1
CHD5
CRB2
DUSP23
GPR157
LAG3
LMBRD2
MOSPD3
MT1F
NEUROG2
NOL9
P2RY10
PTPN23
RPP25
SLC4A8
AGTR2
CYP26A1
GSTT2
NSF

SECTM1
ASNS
CRYZ
GPR162
ALPHA-EST7
AVPR1A
HTR3A
IL22RA1
LYPLA2
RPRML
STK24
ZFAND3
A1BG
ADAM32
AMN
CARD9
COIL
IMP3
IRGM2
MTPAP
NDUFB1
NOC4L
OR2L13
PLEKHA4
PPP4R1
QTRT1
RAB11FIP2
RUNDC3A
SLC6A20
SMYD5
TOMM40L
ZBTB11-AS1
ANXA9
APH1C
CGGBP1
CLEC14A
DAZAP2
FASL
ICMT
IGKV6-25
INSC
MIR374C
PHACTR3
PLEKHN1
PLSCR1
RAB37
STARD8
TEKTIP1
TRIM33
RPS3
ABCB5

SBPL
ARID3C
C1S1
CCZ1B
CLNK
H2-Q5
PRSS41
ANXA2P1
ATP5PD
C1ORF122
PALLD
PLA2G4E
ZNF554
CCDC103
CRCP
FAM25C
KCTD14
PREX2
SLC49A4
TNFRSF22
TNFRSF23
CYRIB
FGFBP3
GTF3C4
HSPBAP1
JAGN1
MED23
MRPL15
NDFIP2
PABIR2
PAPOLG
PFDN4
PHF11D
POLRMT
RASSF7
RBM34
SEMA3D
SLC22A17
SLITRK5
SP2
TRIQK
TSGA10
DDO
LGI2
MAML1
MKRN1
NSMCE4A
PLPP6
RABGEF1
KATNA1
NFIB

DCAF15
PNRC1
SLC2A5
TPRG1L
SNX17
ALG12
CRELD1
FAT2
KDM5C
PLXNA1
ACSM3
ADIPOR2
ELOA
MOG
REEP6
SHQ1
STXBP5L
TRMT5
ABHD10
ART4
CCP110
CHMP2B
CHST8
DEAF1
DENND2A
GPR85
HPS3
KMT2B
RAB11FIP3
SEMA6B
SEPTIN10
SH3GL1
SHC2
SSBP4
VPS36
ZMYM5
GNG10
RBM18
RILPL1
USF2
LYRM2
SNAPC3
DEK
FAP
FUNDC2
NR2F1
PAK1
XKR8
ZNF587B
KLRA16
SLCO1A4

SNORD43
PRSS16
ZNF585B
ELFN2
MT1IP
TMEM109
ZNF439
DEF8
FBXL9P
GTF2H3
INPP5B
MINDY1
MRPL24
MRPS18C
NFIA
OGA
PEX3
PSG5
ZNF468
ACY3
ATP11B
C18ORF21
C1QTNF2
C3ORF62
CCDC117
CCL21B
CDKN2AIPNL
CHM
CNPY2
FOSAB
GPRASP1
INR
KRT36
MUP9
MYLK3
NUDCD1
OAZ2
POLR2B
RNASE2A
RPS6KB2
SAMD11
SLC9A2
SPIN3
TMEM52
TRF
TSPAN9
TTLL8
ZNF548
ZNF675
ACTL7B
MT1L

ZNF846
KLHL22
UROS
CCDC93
KIFAP3
MDR65
MRPL55
PSAP
UNC5B
KCNH3
MYOM3
APOL9A
CNST
LGALS7
LRRC41
NUB1
NUDT16
OSBPL9
PDCD10
SLC5A8
USP40
ALDH18A1
ASCL2
B3GALT5
CCNK
CD320
CDC26
CES1G
CLDN8
CRTC2
CRYZL1
DBNDD2
GSTM7
GTF2A1
LCE1A1
LONP2
MIR124A-1HG
NPHS1
PCSK1N
PPM1E
SLC7A10
TM2D1
TOMM34
UBXN1
WDFY4
CDK5R1
DPM1
SNW1
TBCEL
ZC3H15
OST4

PMS2P4
C10ORF95-AS1
EGOT
ELP6
KLHL41
LCE1I
RNF126
PRL8A2
CD99
EEF2KMT
ERVMER34-1
FOXK2
LCE1B
MIR4516
ACTL6A
EFCAB8
FRMD5
H1F2
RBM7
CSGALNACT2
DDX56
DNASE2
EGFLAM
GCC2
HYI
JAZF1
KIZ
LETM2
OGDHL
PI16
PLD4
POLR3B
PRR15L
RCHY1
REPS1
SAMSN1
SLC35E1
TBC1D30
ANKRD63
C1QL2
CYB5R2
HRC
NSDHL
PLAG1
SLC22A8
TMED7
TOR1B
CSTDC2
HLA-DRB4
KCTD12B
PEX11A

PRAMEL3E
TRIM30D
TSPYL3
GDAP10
PIRA2
TRIM12A
ZFP668
CSTA3
CSTDC6
KRT17P2
LINC01970
MIR3473A
OCM
PYCARD-AS1
SEPTIN9-DT
ABHD17B
HGH1
MC2R
P4HTM
VXN
ATP6V0A4
GABRA3
H2AC21
KLRA14-PS
NDN
PRPF6
XYLB
ALDH16A1
ATE1
CTSO
GRM8
MLX
NDUFS6
ROBO4
SLC2A2
SLC8B1
UBAP2
ACSM1
ARF5
BCAS2
COL9A3
LPIN3
MCRIP2
MRM3
MT1B
PLEKHF2
SULT1A3
SURF6
WDR54
ZCCHC12
NAGPA

AKR1D1
C22ORF46P
LCE3F
OR4P19
OR4P21
PRPF38B
TSKU
EST-6
ZNF26
ARHGAP27P1
CRTC3
ELP4
OSBP
PAIP2B
PROK1
VRK2
ATG2B
BCL2L14
CTLA2A
FER
KLHL2
NCKAP1L
NDUFB7
NSMCE1
PCDHGB6
RILPL2
ULBP1
ADH6
ATP6V0E1
CDO1
DIP2B
DUS1L
ENOX1
FREM1
ICOSL
KLHDC7A
MBLAC2
MTMR6
SEC23IP
SEC24A
SLC27A1
SULT5A1
UBN2
VEZF1
MZF1-AS1
PARP16
BPIFC
PPP1R14D
SST
C1ORF162
HSD17B14

LRP5L
LRRC10
MIR503HG
NWD1
TMEM213
TMEM88B
ZNF444
ADAM21
DMRTB1
DPYSL3
FCN3
ZNF426
ZNF431
ZNF777
ATG13
CES2E
KLRB1A
PDAP1
RNF182
CBX2
GOLGA3
HIPK1
PI4KA
WAPL
AATF
C10H16ORF89
CMTM8
DDX24
EFR3B
ESRP2
MAP3K10
MIR4521
MRPL45
PRRX2
RNF207
SNCAIP
TCAF1
TFAP4
XYLT1
IFITM3
KRT34
SPATA17
TARS1
FAM218A
FOXD1
H1-10-AS1
HTR7P1
KRTAP5-AS1
PATE2
SBF2-AS1
SBSPON

CACNG4
GDF9
ITFG1
MIX23
RASSF8
UBQLN2
ZNF221
CHD6
CPVL
GNPAT
GXYLT1
PDE12
SNAP29
STAC2
USH1C
ZFYVE26
ABHD15
ARMCX1
CDIP1
DUSP11
ESCO1
PLEKHH1
SCX
TUB
TUT7
RAB33A
ARL13A
ATP7A
CYP4A10
MBNL2
ZNF480
IGHD
MYH13
PAX7
TTC31
ZNF225
CASQ2
BATF
FARS2
KIF2A
MICU1
PPFIA3
SINHCAF
AMOT
DENR
DOK1
ELOVL5
FAIM
GPR160
HBB-B2
KCNT1

LARP4
RAP1GDS1
RBM15B
RBM38
SNX25
TIE1
TMEM14C
TMIGD1
TRIAP1
UNC45B
UQCR10
ZRSR2-PS1
DAGLA
DCAF8
EIF3M
GTPBP1
IRX2
TOR4A
RHOXF1
CCNJ
FAM76B
GUCY1A1
NT5C
SLITRK6
SMTNL1
EPHB1
VCPIP1
ENPP6
EXOC6
GPR132
HACD2
PHF6
BLMH
BPNT2
CMTM7
CREB3L1
MAP4K5
MMS22L
NPDC1
PIP4K2B
RAB7B
S100A3
SIK2
SLC1A1
UXS1
SERPINA1D
COL1A1A
SLC29A4
LOXL3
GPBAR1
HPR

ZNF552
FNTB
PKD2L2
SOHLH1
ABCD1
ACOX3
AP1S3
ARHGEF1
BEX4
C4BPA
CRYBG1
DCDC2A
IMMP2L
ND4
OS9
PAFAH1B2
PPP4R2
RSPH10B
YIPF7
ZNF384
ARHGAP9
BCO2
ENPP5
FAHD1
FAM156A
GMFG
IGHG
ITPRID2
LRRC8A
MIR466
NANOS1
PCDHA4
PCDHB9
PLEKHG1
RALY
SCCPDH
SERPINB1B
SFXN4
TAF13
ABCG3
ARMH3
C16ORF74
CD300LD
GPR174
MATN4
METTL14
MSL3L2
TMPRSS5
UQCC4
H2-T22
TGM7

CFAP73
CHRNB2
CLCA4A
CRYBB2P1
IGHV1-9
MFSD3
MIR671
SPIN1
DPP6
LCK
ERBIN
GGCX
GLTP
LRP11
MCUB
MIR141
PIP5K1A
RBFOX2
RTN4IP1
SLC22A15
SLC33A1
TMEM205
VGLL3
CLTA
CYP-29A2
DIAPH1
GCG
GPX-6
PLA2G15
SS18L2
ABCA8
ACADL
BNC1
CYP1C2
SDE2
GFM1
MATK
ND3
TGIF2
AP3S1
C9ORF152
CDKN2AIP
ECHDC3
ELF2
ELMOD1
GRIK3
LARP4B
MRPL49
P3H2
PEX6
RANBP2

RBM26
SETD7
TRPM6
ADGRF5
ATP2B3
C1ORF216
DBNL
DEGS1
ECI1
H1-4
IFT27
MIR636
NCAM1
RAB8A
RAET1L
SLC25A19
SPIN2A
STIP1
STRN
TMEM100
TYSND1
ZNF317
CD164L2
H2-Q6
LINC01588
NAIP
OAS1G
SLFN9
ZNF248
ANXA8L1
ARHGAP32
CCDC191
CIB3
GXYLT2
IGH-VJ558
KIR2DS5
MZT2B
SDHAP2
STPG3-AS1
APOC3
C11ORF24
GIT1
REP15
ANKRD9
CAPN8
CCDC144CP
CES2C
GPRC5D
HSPA4L
NSD3
RGPD2

SEPT8
SOX9B
SSR2
ARHGAP1
CD244A
FAT3
GABRD
IGKV1-132
ITPRIPL2
LINC00957
NEFM
NPIPB11
PPP1CCB
RPRM
SEZ6
SKAP1
SNHG1
SYCP3
TBC1D14
TMPRSS11G
TNFSF13OS
TOM1
WDR45
ARF3
C1QTNF3
ERG
FMO4
FUT1
HOGA1
LRRC53
MAS1
NCSTN
SIRT5
THEM6
CYORF15A
ARFIP1
HOXA2
IFT57
KSR2
MIR92-1
NAPA
RPTOROS
DNAJC21
MUG1
LUC7L2
MLLT10
ZNF227
ART3
EXTL1
LACC1
MRTFA

POLE3
PTPN18
SELENOI
SHROOM2
SNED1
ST7
STAB1
TMEM106A
TMTC3
ZBTB4
GOLGA2
MRPL1
OR5B98
OR7G17
NKX2-6
SLC22A13
VSIG2
ANKK1
EFCAB10
LYPD5
RPP38-DT
RT1-S2
SAMD3
SPATS1
CCDC96
GIMAP3
GIMAP9
PHF11B
PLCD4
ZNF792
ZSCAN22
CIDEB
FAF1
FIGNL2
FITM2
FTSJ3
INCA1
MIR3095
RFX5
SKIV2L2
STK31
ZFP318
ZSWIM1
ARHGEF12
DNAJA3
EBF3
EVI5
LYNX1
NDUFB5
SCAF11
SEPTIN6

BCAP29
JPH1
MCOLN3
TBCB
UTP14A
EFCAB3
GABRB2
GSAP
RHO
ALPP
GALNT18
PIK3C2G
ATP6V1H
CHST14
CREBZF
DAPP1
FCGBP
MLC1
NHSL1
NRAP
RALGAPA2
RNF14
STAC3
TMEM135
VWA5A
CNKSR3
ETV3
FILIP1
MDGA1
MED13
RIMS3
SGTA
UBE2Z
VPS51
ING1
MUCL1
MVK
AMER3
IGH
SLC25A11
XRRA1
DLEC1
DPY19L2
PPM1L
SAAL1
SAMD7
TEAD2
ZNF670
CCDC3
COL9A2
SPATA33

UBTF
UGT2B15
CPPED1
ERP27
GBGT1
LRRC20
NAA50
TMEM72
TRIM67
WDR62
ZPR1
CRYBA2
F2RL2
IL27RA
MLF1
MTPN
NMI
PBX3
RAB10
RASSF3
CLDN16
UGT1A8
CTNNBIP1
FER1L5
MSC
PRL4A1
RENBP
CES1C
DIAPH2
RAMP3
ARMCX3
CYP20A1
FKBP10
GRWD1
PDS5B
RPSA
USP47
CLEC4G
COX15
DPAGT1
GSG2
MALL
MSRB3
PLEKHG3
RAPGEF5
RYR3
TM9SF3
APOBEC3D
FUT3
SUPT20H
TMEM147-AS1

VPS35L
C5ORF24
VCF1
PITX2
SMYD3
TREM3
ZNF569
AKR1C2
APMAP
SNX31
CERK
CFAP65
CUTAL
DENND2B
ELF4
GLRA1
KLB
KRCC1
LRIG3
NDUFB10
OCIAD2
RPS23
SERPINA5
SLC17A1
SLC2A8
ST3GAL4
TCN1
WSB2
ZNF142
ZNF432
CSNK1G1
EPB41L4B
HERC3
PDHX
PNPT1
SIGMAR1
TIMM29
BSPRY
OXT
P2RX5
TMEM255A
TPH1
RBM8A
BCL6B
FBXO21
MGAT2
PMS2P3
RNF43
TNFA
IDH3B
KLHL24

NCAPD3
CMIP
DERA
GOLGB1
GOLM1
KAT6A
NIBAN2
NMNAT1
RYBP
SLC22A6
SNRPD3
UTP20
ZBP1
ZBTB21
UTP4
NEUROG3
PMPCB
SFMBT2
SRPK2
ZNF10
CDC37L1
GATAD2A
NDST1
RLF
C12ORF75
CCDC56
CYP2B2
GCA
ISOC1
MIRLET7A1HG
MUL1
NOTCH2NLC
POLK
SMN2
ZNF180
C9ORF85
CCDC34
CTBS
DAGLB
FAM149A
RPS28
S100Z
SCIN
SNORD22
TEKT4
ZNF469
C8ORF58
CERS1
FOXI2
ITGA9
MYOZ3

NFYC-AS1
SCD3
STFA3
STYXL2
TMPRSS11D
ZFP219
HSP90AA2P
MAPK4
NPW
SNORD115-11
EAF1
HERC4
KLRA13-PS
MIR135A1
NLRP5-PS
RPL14-PS1
SIGLECF
SIRPB1C
IL6RA
LINC01303
LINC01589
PRECSIT
TRIM22
ZNRF2P1
ARF6
CETN3
INPP1
ITM2A
LYST
POLR1C
RPL36A
BTD
C19ORF67
CRYGF
DMTN
DPP3
PLCL2
PUM3
RAB3D
SMPX
TMX1
TRIP12
ZFP322A
SULF2
ADD2
CEBPA-DT
CFAP46
POU2AF3
SCLY
MMAB
BCKDHA

CLN8
DUSP14L1
ECHDC1
SLTM
TRPM2
ZNF682
ARIH1
EIF4E2
LCE1H
MFAP3L
MIR365-1
OR2G7
PRR18
PTGFRN
SYBU
TTC39B
TULP2
DUOXA2
FAM200B
KCNH4
MGARP
RNU1-1
SYNRG
CAGE1
CARD16
COPS9
CYP3A16
FBXO10
ISOC2B
PGAP4
PITPNA
SCN9A
WNT3
ZDHHC15
ACVR1C
LRRC71
PIPOX
SCRT2
SMIM26
DHRS7C
MIR331
AADAC
EIF3I
ERC1
NEURL1B
VIL1
EMG1
LIMK2
MRPL19
NAGA
RPS4X

SLC4A11
UCHL5
VAMP5
VPS13A
WARS1
ACYP2
NPTN
SERPINA1E
ACOT2
H13
ND6
RFX7
TYRO3
UROD
ADGRE2
MS4A4C
ABR
ASAP1-IT1
CHLSN
CHSY1
CPB2
FAM162B
KCNK2
NFATC4
SP5
TIMM9
ACTRT3
AKNAD1
B3GNT5
DYRK1A
NANS
ZNF574
LRRIQ3
MIR101A
SLC22A12
ZKSCAN2
B9D2
C16ORF86
CBX3
CCDC116
CYP6A2
IGFN1
NDUFS7
NEU4
SEC14L5
SPRR2G
SPRR3
T
THAP6
TREML4
ZPLD1

DNAJB3
NME3
NUDT17
PIERCE1
SH2D1A
TMEM120A
TRP53I11
COL4A5
FAM110C
ITLN2
MUC12
NP4
PBXIP1
PHACTR1
RHPN2
SNRPB
TAOK3
VAV1
CYP2F2
OMD
PABPN1
RPS29
TWF1
UQCRB
GPR75
MIR1915
YJU2B
C10ORF95
MLYCD
ITPKA
SNORD96A
ATP8A1
DMGDH
MAPRE2
MEOX2
PTH2R
THOC2
TLCD1
ATF1
CRCT1
FGF16
FXYD1
SPTB
TIMP4
TPST1
HABP2
AGPAT4
BMX
CDK15
ND2
ZNF692

ADAM2
APOBEC2
CCDC142
CKMA
SMIM29
ZFP46
ZFP521
ZSWIM3
CAR12
FAM131C
PTPRJ
STK4
ECHDC2
GMPS
H4C8
HEBP1
NLE1
OLA1
RBM20
SMIM3
SOX6
ETF1
LYPD1
SLC27A2
DCAF12L2
MOSMO
RAB44
FADS3
FOLR2
PTGR2
C3ORF80
C6ORF120
CDS1
LINC00115
MIR302A
SPATA5L1
TMEM91
ZNF189
ACOT9
ASPG
AXDND1
CATSPERB
MYEF2
NLGN4Y
OXR1
RIN2
ATP6V1C1
SOX8
TBC1D29P
TPD52L3
ANKS1B

CREB3L3
PGAP1
TMEM191A
ADSL
CD300LD3
CRMP1
CYP4F22
DNM1P35
FGF8
GCGR
GP6
HAO1
IGHV14-2
KRT87
LINC00240
MUC15
TCEANC
UBE2D2B
ZFP991
ZNF263
CD1E
CETN4
FOXN1
GH1
LBX2
MAGEL2
NDUFB6
RALYL
RPPH1
SIS
STX5A
TCRD
UTS2R
ALOX15B
EML2
GOLGA8O
LRIT1
MIR499
MORN3
SNORA72
SSX2IP
STS
UMODL1-AS1
VPS26C
CLEC18C
PAFAH1B3
RLBP1
ULK2
CISD1
D7ERTD443E
HIST4H4

ABHD3
HMX2
KRTAP10-10
APPL2
IFI27L1
PIRA6
ADM2
HIST2H4A
IMPA1
KAT6B
OBOX5
PHKG1A
SEC23A
STX19
TIMM8B
UGT5A1
ZNF45
APH1B
DEFB6
DNTT
OR2N1
RPL17-PS16
RT1-T24-1
SIRPB3
SNHG3
CALHM5
DDX6
DTX4
LHPP
EEF1AKMT1
HNRNPF
NBPF10
RAPSN
RIMBP3
SSB
ZFP655
EPM2AIP1
FAM178B
HEXIM1
LPGAT1
NABP2
NTSR1
PGRMC2
PIK3C2B
RCE1
RPL30
RPS18
SNAPC2
SNX32
SYT10
DENND11

FLI1
GALNT6
HMGN3
HNRNPR
LINC00339
MANBA
MIR665
PHETA2
PSMC1
TMEM145
UBA5
ZDHHC19
HAGH
LY6G
WFDC8
AMTN
COL13A1
KLF3
CLIC3
CSNK1E
CYP19A1B
KIF1A
PRKCI
RCL1
WFDC21P
DDX46
GALM
HMGCL
IGHA1
IL37
MAST4
SEC62
AKTIP
EPSTI1
KIAA0513
LTB4R1
XPOT
TMEM121B
TP53AIP1
SNX19
ASB6
C1QL3
TCEANC2
TRAPPC5
ACAN
B4GALT3
ELF1
FCRLB
FTL1
KTI12
NKX3-1

RBM5
SLC12A7
SLC49A3
THUMPD2
DNAJA4
ERO1B
FBXO39
LARP1B
MIRLET7BHG
MYPOP
SLC10A3
SMIM11
THAP12
TMEM229A
COA4
H4C3
EGR4
F8A
FAM222B
AJUBA
CPZ
ENDOD1
MACROD1
PPFIBP2
PSG8
SLC39A6
ZNF79
CXCR7
DECR2
GSPT1
HEXA
MMP19
SNX22
UOX
CAAP1
KLK7
PIGO
QRICH2
ACBD6
CEACAM19
CLSTN3
SPATA24
ABCA8B
AFG2B
APOA5
ATP5PF
CDSN
DEPDC1A
EEF1AKMT2
EFCAB6
FUCA1

GKN2
LMNTD2
LRTM1
MMEL1
PAX8
PROX1
STIM1
UBE2H
ZFP395
ARHGEF7
ARL6IP5
ARMC7
CALML4
CTDNEP1
NELL2
TUBGCP4
CYP1C1
DCUN1D4
EVI5L
NIPBL-DT
USH1G
CHST11
EPHA3
KLRB1B
KRTAP2-2
MAP6
SPARCL1
CSTDC4
IL17RB
COX6C
CRTAP
FOXO4
HRH3
LINC02875
LYZ2
NDUFA5
WWC1
ZC3HAV1
ZNF559
ARHGAP22
BOD1
CHI3L4
CRYZL2
GARIN4
IGKV5-43
IGKV6-23
PXMP2
RHCGL1
UGT5A2
AP1S2
ARRDC1-AS1

DNAH7A
DSCR10
ERN2
MEMO1
OR52K1
TEX19
ANO2
CSTDC5
EWSAT1
GNAT1
ICE1
LY6I
SPIC
STARD7-AS1
ZNF706
ATP5F1C
C17ORF107
CYP2B9
DNAI7
JAKMIP2
KBTBD12
KRT7-AS
MITD1
PABPC4L
PPP1R3E
SPTBN5
TRIM28
ZAN
ANO7
CGNL1
DLEU2L
LCE2A
MIRT2
MRGPRA2B
MYCBPAP
NAT14
NECAB3
PSPC1
RIT2
TFE3
TMEM222
DCC
GEMIN7
GPN3
MPLKIP
RRP36
SF3B1
TMEM26
LHFPL1
ZNF416
C8ORF88

CCS
MFSD6L
TEFM
BAZ1A
CASK
DSG1B
HIST1H2AO
KRTAP21-1
NWD2
RAI1
SERPINB3A
CPD
DLX6
RPS27A
EPHX4
ILK
SLC5A6
WDCP
WIF1
AIFM4
CMTR2
CSMD3
EMX1
AMY2A
ANTKMT
DDX19A
EBNA1BP2
FLT4
GPR34
H1F10
LRP4
LURAP1L
MMUT
PRXL2C
TAF8
TMEM128
ZMYM1
EPB42
FBXW9
PHF21B
TMEM179B
OXLD1
PPIL6
PRSS36
RPS19BP1
COX7C
GNA13
PLAAT3
SETBP1
DAP
SLC6A13

SIRPB1
SP8
ZBED1
MIR1268A
NEXN
OR10C1
POMK
PSG3
SFTA1P
A2ML1
CAPN12
CCSER1
KCNK9
KLHDC1
MPPED1
MPZL1
MUG2
SLC35D3
SYCE3
TPSAB1
WNT16
EMC10
FAM107B
HNRNPA0B
HSD11B1L
HSD17B12
IPO4
MTF1
MUC20
PYHIN1
TMEM181A
VIRMA
AARS2
DBT
FABP12
FBXO17
GAS2
INPP4B
PPP1R37
SIMC1
SPRN
STK39
THEM5
ZFP92
CCDC149
CYREN
JMJD4
KCTD4
KLRG2
MADCAM1
POU3F3

UBA52
VGLL1
RPN2
TMEM61
BOP1
KLRA9
MYLIPB
PNISR
RPL18
ADAT1
CCDC181
G6PC1A.1
LINC00900
MAN2A1
C3ORF52
DIDO1
AIG1
DKK4
MIR687
OR2F2
FOXD4
VGLL2
ACAT2
COMMD3-BMI1
CYTB
DACT1
DIS3L
ITSN1
ANAPC15
ENTR1
GNRH2
GTF3C3
KRT33B
NELFCD
PARL
PTCD2
TECPR2
TTC32
BTG4
C10ORF67
CASTOR1
CCDC22
DYNC2I1
EMC2
FAM182A
H3C7
IFI209
IGKV6-17
LRRN4CL
MIR23B
MROH7

SOX21
TAS1R2
TSEN34
TTC39D
ZER1
ZFP939
C11ORF71
C8ORF48
GRIP2
MORN1
OR4D11
REXO1L1P
TCTN2
AGPAT2
C1QTNF8
CIMIP2C
MIR3911
MIR7B
NSUN4
SLC13A2
SUOX
TEX55
TOLLIP-DT
ZNF295-AS1
CORO7-PAM16
DNAJC12
LATS2
LPXN
STX16-NPEPL1
STXBP1
C11ORF91
FAM86JP
HMGN3-AS1
ACOX1
NHLH1
ARL6IP1
CHTF8
EPHA6
GBP
HPDB
LDHD
NEUROD4
RPL8
ABHD5
FGF6
MLLT11
PNMA1
PPP3R1
SLC25A48
SRSF11
TLL2

ZNF22
CCR1L1
OR4K5
ORMDL1
PRL8A4
PSG23
SLCO1A5
SULT2A3
TRIM72
CARD14
COA6
DDRGK1
HSP-16.1
ULK3
HHATL
KLKB1
MED28
PLXNB3
TBC1D12
EDAR
ABCD4
DZIP1L
FANK1
MIR1468
NAV3
OOEP
PTDSS1
RPAP2
TSTD2
GPR37L1
SCG5
SLC7A14
ZNF500
CCDC152
DSG1
FTCD
MTERF4
SYNJ2
DCUN1D2
SNX10
XKRX
ZNF646
AARD
CLHC1
ERMAP
LRCH2
NHLH2
PRELID3A
RHPN1
SECTM1B
FOXD2

GK5
GOLGA1
MYRFL
BTLA
GFRA2
GNG11
PDIA5
PNPLA8
SCD2
SKIL
SLC22A23
TAX1BP3
TBC1D4
TRPM4
NT5C2L1
RNU2-10
ADAMDEC1
KRT16P2
MISP3
TLX2
TMEM161B
CEP72
CRISPLD1
FXYD4
HIVEP1
NEFL
TCHH
TMEM74
ABCE1
SLFN13
SMG1P3
UNK
ZMAT2
ZNF586
CCDC106
DBR1
H1F8
NUDT10
NXNL2
SLC26A7
TRMT11
VSIG10L
ZBTB39
CCDC71
CELA2A
DIRC1
FBXW2
H2AC14
LRRC75A
NATD1
PADI1

PDZD7
POMZP3
RAG2
SLC25A35
SRRM3
TGM4
C12ORF50
C12ORF76
DHRSX
GBP1P1
METTL23
SLC45A2
ST18
TAF1C
ZNF596
GAST
LIN7A
MIR1-1HG
PRDM10
ATP5F1EP2
CRHBP
FAM162A
ILDR1
LINC02910
MRLN
OAZ2B
ACNAT2
DEFB124
DYNLT1F
IGHV7-1
PAGR1A
PIGV
TNFRSF18
DBNDD1
LINC01132
MIR100HG
SNORA54
SNORD46
CEP295
COX17
EXOC3L2
ISPD
KIRREL2
LAMA4
LINC00652
PDE10A
ZNF891
OR52W1
SNX30
CAMLG
FCNA

FSIP1
MAP7D3
NDST3
PIRA12
SNAP91
STOML1
UNC80
VPREB3
WFDC18
CES4A
DNAJC17
PIANP
RIBC2
TMPRSS3
TTLL6
VTRNA2-1
ZKSCAN7
COCH
RAB30
CYB5A
EMB
IQCF3
OR13A1
SIM2
ZFP619
FIRRM
H2-T3
ZFP964
ZNF830
AIRE
MOBP
PDXDC2P-NPIPB14P
TAF5L
ZNF462
ACTN4
BEAN1
MPX
SLC6A19
SUSD5
CASS4
CPSF7
FAM118A
IFI30
KDM1B
LXN
THOC5
ABRAXAS1
BRAT1
BUD13
CDAN1
LILRA5

LYRM1
MS4A6B
RWDD2B
SLC41A1
SMG5
TBC1D10C
UBXN11
WDR89
CALY
COMMD6
GANC
MBL1
B3GALNT2
IGKV3-4
INTS9
PPP1R35
HSD17B6
PEAK1
FBXO40
KIN
TATDN3
MCPT1
PPP1R21
ACTR1B
C2ORF42
LIN28B
MIR622
OASL1
POLR3C
SNHG10
ST3GAL1
TMEM230
TP53RK
ZFAND1
AOX4
BRD3OS
COL26A1
COX20
FRY
HS6ST3
KANSL1L
MCRIP1
PIWIL2
PRRG2
PTN
RGS3
ANKRD20A8P
BICRAL
BMERB1
FAM50A
GMEB2

GPR171
KLHL15
KRT6C
MCAT
MPP3
MRPL44
NPHP3-ACAD11
PLEKHM3
RIPOR3
SLC36A2
SNX11
TMEM59L
TRAT1
ZC3H3
ZNF324
CHURC1
DCDC1
DUSP18
ETNK1
FAM131A
GUCY2F
METTL27
PCDH7
SMARCD1
SPCS1
SS18
VRK3
ARPIN-AP3S2
BLOC1S5-TXNDC5
CCM2L
FRZB
PEAK3
SLC8A2
CHKB-DT
ATP6V1A
CYP-35A2
FOXS1
FRG1
GPR3
SCN2B
UBE2D4
DESI1
RAB6C
SEPTIN3
SGCE
TDRD1
WDR35
CEP57L1
CYP4V2
H1-9P
IGKV8-24

LINC01270
NSUN6
RBM12B1
SPEF2
ARHGEF15
AS3MT
BBS12
F8A3
FAM149B1
H4C7
MIR298
MROH9
OBI1
SI
CCDC107
CFLAR-AS1
DCLRE1B
DHRS13
FTX
HAGHL
KRT2
LIPK
MILL2
MIR154
MYOT
PAXBP1
RPRD1B
TBP
CD82
DSPP
FAM170A
HMG20A
HYAL3
LRFN1
PEX11B
PPAP2A
RHBDL1
SHF
TMEM199
TMEM41A
TRIM45
TRMT9B
VWA5B2
CFHR3
KCNA4
SARS1
TIGD7
ZNF273
ZNF425
ZNF66
ZNF843

C1ORF159
EMC3
NDRG4
RNF169
SIGLECE
TPD52
ZNF141
PARP11
ADGRF1
ADPRM
ANKRD12
EIF1A
GALNT5
LIMD1
OR8J3
PICK1
RAB12
SENP5
TMEM170B
ZNF219
ANAPC16
PHYHIPL
RRAGB
TYW1
XLR4A
ALKAL2
GRAP
IDNK
LCE1G
SLC34A2
SNORD32A
TAPT1
DIMT1
GLIS1
LRGUK
PACRG
RFXAP
SIGLEC5
SLC39A3
SPINK5
ZDHHC16
CDKL3
GNAQ
PCNX2
SERPINA1A
XAF1
ACOX2
GPR137C
LRRN1
PALS1
PLA2G12A

RPL31
MPV17L2
SPX
CHMP5
FAM184B
MRRF
THOC7
ZNF805
AQP6
DICER1-AS1
GPR88
LCE3B
RWDD3
SERPINA3I
TJAP1
TMEM270
BEGAIN
CERCAM
DNAJB11
DNAJC22
FBXO28
HNRNPA3
LRRC8E
MEX3C
MORN4
NAT8F5
NXPH1
RHOT1
RIDA
TMEM131L
VSTM2L
ZC3H10
ABCF3
B4GAT1
CHCHD4
CLTRN
FOXD3
KLHL25
PIH1D1
THAP1
TMEM258
ATF7IP2
COMMD9
POTEM
NEXN-AS1
PFN2
WASH2P
ZDHHC24
ZFP523
NHSL2
VSTM4

ZNF165
AOAH
BTBD8
HAND1
HSP-16.2
NCAM2
NMRAL2P
OCRL
SLC51A
TENM1
TMEM132C
TNFRSF10C
ZNF517
AMBP
DDX60L
DNAH2
FAM217B
MSL3
MSTO1
PCYT1B
PLIN1
SCRN3
ZDHHC3
ZNF7
ASAP3
GABRB3
NTAN1
TTC33
BLOC1S4
CYP2C38
DYNLRB1
FBXO15
GPC5
IFI208
MICAL2
MTX3
MYG1
NCDN
PUS7L
SP6
SRBD1
VEZT
ZNF174
ZNF286B
CSPG5
DIPK1B
DNAJC4
H2AC7
KIAA0319L
RNU6ATAC
TNRC6C

B3GALT6
EXOC6B
SPINT4
SPMAP2L
TWSG1
LINC01255
LINC02372
MIR1249
PCBP3-AS1
TOX4
CRAMP1
HAPLN2
OR51S1
GGNBP2
HMGXB4
KLF1
LCN10
RNF187
ANAPC10
FCF1
GTF2E2
PCED1B
PHF20
RAB5IF
TM2D3
TNFRSF13B
UXT
ACP6
CHRAC1
DGCR2
ELFN1
FHIP1A
NSA2
PIGX
PSENEN
RETNLG
STARD7
TMCO6
TMEM198
APOBR
CCDC102A
CCDC51
CHMP1A
CLDN20
DGUOK
DOLK
FBXL17
GRM3
HSD3B
MED17
MIR511

ND4L
TATDN2
ZFP512B
LIN7B
LIN7C
SERPINB11
STYX
SYT4
TBCA
WHAMMP3
AK6
DOK6
EXOC5
INO80
SLC51B
SPATC1L
ZFP871
ZNF493
ZNF567
APOC1
DAND5
LAMTOR1
NAIP5
PPIP5K1
RAB11FIP5
SNORD104
TXLNGY
CCDC171
CHML
H2AC8
HEXIM2
NT5C3A
USP20
ATP5MK
BTBD2
CCDC138
COMMD4
CYTL1
EXOC3L4
GPD1B
IL20RA
OVCA2
PIGM
PLPPR3
ADAMTS3
ALDH5A1
BRMS1
CD244
CHCHD5
DRG1
FMC1

HPCA
KIF17
KY
LRRC39
MYH8
PLEKHA8
RASSF9
RNF183
SIX3
SSC5D
TTBK1
UBXN6
VPS11
ZNF335
GADD45BA
H2-EB1
H4C14
NKAP
TAF1A
EIF1B
WIPF2
CASP3A
TAB1
CBLN3
GSTM5
IQCC
SNCB
THOC6
ZNF217
CNIH3
EPM2A
ST8SIA5
TPTEP1
CCDC59
DDX28
POLA1
PRR5-ARHGAP8
RETREG3
TAMALIN
UBE3D
ZNF584
GTF2A2
PMEL
TCF15
TDRD6
TMEM69
ZFP36L2
APOL7E
CCDC127
CPTP
IFI206

KLRI2
RGPD3
SLC39A13
SSTY1
GPATCH8
LRRC45
RPS6KA3
IQCN
PITX1-AS1
RPS3A1
SNORD17
TMEM105
WDR87
BORCS8-MEF2B
LAMB4
ZCCHC8
ZNF749
DHX33
DLGAP3
GOLM2
HBB-B1
IPO11
MUP18
NPY2R
POLDIP2
SHISA3
SLC32A1
TYMSOS
DAPL1
ENDOV
ENTREP1
HDGFL2
IFT172
MRPS21
NPIPA1
PLGRKT
RPP25L
SLC24A2
USP46
XKR5
AGGF1
APOC4
CCDC81
CCSAP
CDC42BPG
ENGASE
IFT140
LAMTOR5
LRSAM1
MDFI
MGAT4D

RELL2
RESF1
RNF146
SCAI
TMEM92
WASHC4
ZNF485
ZNF677
ANKRD36
CCL25
IQCG
LIPT2
PNCK
RAB22A
SPDYE1
SPG21
KLHL34
LYSET
WDR92
ZFP120
FAM241A
IKZF4
MGA
SMARCD3
USP12
ANKRD39
COG3
CPNE6
FAM25A
FZD10
GABRG1
HECTD4
LINC02021
LINC02290
PPIAP46
RDH14
RPH3AL
SNORD62B
SYT3
UVRAG-DT
ARMC1
DNHD1
GRAMD4
IPP
LAYN
LYPD3
NAPG
PPIE
PSD4
SNX14
TINAG

TMEM130
TMX3
TRIM69
UNC13C
CDC23
EDEM2
EXOSC7
LCMT2
MBD5
NUDT2
NXPH4
TMEM160
ARPC1A
DEGS2
GBX2
MFSD4A
WDR83OS
HSP70L
PSG1
RNF225
STRIP2
ACTR6
ANKMY2
ASPSCR1
BRF2
DHRS1
HTR2C
RCBTB1
TNFB
CACHD1
CFAP91
CHCT1
COL22A1
CYP4A12A
LDLRAD3
PDE6C
PRDM8
PRPF31
BEND3
C1RL-AS1
CFAP99
CHMP2A
CRIPT
DDHD2
EIF4EBP3
H2BC10
KATNBL1
KCNA10
NGRN
RRAS2
USP32

ZFP773
ASB16-AS1
BFSP1
C19ORF33
HPS6
OR1F1
PGM1
RIC1
RPL26L1-AS1
SLC35B4
TMOD3
CCL21D
IFIT1BL1
KLRA23
RSPH3B
SH2D1B1
SNX13
SPEER4C1
USPL1
VSIG10
C1S2
CDC42EP5
CLDN10
DDX25
EVX2
FTH1P3
KCNS1
SKIV2L
SPANXD
CTSR
SCARNA9
SRSF7
TPBPA
ALPPL2
CARD8
GDF3
MCMBP
SPAG4
TCTA
ABHD14A
ANKS3
BDP1
EID2
FAM241B
FFAR4
FSHR
GOLT1A
LEFTY1
MED6
MPV17
MYH15

NGB
SMG7
SUMO3
TBL2
TNNI1
TTL
APOOL
BRWD3
FAM91A1
NR5A1
PALD1
RABAC1
THEM7
DTR
EXOSC3
GOLGA5
HAND2
PODXL2
PTP4A2
RNF2
APOL10B
SLC12A1
STK35
ZFP438
ZFP54
ALDOAA
MIPOL1
SPECC1
CC2D1B
CEP135
ENO1B
EXOC8
KLHL9
NKAIN1
NRDC
RTN4
SH3GLB2
TRMT2B
ZFP2
ANAPC4
CAB39L
D5ERTD605E
DBF4B
EIF3K
FOXF2
MED14
MSRB2
NANP
NEDD8
PPP1R26
RAD1

ZBTB7C
B4GALNT2
BCAN
C1QTNF5
CCDC125
CCDC157
CDX2
CPSF3
GGACT
HAPLN3
JOSD1
MATN3
RBP2
RTKN
SEC14L4
SNHG17
VPS16
BAZ2A
DGKI
FAM136A
GMEB1
HBD
LYSMD3
NDUFA8
SPTLC1
STK25
PLA2G3
BROX
CENPC1
CNTN4
POLR2J3
ALDOB
DCP2
MAP4K3
MRPL42
PHF8
RTP3
WASHC3
ZNF790
ANKS4B
ARSJ
ATP8B3
KRT76
MIR2682
MIR489
N6AMT1
POLR2C
PRAF2
RUNDC3B
SPAST
TMT1B

BCORL1
C1QTNF4
CACNG6
COA7
CR1L
DUSP26
GRIA4
HGFAC
MPHOSPH10
PAQR3
RC3H1
SERPINB6B
SZT2
TMEM252
UBIAD1
ZNF503
CCDC90B
DNAH7
HHAT
METTL16
SHLD1
THUMPD3
USP48
ZIC2
CHE-11
CLCA3A1
DHRS12
ELT-6
EPC-1
IFI213
IFI214
MOB4
NSMAF
OSM-3
OSM-6
PDK-1
PDYN
T28D6.4
TNFSFM13
TRIM34A
AGBL5
C2ORF49
MIR3675
PRSS33
TARM1
ZNF808
C16ORF54
CEACAM11
CYP4A3
GZMH
LCMT1

MAK
SMR3A
SNRNP200
ATOH7
DCAF4L1
DCP1A
KCNA1
SUSD1
TLE5
ULBP3
YIF1A
ZNF497
BRAP
DDX4
DHRS7B
DPH6
FITM1
GALR2
HIPK3
HNF4G
MRPS26
P3H4
PET117
PRMT2
RABGAP1
SERPINA3A
SMARCC2
SRL
URB2
WDTC1
YTHDC2
AKR1C18
BICD2
CCSER2
UBALD2
USP8
CDK2AP1
GORASP1
MARCHF6
PCDHGB5
RAD54L2
SFI1
ZNF490
ACCSL
C14ORF132
CASD1
EIF3CL
FAM20A
LCN3
LDLRAD2
LINC02256

MVB12B
PHKA1
SYNGR3
UBE2W
ZFP382
ZIK1
CAPRIN2
DLL3
FEM1C
MED12L
MYMK
SGCD
STOML2
ZMYM2
CENPP
DMRTA1
EFR3A
EIF3L
MRPL41
NDRG1B
POFUT2
SLC5A9
ZFAND6
AF357428
ARHGAP12
ATAD2B
C87198
CEP112OS1
ETFRF1
EXOSC6
GNPNAT1
LCE1K
LINC01070
LINC01874
MAGT1
MIR219C
MIR344D-2
MIR3969
MIR466K
MIR5108
MIR5120
MIR5619
MIR7649
NUP133
PCSK2OS2
PEAK1OS
SARNP
SIN3B
TIPRL
TMEM97
VMN2R75

ATP6V1F
CCNDBP1
COSMOC
CYB5R3
IL6R-AS1
LINC01271
LINC01990
LINC02029
LINC02175
LINC02709
LINC02783
MIR4745
MYO16-AS1
NIT2
OSER1
PGLS-DT
RANBP3-DT
RNF138
SBNO1
SCN1A-AS1
TOMM5
ZMAT4
ZNF292
C19ORF25
FAM177A1
SULT4A1
SYNCRIP
DDHD1
HOXD1
IL18BP
MIR1
PDZRN4
SMYD2
SNN
ZNF667
BABAM2
BBLN
DTX3
LINC02125
MIR556
MRTFA-AS1
PRKAR2A-AS1
SLC41A3
STAM
WNT1
BBS4
CATSPER3
CCDC47
CREBL2
KLHL23
NRBF2

PITRM1
PPP1R3G
THEM4
CSRNP2
FAM27E3
KRTAP2-4
NHLRC4
PMS2P5
RMDN1
ST20
BLTP3B
CEP290
CLK3
DRD3
KCNK6
LY6C2
SUN5
THNSL1
TTYH1
DCXR
H3C3
RBMXL2
ZNF445
DYNC1I1
ECE2
EMC1
MTERF3
XKR4
HSPB9
NEK9
NUP35
SCN11A
SIGIRR
SMIM22
SPNS3
STAMBP
NBPF11
PLA2G12B
PRELID2
RHOG
SLC31A1
SNAPC1
TSPAN31
UBA6
USP50
ADAMTS13
AFG3L2
AFM
C3ORF20
CLEC3A
CRTAC1

GOLGA8IP
HGSNAT
KCNH8
MACIR
MCPT8
MYT1
PPIL4
RNU11
TMEM150A
BNIP5
CFAP141
GK2
IK
KPRP
LHX8
OR51B4
RBM6
FOXO1A
HECTD2OS
MARS
OR2B11
TANC1
COX7A2L
CSTF2
OR1F19
OR51K2
VANGL2
ZFP746
ZFP758
ZFP949
ARHGAP31-AS1
C12ORF57
C17ORF113
C4ORF54
CEP170B
CNDP1
DACH1
GAS2L1
GRIA2
H2AC25
HEXA-AS1
LINC01275
LRFN3
MRPL46
MYRF-AS1
OSBPL11
PPP1R17
PYCR3
SLC18A1
SMC2-DT
ZNF652

GALNT16
MIS12
RSPO4
ZC3H8
CDHR1
MANEAL
PCMTD2
TBC1D8
UGT8
CARTPT
HAUS8
LTV1
PDE1B
PWWP4B
FHL1P1
LINC00307
NUP54
PRPF3
RLIM
TPTE2P3
DMTF1
FRMD1
GPAT4
PEX14
SP
SYNPO2
CDH17
IGL
IIGP1
MPHOSPH6
SEC11C
TM7SF3
U2AF1L4
UBE2D1
WDR88
ZFYVE21
AMHR2
DIO3
ELP2
EOLA1
GATAD2B
GPR39
GRM4
LONRF1
MIB1
PPDPF
ST6GALNAC3
ST6GALNAC4
TRIM8
ZFAS1
ZSCAN4

DPH5
GNG12
GNG13
PCDHGA6
POU3F1
PRORSD1P
TMEM63A
TRIM30B
WFDC6A
KLRG1
MILR1
ZNF491
ZNF837
BDH1
FOXR1
HTR5B
IL21R
METTL8
PMCH
SIGLEC10
ZNF135
ZNF700
CACNB2
CCNP
EID1
RTKN2
TTYH2
USP16
BSX
H2BC14
KIF4
MIR539
RBM4
ABCG8
PDCD2
RMND5A
TMEM62
TPM4A
ZNF737
ADGRA3
CLRN1
GFER
GNRH1
LCE3C
RGN
STK32B
SYT14
ZFP568
ERGIC3
GLUD1
IFNK

PGGT1B
RNF222
DPY30
HNRNPA0
KIF5A
NDUFB9
TMX4
TNFRSF4
TRIL
TWIST2
WRAP73
AKAP7
BTN3A1
C1ORF54
DLX1
LRRC63
SNTA1
TMED4
TPK1
UBE2M
UBR5
ZBTB33
ZNF367
CDCA4
GABARAPL2
SMN1
CEMP1
IFNA4
LHX1
ORC4
PARVA
VPS53
ARFGEF3
CARMIL3
DUSP9
GPHN
PEX1
PKP1
RGMB
RUVBL1-AS1
SLC7A3
SPAG7
KLF10
MTHFD2L
RIOK2
AK4
CEP85L
EME2
EPN3
PIP5K1B
SCN8A

SIL1
SLC9A7
TIMM23
CAMSAP2
CD1C
HNRNPCL1
PI3K92E
PTCHD3
TER94
ARL3
DECR1
MIR302B
PIRB
RAET1E
UBQLNL
APCDD1L
CRYBG2
LINC01587
LMO1
P2RX1
RGSL1
UGT2B4
CEP76
GCSH
GJD4
HS6ST2
NPHS2
NUDT22
SNORD89
ZNF788P
FBXL14
H2AC10
LINC02537
RAB1B
SPRED3
TMEM119
VAMP4
ARHGEF26
ELF5
FKBP2
GALNT1
PFDN2
TSPAN1
ZNF558
ZNF626
ALKBH4
ASIC2
EN2
KRTCAP2
NKX2-8
OXSR1

PTS
SLC1A4
ZNF430
CEP44
CYP2C11
ENTHD1
SMCO1
ZFP300
ARHGEF18
CHD8
CORIN
FHOD3
GTF3C2
KCNJ16
LY6C
OPN1MW
PEX13
TSSK2
TUBGCP3
BLCAP
C11ORF96
CCDC50
ISLR
MIR629
MTFP1
MTM1
OPHN1
S100A2
SENP7
ZBTB1
CORO1B
ESF1
GABARAP
POLR1A
SKIDA1
SMIM13
TRMT10C
USO1
FLT3L
SALL2
TMEM94
TMPO
UTP14C
ZFP37
CITED1
SLC26A5
TTC21A
VDAC3
AHI1
GPR32
MIRG

CHP1
CIR1
DTWD2
ITM2C
PARD3B
PNRC2
POLD2
UGT1A10
ALKAL1
KLK15
L2HGDH
METRN
MIR520A
PLEKHA7
STT3B
ZNF576
C5ORF46
CIB2
NENF
SETD4
SLAMF8
XCL1
ABHD1
SFRS3
SLC25A27
ZNF865
ATE1OSP
IGFBP1A
LINC00896
LINC01504
LINC01907
NCOA5
RASAL2-AS1
SMCO3
SNORD11
TMEM92-AS1
GSC2
HOXA10
L1CAM-AS1
TMEM141
UBL3
ART5
BCCIP
DENND1B
DPH1
EIF3J
MAN1A1
MRPL14
NECAB1
STOX2
ZC3H11A

ANKRD55
LANCL1
RAB11B
SCHIP1
ACE-2
ACE-3
CLINT1
CPOX
HECA
LHX2
PROK2
SULT2A8
CFAP276
GATA1
PRL3B1
ARHGAP17
CLP1
MIR33B
PGLYRP4
SH3YL1
TAF9B
ZNF593
ARHGAP35
CDK19
CERT1
DIP2C
G6PDX
GABRA5
ITSN2
KHK
MIR301A
RUFY3
SLC30A7
TTC17
LHX6
MMS19
NLRP7
PTPN7
SMIM34
TWF2
UBE2J1
UQCC2
ACSF3
FZD1
KLRA17
LRRN2
NECTIN1
NR2C2AP
NUP88
DMXL1
FAM163A

KBTBD3
MARK3
PANK3
PCDHGC4
ATAD3B
CDH8
DOCK6
FAM110B
KCNQ3
MIR487B
ZNF334
FAM210B
KCNA2
LSM5
POLG2
RPS2P32
TMEM19
AKR1C19
C1ORF50
FNDC4
GRAMD1C
H1-6
OR2W3
SEC61G
SSMEM1
TUBG2
USP53
ZNF510
PKD1L3
FBXL4
HELT
HTR6
SOWAHD
ZFP948
ACTL10
ANKRD18A
AVPR1B
DMAC2L
KCMF1
MORF4L2
RPP21
RT1-DMA
SLC17A2
CELF4
CUL2
CYP2C18
DIS3
EIF4H
MTARC2
NKX6-3
PIGA

RSF1
SERPINI2
ALG2
CAVIN3
NAE1
RAB18
WWP1
ACP36DE
CCN3
IFITM5
MIR486
MTH
SPACA9
TMEM14A
ZFP335
ZFP770
ZFP865
ZGLP1
DCAF11
GDE1
PDE6B
PRODH2
AKR7A3
ATP10D
MCOLN2
MIR3679
NAB1
OSM-5
RAB23
RGS-1
SART3
SCD5
SRGAP2C
SSBP3
TTC8
UCN3
ABAT
AGFG1
AKR1E1
CLEC16A
GLCE
HIST2H3A
ISCA1
N4BP2
NMRK1
TMEM38B
CALCOCO1
RTN3
UVRAG
EOLA2
HOXA6

MIR505
NUDT18
QRFPR
ZNF75A
H2-EB2
KIFC5B
MIR4742
PIK3R4
SDR9C7
SSTR2
TLK1
ZFP384
PRKY
STAG1
ZNF212
ALDOCB
FCRL6
LEAP2
LINC01443
MIOX
MPC2
PNPO
RAB4A
RNF112
SLC26A1
TEAD1
UNC93B1
ZNF358
ATP13A3
LINC01106
RAB11A
SH3GL1P1
SLC39A1
STRN3
WASH7P
COL2A1A
CRY5
DNM3OS
MCRS1
PROP1
TAK1
ZBTB14
ILP5
PCED1A
RAB40B
TMEM45A
ACMSD
DHH
GLIPR1L2
HOOK3
LIMD2

LRP2BP
NFE2L2B
SH2D1B
SMURF2
STK17A
TMEM117
ADAMTS7P1
ASTX6
ATP13A3-DT
CLDN34A
CNNM2
COX7C-PS1
GBP8
HDAC4-AS1
IRX3OS
JARID2-AS1
KCNJ8
KCNU1
LIF-AS2
LINC01460
LINC02043
LINC02289
MIR3474
MIR5093
MIR6239
MIR7043
MKX
NDUFV2-AS1
NEUROD6
NUS1
OLIG2
OR52B4I
RFX3-DT
RPL7A-PS8
RUFY1-AS1
SCART2
SDAD1P1
SLC6A19OS
TAS2R110
TMEM123-DT
TMEM185B
TVP23BOS
UNC45BOS
VMN1R148
YTHDF3-DT
CASC2
CUEDC2
FTHL17
KRT32
LINC01269
LINC01410

LINC02159
LINC02561
MIR3150BHG
OR7E47P
TBILA
WASHC2A
ZFP646
ZNF354B
CHD1
CHN1
FGF21
IKZF2
SLC35D1
SRGAP2
THPO
TOB1
ZC3H13
C15ORF39
FAM234A
JMY
MIEF2
MOGAT1
PCSK2
PDE6B-AS1
CNGB3
MT-RNR2
PCP4
SDR16C5
SPSB2
TMEM147
ADTRP
CCDC42
FAM89A
HMGN5
MBPA
MFSD2B
MNDAL
N4BP1
NAGK
RHNO1
ARHGAP18
CA7
GPR82
JPH2
PADI2
UBA2
ANXA2A
CCDC83
CDH7
CLDN17
LINC00174

ZNF766
ERI1
FAM135B
GOLGA2P5
NRTN
PCDHB8
SCN1A
THAP5
ADCK2
ADGRF3
ADH1B
LTBP3
MFSD14B
SPOCK1
SPRR2A3
STX12
ETNK2
H2BC3
RIPPLY1
TPTE
TTC13
UGT2B7
LINC02182
PEX19
PLA2G5
RT1-BB
ZNF253
CES2H
DENND4A
FA2H
MIR452
MUP21
NPC1L1
AAK1
ALPK2
LRRC37A6P
PGLYRP3
QPRT
SCN4A
SMPDL3B
UBR2
ZFP819
ZNF879
CRYGA
CSPRS
DAP3
FER1L4
GOLIM4
HSD3B5
JAM3
MEIS3

PPCS
PRPF40A
SCML1
SUSD6
VSTM2B
E2F5
ECEL1
TRAPPC2B
ZNF407
DCPS
GIPC2
H2BC5
LARP7
MSANTD1
RNF19A
ARHGEF19
CBFB
COX8A
CSDE1
IGLC2
SMCO4
ZNF486
ANXA1A
CCDC74B
CD226
FARP2
FIBCD1
MIR145A
NPEPPS
TRABD2B
EDN2
MAP3K2
PFKFB2
SIX1
CDC27
CRKL
EMC6
FGGY
HBE1
HINT3
PSMD13
RTL10
STOML3
TMEM134
TMEM215
TPPP2
ACVR2B
CATIP
DOCK11
EIF4E3
TAF7L

C7ORF57
STAT5
ACSL6
BRME1
CCL15
CRISP3
GPR179
HNRNPA1P51
LRAP
NDUFB4P12
NPIPB12
PPIAP78
RPLP0P5
RPS17P16
RPS3AP25
SNX5P2
SUN3
TPM3P8
KBTBD11
MIR340
NOL4
SERPINE3
AJM1
ANKS1
CYP3A57
EXD1
HNF1B
PPP1CC
PTPRT
SLC22A10
SLC6A17
TMEM240
HKDC1
IRX5
LINC00881
MRPL35
PLAAT4
PNKD
SMIM1
TRIM6
YKT6
ALPHA-EST3
CACNA1I
KRTAP27-1
LINC00616
LINC01484
LINC02541
MROCKI
SCAT8
SIRT4
ZXDA

AKR1B15
ARL2
MPZ
RNF170
BRMS1L
COL24A1
H1-1
ING3
PEX12
PPP2R2B
QTRT2
R3HDML
SMS
ZFP641
APOBEC3C
CYB5R1
GLYR1
HEPHL1
HIC1
KPNA1
PMM1
PMM2
RALB
RPL18A
SSXB10
ZNF524
AP3S2
AVIL
FGF19
SEPHS1
SLC35E4
TVP23C
ZNF223
ARID2
FXYD2
MED20
OPRL1
PGCP
SLFNL1
TMEM101
NOBOX
NPM2
SKOR1
BPIFB2
C11ORF52
CBX1
CTDSPL
NBPF3
RBM24
SLC1A6
STKLD1

ZNF506
CNGA3
CYP3A2
FBXO47
MIR136
POTEF
THAP2
TSACC
ZNG1A
ZRANB2
PTCRA
ZNF717
COL11A2
WDR26
ZNF48
CAMTA1
MAP7D2
H2AZ2
RRP1B
SGSM1
STXBP6
TMEM25
COQ5
GTF2B
SLC5A7
KLHL1
PITX1
SH2D6
SLC2A14
APBA2
C1ORF198
FER1L6
GCH2
MIR322
SLC12A8
LONRF2
SLC25A15
UPP2
DAF-6
DYF-7
MIR411
OGDH-1
ZFP799
LDHAL6B
METAP1
MIR4445
RPS6KC1
ARL2BP
ACT88F
BPHL
CASC9

NFYA
RAB9A
SNRPGP2
UGT1A6B
ZFP180
APITD1
KARS
MIR877
NANOS3
OLFR555
OLFR605
OLFR622
OLFR642
OLFR649
OLFR67
VMN1R171
WDR69
ETNPPL
IMP4
KCND2
MIR4484
MIR449B
NDUFAF1
ACIN1
H4C5
NDUFV3
SELENOW
C1GALT1C1L
CLEC19A
FAM72D
FKBP1B
GLIS3-AS1
KCNMB3
LINC00964
MIR3142HG
MIR760
MKNK1-AS1
NUP62
SCN10A
SCOC-AS1
SLC12A6
SPATA12
TMED5
TPTE2
ZNF513
CCND2B
CLPX
DPYS
EBLN2
EPIST
JAKMIP1

MIR647
RAB35
SERPINA3F
SUGP1
TMPRSS13B
DUSP14
PDX1
RXYLT1
TPP2
TULP1
URB1
ZNF615
C2ORF69
CES1D
OTOS
SPOCK3
ABF-2
HECTD1
NIPSNAP1
PTPRZ1
RABL3
ANP32B-PS1
AXUD1
C1QTNF9
C8A
CLEC18A
EXTL3-AS1
GPX2-PS1
HBQ1A
KIF23-AS1
KRT31
LINC01013
LINC01273
LINC02057
MIR1964
MIR19B-2
MIR3074-2
MIR3110
MIR6539
MIR669D
NICOL1
OR3A10
OR8J3C
PDE6G
RPL31-PS12
RPL32L
SAYSD1
SLC13A2OS
SULT3A1
TMEM9B-AS1
TNFRSF10A-DT

TUSC5
VMN1R211
VMN2R66
CNGA4
KCNG3
MCF2L2
NXT1
RBM4B
SEC23B
VPREB1
FAXDC2
GPATCH2
KCNH1
LYPD6
MIR296
OXGR1
PCDHB11
SCAF8
ZFP329
IGKV1-117
MIR1207
MIR4750
MIR691
RPL14
SLC3A1
SNAR-A3
SPAG17
TCP11
XKR7
ANO10
C16ORF87
DPY19L3
EFCAB12
FRG1-DT
GULO
PSMD6-AS2
ADH1
MRPS36
WAC
MIR1183
ASF1A
BTNL8
NACP60E
RGMA
SENP17
ZNF547
MACROD2
MS4A10
NIBAN1
RRAGC
RWDD4

SH3GLB1
SLC16A13
ZNF621
APOBEC3F
B3GNT4
DPEP3
DSCAML1
GPX1P1
H3P16
IGHV1OR21-1
IZUMO1R
MIR337
NBL1
PPM1K
RPL15P20
RPL17P7
RPL21P16
RPL23P6
RPL4P5
RPS2P20
SRP9P1
TTC7
ZFP750
FAM186A
FOXD4L4
HSD52
LINC00518
PFKFB1
ACP2
BAXA
KIF13B
MACROH2A1
MIRLET7G
PAQR7
RPL6P27
ZNF268
MTNAP1
ZNF605
APOL6
ESPNL
LY6G6D
NUGGC
TRDMT1
UMAD1
VWA5B1
ZFP275
ZNF550
ZNF587
DNAH17
NKAPD1
ILP2

NDUFA2
REPS2
SIGLEC11
SNORD63
WASIR2
ZNF337
ZNF890P
ISX
KRT27
ADAMTS6
KLRA7
LIX1L
MRGPRE
SELENOK
ATP6V0A2
C1ORF141
CATSPER4
MARCHF3
NUP153
PDP2
SDK2
ADIG
ARSL
ARX
JPT1
MFRP
NANOS2
RPL7A
ZFP444
ACOT11
CADPS
KCNQ2
M6PR
PPIL3
TMED10
TNFSF8
EAF2
MCPT2
ZNF597
OTULIN
STEEP1
ZC3H7B
ZNF551
ZNF660
BRIX1
NR2F6
PRR15
USP28
ZFP707
CIMAP1C
GCKR

GSTE8
GYG1
LDLRAD1
LINC01605
MARCHF9
PPP4R1L-PS
SLC2A11
AVEN
C3ORF38
GNB5
H2-DMB2
IRGC
KDM7A
DAD1
ETV7
PEMT
C8B
DHRS2
DTX1
KPNA7
MBOAT4
SSBP2
CXCL8B.1
DRGX
FOXE3
PGM2L1
SSTR1
GABRG2
GNG7
LMAN1L
PAQR9
BPNT1
CD300E
COL-80
H2BC4
HTR1F
KLRC3
MAGI1
MIR3152
MMP27
PYGMA
RPL4
WDR53
ZFP69B
ZNF235
ANG2
C10ORF53
HBBE1.3
KRT12
LCORL
RAB27B

RSU1
SIRPB2
SNORD37
UBAP1
ZNF234
ALPHA-EST2
ANKRD53
BRICD5
ERDR1X
KCNIP4
KRT85
SLC15A1
TADA2B
TRAPPC14
ABCG5
AKR1C8
ARHGEF26-AS1
DCAF1
DHRS4L1
GBP9
IL31
PCDHGB3
PLPP5
PRDM7
RPL34-DT
UGT2B11
CD209F
GAPT
KRT42
MIR4423
SLURP1
ZNF600
GSTE4
PRRC1
SHANK1
WFDC6
BSK
CBR3-AS1
GHRLOS
LIPN
NPPA-AS1
PRDM10-DT
PTCSC2
TBC1D3C
UBR5-DT
ZNF282
AADAT
IFI27L2B
IGBP1P1
LYZL4
NOB1

SNORD116-28
ZNF271
BBX
DESI2
MIR559
REG1
RNF6
RPL28
SLC35E2B
VSTM2A
IGKV3-2
VMAC
BRWD1-AS2
CCDC129
CTSML1
DRD1B
EIF4A3
FBXW4P1
FRS30S
GOLGA8R
GSTA5
GTDC1
H2-Q7-PS1
HMOX2-PS1
HOXB-AS1
IGD
IL12A-AS1
LINC01629
MT1HL1
OR2Y6
OR8G4
PAPLN
RPL19L1
TMEM181C-PS
WWTR1-AS1
BCAT2
CHCHD2P6
EEF1A1P11
FOXH1
LINC01205
LYPD2
NAA15
RPL15P17
RPL19P9
RPL21P119
RPL36AP37
RPL7P59
RPLP0P10
RPS12P22
RPS3AP47
SDHDP2

SIP1
ABCA15P
BCL7A
CDRT15P3
ERAP1
H2BC17
KLRA22
METTL24
MROH2A
NTAQ1
RPL24
ZFP592
CYP2D3
LINC02245
RASL11B
SNORA36A
TPH2
WAKMAR2
AANAT
ATP6AP1-DT
KLRA1
LDHAL6A
MALINC1
MIR3940
NDUFAF5
PLAC8L1
PLEKHH2
SNORD110
ZFR
CHK-1
ND1
UPB1
AFMID
C1ORF52
CGB3
NBPF25P
NT5C1A
OAF
ADHFE1
CAPN11
HSP27
MIR345
TSSK6
UTY
ZDHHC14
ZFP609
CACUL1
CDHR4
FCNB
RTF2
AFAP1-AS1

DRC7
KIR3DX1
MIR224
PDF
SCG3
SERPINA1B
SIGLEC16
SMIM10
SRD5A2
TEX12
CHN2
IGHV1-36
IGHV3-1
NIPAL2
TRBV17
AARSD1
AGPAT5
CRISP2
EPPIN
GYS2
KCNAB3
KCNE5
LINC00261
MIR139
NAIP3-PS1
NKX1-2
RASL2-9
RPL38
ZFP715
ZNF260
CLEC12B
F8A1
KCNA7
LAMTOR2
CAR6
FSCN3
NPL
RAB17
BLZF1
LILRA6
REDIC1
RNF151
SLC38A3
HSP-3
SNRPA1
TMEM200B
TP53I13
UTF1
ZSCAN10
ACANA
LAMC3

MROH8
PVRL1
SCARNA17
SCNM1
ZNF37BP
GSTE2
KIAA0825
FAM65A
LINC00622
SEPT2
SEPT6
TNFRSF17
VWA3B
ZNF800
CACNG3
GON7
HIGD1B
KCNG1
KDF1
MIR215
MIR3175
RLIG1
SHHA
TMEM198B
PCOLCE
ZFP874B
B4GALT6
CKLF
CUBN
FBXO24
HDHD5
HSPA13
KIAA0232
PCDHB2
SNORA80E
ZNF592
DRS
FMO-2
C9ORF40
CNOT10
KPTN
NOX3
RARS
TBRG4
UNC13B
C5ORF58
CCNB3
PCP2
RHOQ
ZNF287
CENPC

NCS1
SNORA71A
SNORD12
SNORD71
ZNF226
ZNF318
ZNF746
ABRA
CCDC177
GAGE12J
NDUFA9
PAIP1
PMFBP1
TXNDC8
ZNF330
MIR3178
MIR378E
ASS1P11
ATP6V1G3
EEF1A1P22
KIF19
MOV10L1
NAB2
NCMAP
RPL12P32
RPL29P11
RPS26P15
RPS26P2
RPS29P9
SNRPGP15
SUN2
TCTE1
XRCC6P1
CTC-338M12.4
GOLGA8K
H3-3A
HAO2
KRT71
LINC00398
LINC01424
LMCD1-AS1
MEI1
MIR1229
MIR4449
MIR525
MIR548N
MIR548V
MIR7111
PA2G4P4
PITPNA-AS1
SNORD103C

SNORD14C
ZFP385A
ZNF687-AS1
HPSE2
ZSCAN5A
ASB18
BTN3A3
GAGE12B
GOLGA6L22
KCNG4
KLF17
KRTAP9-7
LINC01620
OR7E125P
RELCH
SGPP1
UGT1A9
ASB10
LDOC1
SLAMF6
ALDH1A3-AS1
ANP32E
ATP13A5
ATP5S
CD74A
CGM4
CTSQL2
DCST1-AS1
DRD2A
IL11A
KIR2DL5A
LHX4-AS1
MIA2-AS1
MINCR
OR52N2B
PPP1R2-PS3
QPCTL
RBM14-RBM4
RBPMS-AS1
RNF213-AS1
RPL13P5
SCIRT
SLC34A3
SRRM2-AS1
TNFRSF14-AS1
UGDH-AS1
VMN1R205
VMN2R58
VOM2R52
ZNF710-AS1
CFAP144

GPR156
IGHV1-20
IGKV3-5
IRX6
PDE1C
RPS20
ZNF606
ART1
FAM74A3
FER1L6-AS2
FOXB1
KEAP1A
KRT16P3
MIR645
NRL
TLX1
ZNF6
CCDC170
CIMIP6
GLOD5
GOLGA6L6
IGHV1-18
SLC25A34-AS1
TTC5
ZNF84
FTMT
GPATCH1
MIR3588
VASH1-AS1
ZNF512B
FAM27B
HR96
RAB19
VOM2R37
ZNF654
ACTL6B
EVX1
GASK1A
MALT1-AS1
PACRG-AS3
C12ORF73
GDAP5
H1-0
OGFOD2
PANK1
RFLNB
TMEM8A
VMN1R17
VMN1R188
BCL2A1D
BHLHB9

CNTFR-AS1
MIR455
NDUFAF4
PLDI
PVALB
TBCCD1
TSTD3
ANGEL1
ANKRD35
KRT25
ACTR5
KLRA10
MIR28A
RPUSD1
TFAMP1
UBASH3A
ZFP462
CASP3B
GDPGP1
H2BC12
KLHL20
LINC01996
LIPT1
LURAP1
PCBD1
RPS11
SARDH
SNORC
ZFP157
ZSCAN25
CFAP263
PRL3D3
SPEF1
ARHGEF4
ASPN
HAAO
HEMGN
MIR324
MIR362
PRM2
SMIM19
HSDL2
KGD4
NBPF1
SSPN
CARNS1
H2-M10.1
H4C12
LHX5
MIR6775
NMUR1

SFT2D3
TMEM186
CNTN6
COL-19
FNDC10
MIR651
SNORD38A
UGT2B34
VWC2L
AATBC
CRNDE
KRT81
MIR181C
MIR2116
ODF3B
PAX1
SHISAL2A
SLC7A11-AS1
DRE-MIR-27E
HMGB1P10
KRT18P31
MICOS13
MIR142HG
MNX1
NPIPB8
SAMD4
H2AC1
H2-D1
HSD17B11
LINC01551
LY6H
OTUD7A
PALM3
TRIM68
AQP12A
ATP5MGL
CD1D
PRAP1
SLC9A4
CSTPP1
IFNA2
MIR4508
SLC38A11
SMDT1
ARMCX5
C21ORF91
H1F5
P4HA2
PIGG
FAM86B2
MUP2

TUBB2C
C2CD4D-AS1
KCNJ3
MIR1469
OAZ3
PAN3-AS1
PRR7-AS1
RNU5B-1
SNORA57
UCKL1-AS1
FRS3
GAR1
GSTP3
LYSMD4
ABRAXAS2
CEP104
CYT1
FSCN2
LINS1
MEIOC
RNF215
RPF1
SRRD
ABHD6
HTR3D
SULT2ST3
USP32P1
ZNF365
CIMIP5
DOS
FAM188B
GLTPD2
GOLGA8H
GSDMC2
IFI202B
LINC00205
LINC01191
MIR16
MIR3060
MIR7654
NUDT16L2P
OR7A35
OR8G34
RB1-DT
RPS15A-PS6
SPEER3
VAX2
ZFP804B
ARL16
CACNG5
ENTPD6

SPATA45
ST7-AS1
STARD4-AS1
TPGS1
TUBA3FP
ARL14EP
GIMAP7
MIGA2
ATP1A4
CHST5
FAM83E
H2AC12
LIM2
LINC01564
NSUN3
RNF145
RUNDC1
SNAR-B2
SNAR-D
CFAP96
FAM151A
NLRP1B
ZNF337-AS1
ATP6V1E2
CYP4A12B
HBG2
TVP23A
ZSCAN5B
CHIA.3
CYP2D10
INSYN2A
LYRM4
PROB1
TAFAZZIN
TDRD9
ADH4
AGPAT9
BORCS7
CFAP100
CHGB
ISM2
S100A5
TMEM217
BTNL3
CCR10
CDH12
CXORF65
HIGD2A
MRPL43
MTARC1
SMIM32

ACER1
ATP1B4
POLR3G
CIAO2B
LPCAT3
METTL26
POLR2J
AMY1B
CCT6P3
CHD9
DELE1
FMOD
GSG1
KCNK4
LINC00938
MRPL10
MTND5P10
NLRC3
OPTC
PSAT1P3
RNU6-15P
RPL10P9
STUM
DECAY
GST-10
PIP
PNPLA4
SFR1
SLC4A1AP
SLC5A10
STAI
DNAH3
FAM83B
FBXO45
FH1
RNF25
SAMD10
WDR24
KIFBP
HSPB11
LRFN5
TMEM248
ALX1
ATPAF2
COPS6
H4C2
IL31RA
PEX10
RNF113A
DPM2
MTRF1L

MYOZ2A
FAM78B
PROSER2-AS1
RHOX5
RTL6
SEBOX
ANKRD49
BCAS4
CFAP97
CIRBP-AS1
LGALS16
NKX2.5
SNORD118
TPTE2P1
USP30
XKR6
ZC4H2
CCDC43
HENMT1
MIR9-3HG
MTG1
MGAT4C
TCP10B
TMED6
TOMM70
APL-1
BEAN1-AS1
FAM149B
MORF4L2-AS1
MUP1
SLC25A10
C12ORF54
C9ORF163
CEACAM12
CIMIP1
FGD5-AS1
LINC00106
LINC00589
LINC00607
MIR1296
MIR466H
MIR677
MIR700
OR10D5J
PELATON
PIGY
PRL3D4
PRRG3
RSPH10B2
SNORD33
TEN1-CDK3

ZFP408
AP4S1
KLHL8
NOM1
ZMAT5
ADH5P4
ALG11
BRK1
CCDC85C
CRIM1-DT
CYP2K6
ITPR1-DT
LINC00484
LINC01635
LINC01686
MIR193BHG
MIR6730
NAMA
NOS
PRAMEF22
RHD
ZNF750
ARSD
DRICH1
IGLC1
KIAA1549
SNAR-G2
SNAR-H
TMEM242
C1QTNF12
KIAA1328
LINC01583
LINC02912
METAP1D
MIR3960
MMP25-AS1
OAS1B
SMIM14
TBC1D3P2
ZDHHC1
ZNF724
ADK
AQP3B
CCDC33
CDK3
FAM76A
FUT10
KCTD21
RBM27
HMG20B
KLHDC7B

LINC01160
OR7E170
RPL17P33
STK4-DT
TRMT112
CCDC163
ERV3-1
H4C11
MIR4505
NKAIN2
