# Supplementary material for: Bioinformatics Approach to Identifying Molecular Targets of Isoliquiritigenin Affecting Chronic Obstructive Pulmonary Disease: A Machine Learning Pharmacology Study
Source: Int J Mol Sci. 2025 Apr 21;26(8):3907. doi: 10.3390/ijms26083907 (PMC12027559; doi:10.3390/ijms26083907)
Supplement: Supplementary file 1 [file ijms-26-03907-s001.zip › tableS3.pdf]

Table S3 Drug target gene lists obtained from databases

CTD[13]	Pubchem[16]	TCMSP[15]
AKT1	BACE1	VCAM1
CASP3	JUN	JAK2
MMP2	USP1	TYR
MMP9	CYP3A4	NA
CASP9	MAPT	PIM1
INS1	KCNH2	FOS
CYP1B1	IDH1	PTGS2
NOS2	CYP2C19	PTGS1
PTGS2	VDR	PIK3CG
SLCO1B1	GBA1	PPARG
TNF	RELA	NCOA2
CYP4A11	GMNN	NOS2
IL6	ATAD5	PRKACA
RELA	NFKBIA	MAPK14
BAX	NOS2	MT2A
BCL2L1	NOS2	F11R
CAT	NOS2	HSP90AA1
CYP1A1	NOS2	GSK3B
CYP1A2	nos2a	GABBR1
IFNG	NQO1	ESR2
NFKBIA	NR1I2	ESR1
ARG1	PARP1	SELE
CCKAR	PDK1	CCNA2
CDKN1A	PLA2G5	CDK2
CYP2E1	POU5F1	CA2
HSPA5	PPARG	PKIA
IL1RN	PTGER4	ampC
ITGAM	PTGS1	ADRB2
MELK	PTGS2	BAX
MKI67	PTGS2	AR
MRC1	PTGS2	MAOB
NQO1	PTPN1	
NR1I2	RELA	
PARP1	RELA	
PTGER4	RELA	
SLC2A1	SIRT1	
SOD2	SLC2A1	
AFP	SLC2A4	
AHR	SLC6A3	
BCL2	SLCO1B1	
BIRC2	SOD2	
BIRC3	SOD2	
CASP8	STAR	
CCND2	STAT3	
CCNE2	TGFB1	
CDKN1B	TICAM1	
CDKN1C	TNF	
CEBPA	TNF	
CHIL3	TNF	

CHUK	TNF
CTSB	tnfa
CXCL10	TRAF6
CXCL18B	Trp53
CXCL8A	UGT1A1
CYP17A1	UGT1A10
CYP19A1	UGT1A7
CYP2C19	UGT1A8
CYP2C9	UGT1A9
CYP3A4	XBP1
DDIT3	AFP
ERN1	AHR
FABP4	AKT1
FOXO3	AKT1
GADD45GIP1	AKT1
GFAP	AKT1
GPT	ARG1
GPX1	BAX
GSR	BAX
HAVCR1	BCL2
HMOX1	BCL2L1
HSD17B1	BCL2L1
HSP90B1	BCL2L1
IL10	BIRC2
IL4	BIRC3
INSR	CASP3
IRAK4	CASP3
MAP1LC3B	CASP3
MAPK1	CASP3
MAPK3	CASP3
MFN2	CASP8
MYD88	CASP9
NFE2L2	CASP9
NFKB1	CASP9
NOS2A	CASP9
PDK1	CAT
PLA2G5	CAT
POU5F1	CAT
PPARG	HMOX1
PTGS1	CCKAR
PTPN1	CCND2
SIRT1	CCNE2
SLC2A4	CDKN1A
SLC6A3	CDKN1B
STAR	CDKN1C
STAT3	CEBPA
TGFB1	Chil3
TICAM1	CHUK
TNFA	CTSB
TRAF6	CXCL10
TRP53	cxcl18b

UGT1A1	cxcl8a
UGT1A10	CYP17A1
UGT1A7	CYP19A1
UGT1A8	CYP1A1
UGT1A9	CYP1A2
XBP1	CYP1A2
	CYP1B1
	CYP1B1
	CYP2C19
	CYP2C9
	CYP2E1
	CYP3A4
	CYP4A11
	CYP4A11
	DDIT3
	ERN1
	FABP4
	FOXO3
	GADD45GIP1
	GFAP
	GPT
	GPX1
	GSR
	HAVCR1
	HSD17B1
	HSP90B1
	HSPA5
	IFNG
	IFNG
	IFNG
	IL10
	IL1RN
	IL4
	IL6
	IL6
	Ins1
	INSR
	IRAK4
	ITGAM
	MAP1LC3B
	MAPK1
	MAPK3
	MELK
	MFN2
	MKI67
	PTGS2
	MMP2
	MMP9
	MRC1
	MYD88
	NFE2L2

NFKB1

NFKBIA
