# Supplementary material for: Bioinformatics Approach to Identifying Molecular Targets of Isoliquiritigenin Affecting Chronic Obstructive Pulmonary Disease: A Machine Learning Pharmacology Study
Source: Int J Mol Sci. 2025 Apr 21;26(8):3907. doi: 10.3390/ijms26083907 (PMC12027559; doi:10.3390/ijms26083907)
Supplement: Supplementary file 1 [file ijms-26-03907-s001.zip › TableS4.pdf]

Table S4 Demographic characteristics of the study population in dataset GSE130928

Parameter	Nonsmokers	COPD	COPD vs. Nonsmokers *P* Value
Transcriptome cohort, *n*	24	22	
Sex, M/F, *n*	18/6	18/4	0.5 (0.62)
Age, yr	40 ± 8	54 ± 8	<0.001 (<0.001)
Race, B/W/H, *n*	15/6/3	5/13/4	0.05 (0.97)
Smoking, pack-years	—	43 ± 28	—
FEV_1	102 ± 12	85 ± 23	0.001 (<0.001)
DLCO	81 ± 6	62 ± 8	<0.001 (<0.001)
GOLD stage, I/II/III/IV[‖]	—	13/7/2/0	—

Definition of abbreviations: COPD, chronic obstructive pulmonary disease; B, black; H, Hispanic;W, white; FEV_1, forced expiratory volume in 1 sec; DLCO, diffusing lung capacity for carbon monoxide; GOLD, Global Initiative for Chronic Obstructive Lung Disease.
Demographic data are presented as mean ± SD unless otherwise specified.

[‖] Based on GOLD (the Global Initiative for Chronic Obstructive Lung Disease) criteria.

The above table data and content are from the published literature:O'Beirne SL, Kikkers SA, Oromendia C, et al. Alveolar Macrophage Immunometabolism and Lung Function Impairment in Smoking and Chronic Obstructive Pulmonary Disease. Am J Respir Crit Care Med. 2020;201(6):735-739. doi:10.1164/rccm.201908-1683LE
