# Supplementary material for: Bioinformatics Approach to Identifying Molecular Targets of Isoliquiritigenin Affecting Chronic Obstructive Pulmonary Disease: A Machine Learning Pharmacology Study
Source: Int J Mol Sci. 2025 Apr 21;26(8):3907. doi: 10.3390/ijms26083907 (PMC12027559; doi:10.3390/ijms26083907)
Supplement: Supplementary file 1 [file ijms-26-03907-s001.zip › TableS5.pdf]

Table S5 Demographic characteristics of the study population in dataset GSE13896

Parameter	Healthy nonsmokers	COPD smokers
Number	24	12
Sex (male / female)	18/6	10/2
Age (yr)	40.3 ± 8.2	54.7 ± 9.3*
Ancestry (B/W/H)[1]	15/6/3	1/8/3
Smoking history (pack-yr)	0	49.8 ± 28.3
Urine nicotine (ng/ml)	negative	1217 ± 1301
Urine cotinine	negative	1243 ± 639
Venous carboxyhemoglobin (%)[2]	0.5 ± 0.8	3.2 ± 2.1
Pulmonary function[3]		
FVC	106.1 ± 11.2	105.2 ± 24.6
FEV1	102.9 ± 11.8	85.2 ± 22.9*
FEV1/FVC	81.5 ± 4.7	64.2 ± 4.4*
TLC	96.8 ± 8.1	112.8 ± 21.6*
DLCO	94.3 ± 8.5	75.4 ± 17.0*
GOLD Stage (I/II/III)[4]	--	8/3/1

All data are mean ± standard deviation.

[1]B, black; W, white; H, hispanic.

[2]Venous carboxyhemoglobin, a secondary marker of current smoking; normal level for nonsmokers <1.5%.

[3]FVC - forced vital capacity; FEV1- forced expiratory volume in 1 sec; DLCO - diffusing lung capacity for carbon monoxide; TLC - total lung capacity; all values are presented as % predicted except for FEV1/FVC presented as % observed.

[4]COPD staging defined by the GOLD (the Global Initiative for Chronic Obstructive Lung Disease) criteria.

*p<0.05 as compared to healthy nonsmokers.

The above table data and content are from the published literature:Shaykhiev R, Krause A, Salit J, et al. Smoking-dependent reprogramming of alveolar macrophage polarization: implication for pathogenesis of chronic obstructive pulmonary disease. J Immunol. 2009;183(4):2867-2883. doi:10.4049/jimmunol.0900473
