# Supplementary figures and images for: Bioinformatics Approach to Identifying Molecular Targets of Isoliquiritigenin Affecting Chronic Obstructive Pulmonary Disease: A Machine Learning Pharmacology Study
Source: Int J Mol Sci. 2025 Apr 21;26(8):3907. doi: 10.3390/ijms26083907 (PMC12027559; doi:10.3390/ijms26083907)

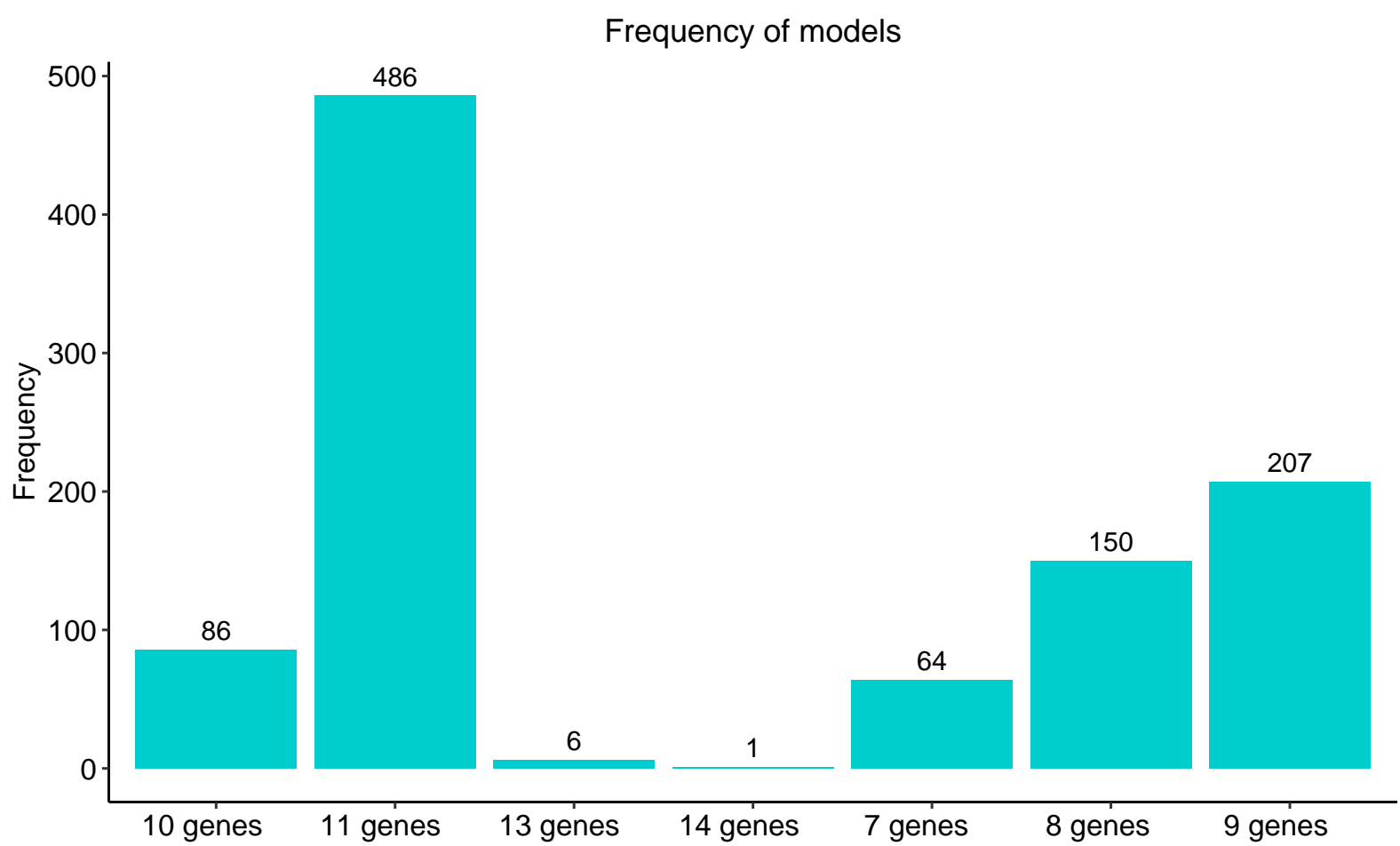

Figure S1 Lasso screening diagram

Supplement: Supplementary file 1 [file ijms-26-03907-s001.zip › figureS1-LASSO.pdf]

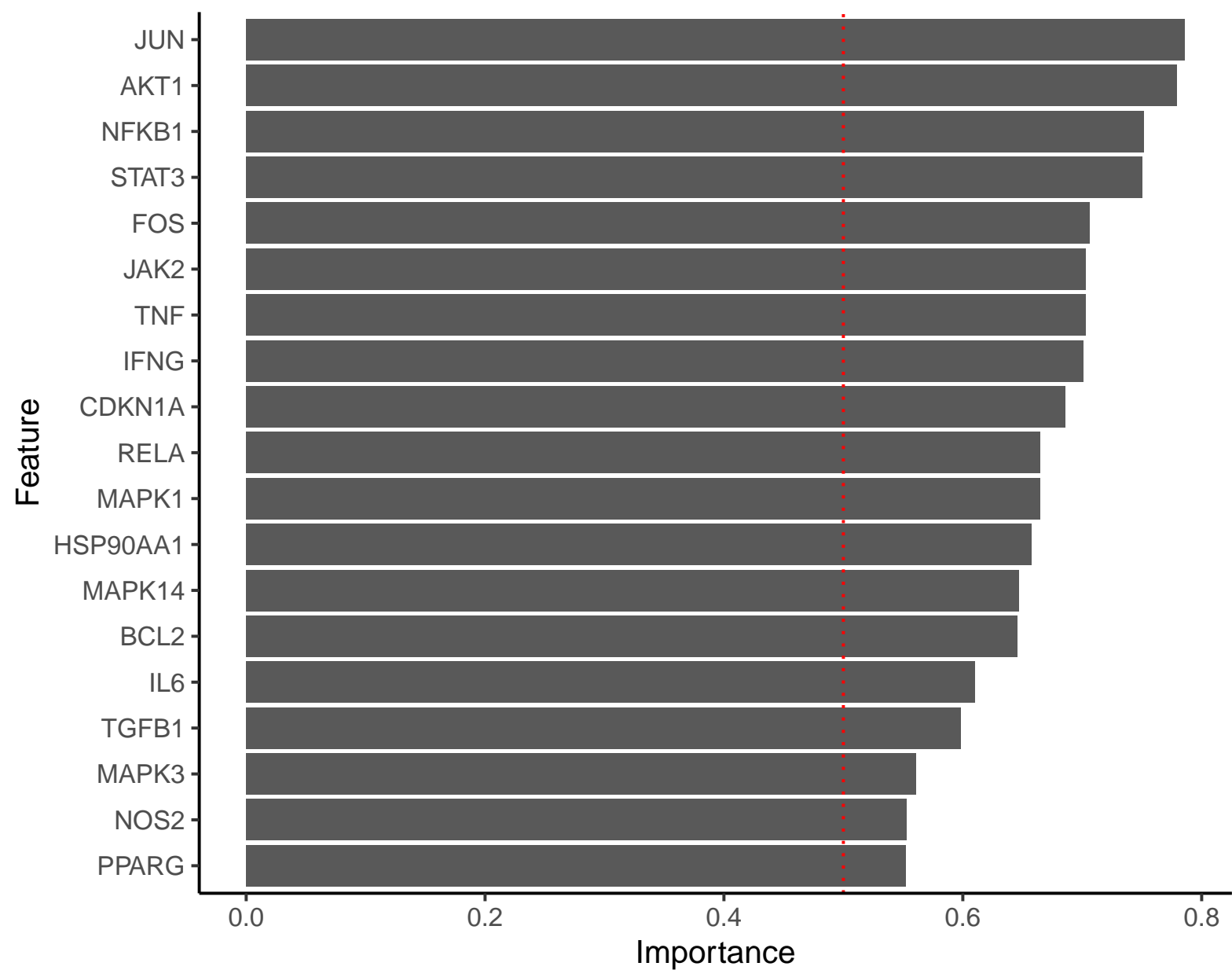

Figure S2 LVQ screening diagram

Supplement: Supplementary file 1 [file ijms-26-03907-s001.zip › figureS2-LVQ.pdf]

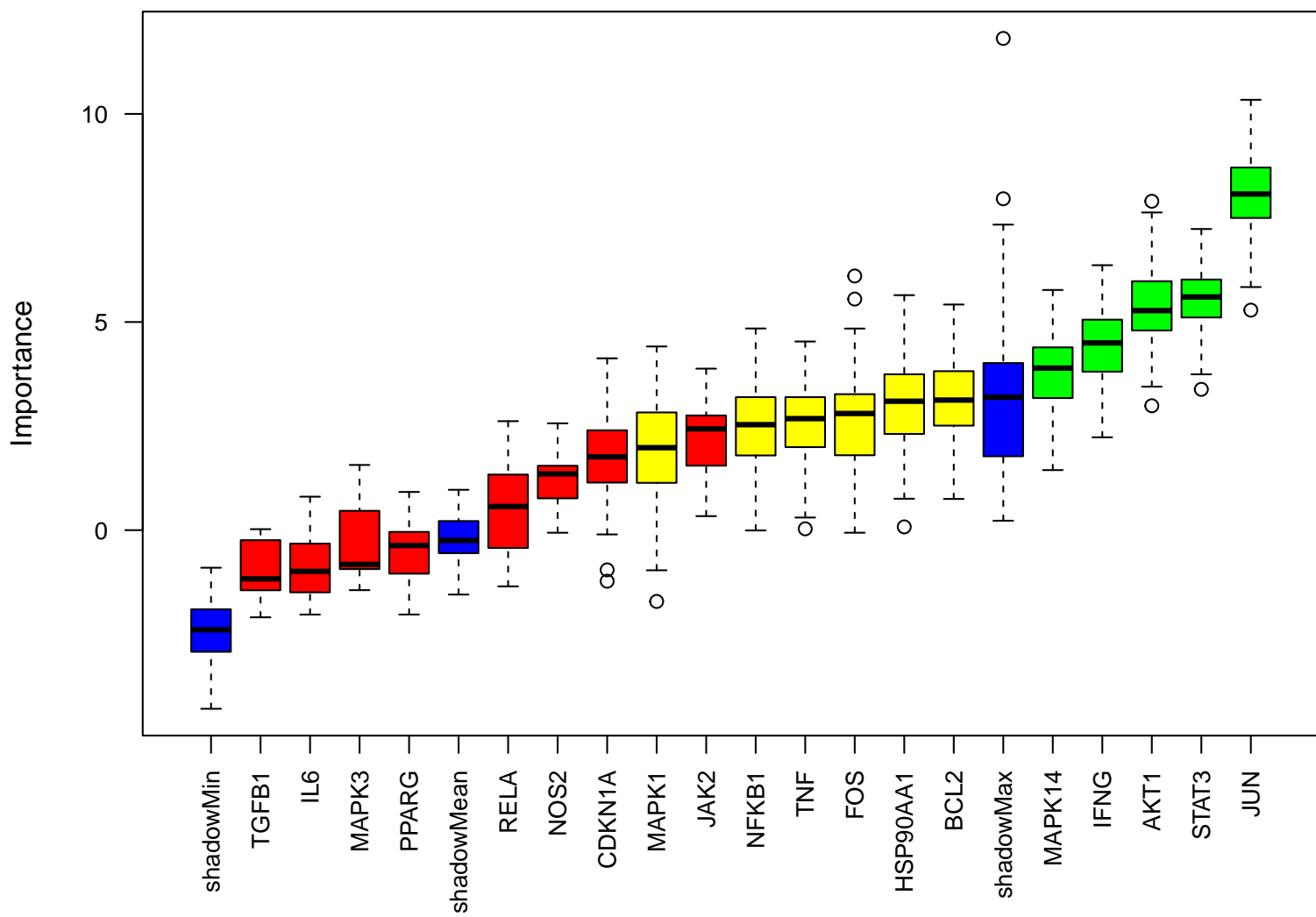

Figure S3 Boruta screening diagram

Supplement: Supplementary file 1 [file ijms-26-03907-s001.zip › figureS3-boruta.pdf]

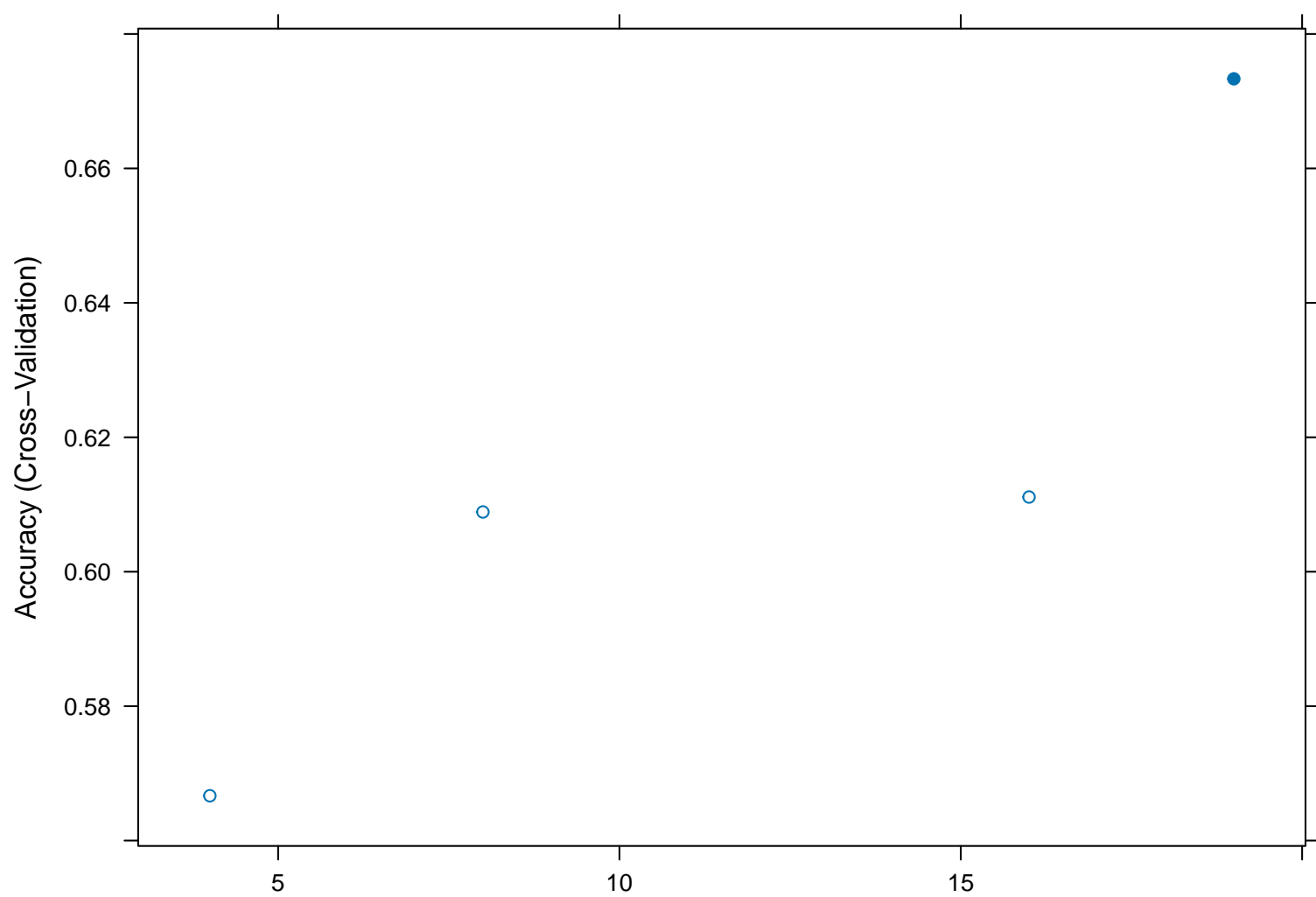

Figure S4 Bagged Tree screening diagram

Supplement: Supplementary file 1 [file ijms-26-03907-s001.zip › figureS4-treebag.pdf]

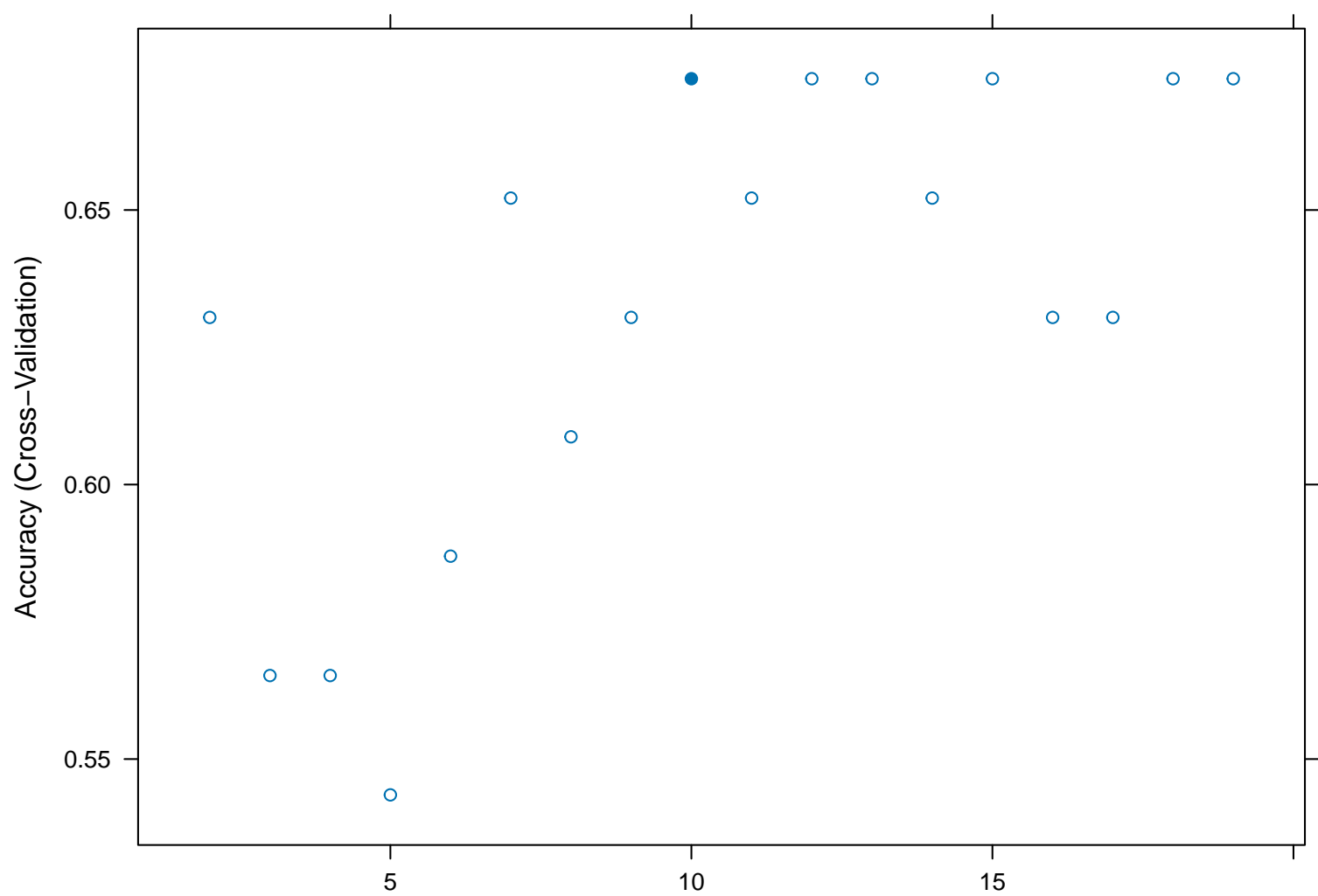

Figure S5 Random Forest screening diagram

Supplement: Supplementary file 1 [file ijms-26-03907-s001.zip › figureS5-RF.pdf]

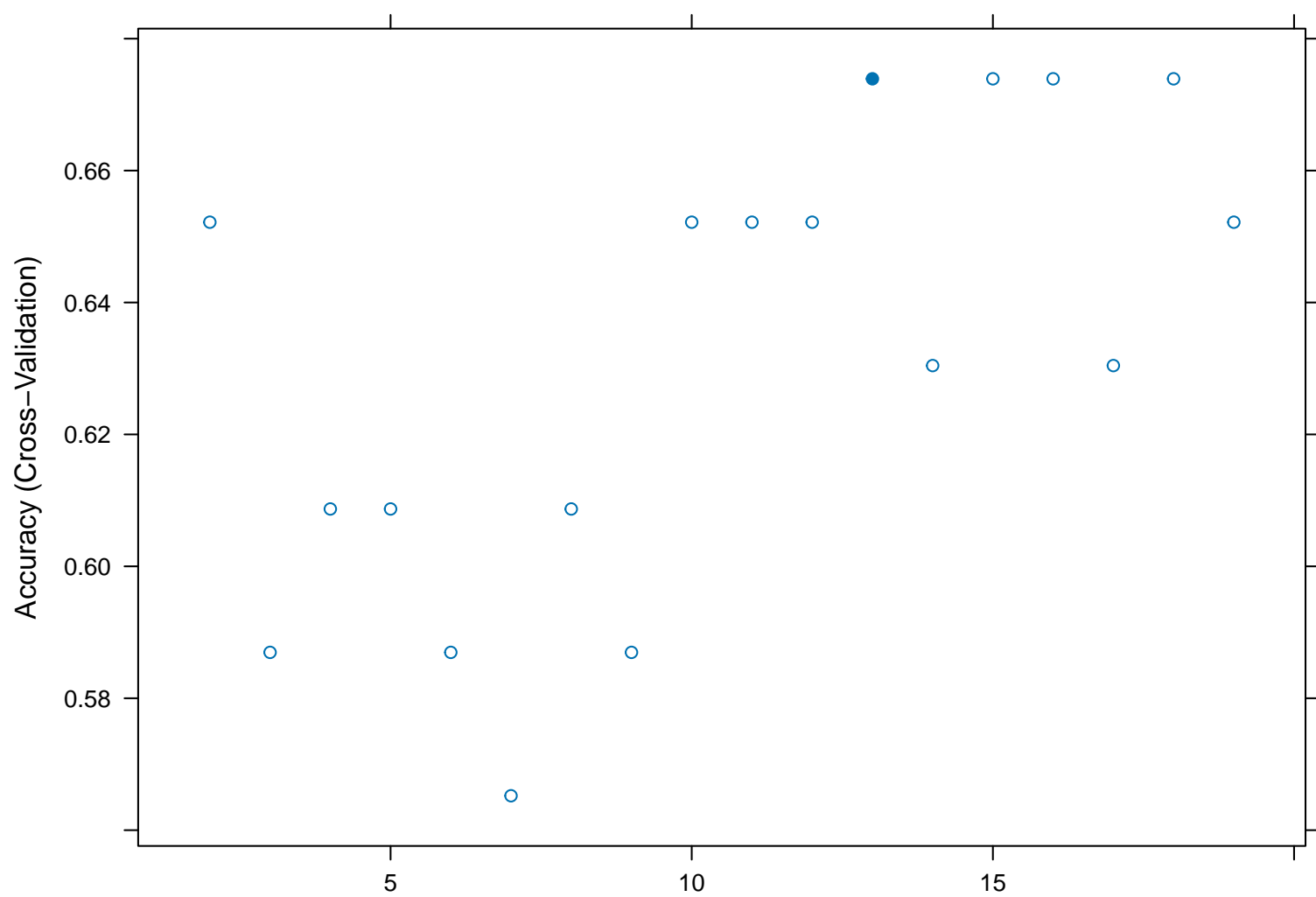

Figure S6 Bayesian screening diagram

Supplement: Supplementary file 1 [file ijms-26-03907-s001.zip › figureS6-bayesian.pdf]

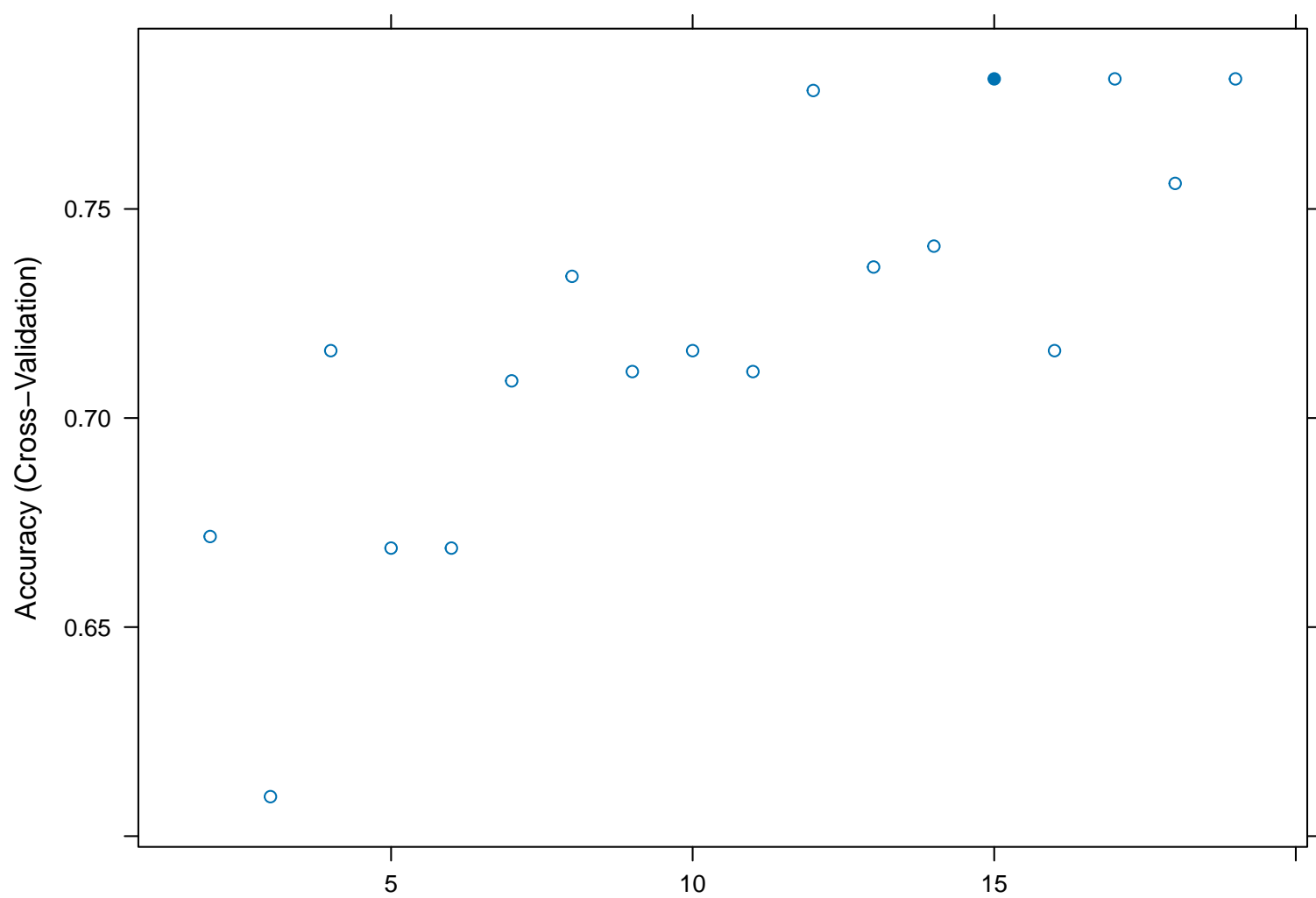

Figure S7 SVM screening diagram

Supplement: Supplementary file 1 [file ijms-26-03907-s001.zip › figureS7-SVM.pdf]

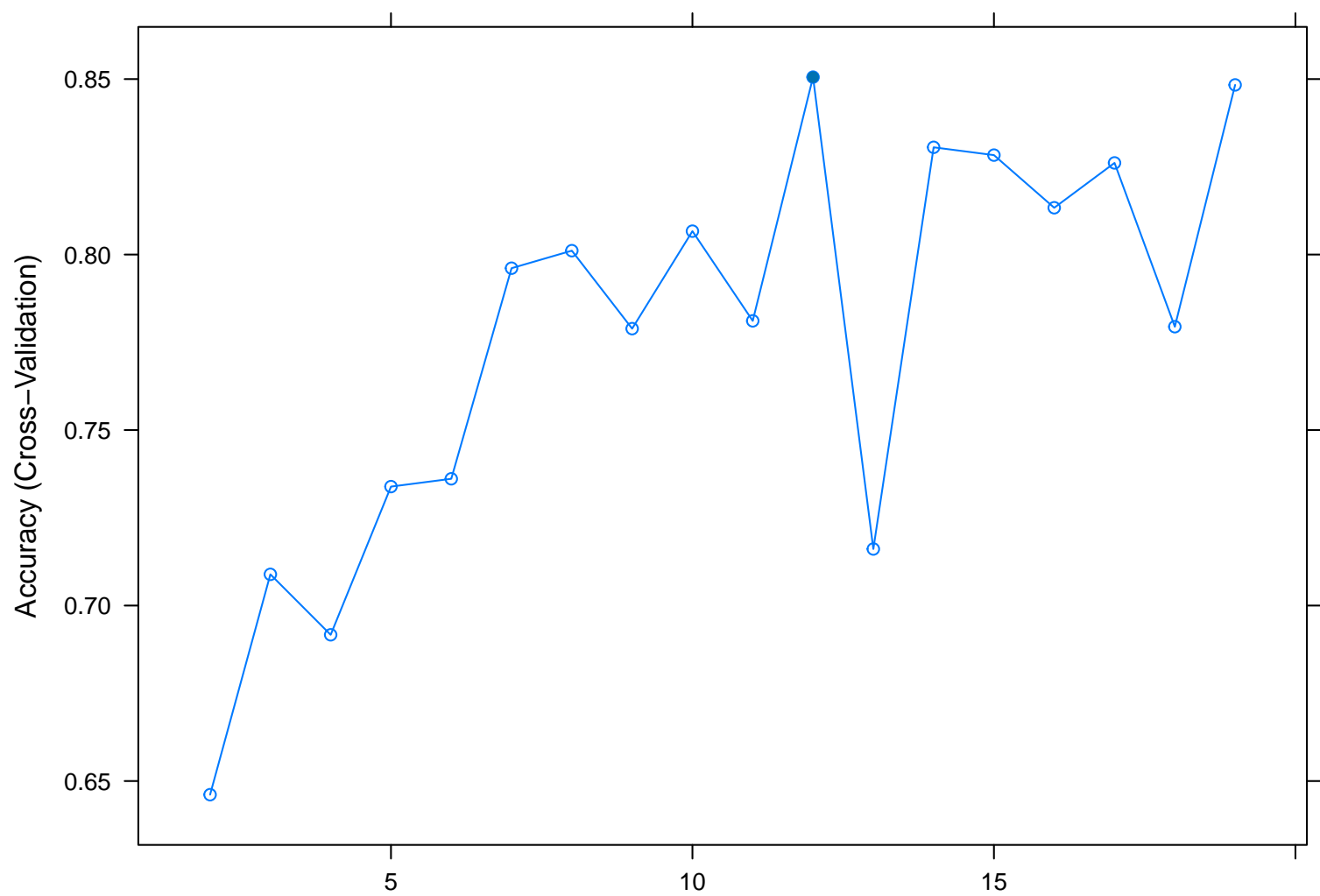

Figure S8 Xgboost screening diagram

Supplement: Supplementary file 1 [file ijms-26-03907-s001.zip › figureS8-xgb.pdf]
